# Comprehensive Transcriptome Analyses Reveal Differential Gene Expression Profiles of *Camellia sinensis* Axillary Buds at Para-, Endo-, Ecodormancy, and Bud Flush Stages

**DOI:** 10.3389/fpls.2017.00553

**Published:** 2017-04-18

**Authors:** Xinyuan Hao, Yajun Yang, Chuan Yue, Lu Wang, David P. Horvath, Xinchao Wang

**Affiliations:** ^1^Tea Research Institute, Chinese Academy of Agricultural SciencesHangzhou, China; ^2^National Center for Tea Improvement, Key Laboratory of Tea Biology and Resources Utilization, Ministry of AgricultureHangzhou, China; ^3^Biosciences Research Laboratory, Sunflower and Plant Biology Research Unit, United States Department of Agriculture-Agricultural Research Service, FargoND, USA

**Keywords:** bud dormancy, transcriptome analysis, tea plant, epigenetic mechanism, phytohormone

## Abstract

Winter dormancy is an important biological feature for tea plant to survive cold winters, and it also affects the economic output of tea plant, one of the few woody plants in the world whose leaves are harvested and one of the few non-conifer evergreen species with characterized dormancies. To discover the bud dormancy regulation mechanism of tea plant in winter, we analyzed the global gene expression profiles of axillary buds at the paradormancy, endodormancy, ecodormancy, and bud flush stages by RNA-Seq analysis. In total, 16,125 differentially expressed genes (DEGs) were identified among the different measured conditions. Gene set enrichment analysis was performed on the DEGs identified from each dormancy transition. Enriched gene ontology terms, gene sets and transcription factors were mainly associated with epigenetic mechanisms, phytohormone signaling pathways, and callose-related cellular communication regulation. Furthermore, differentially expressed transcription factors as well as chromatin- and phytohormone-associated genes were identified. GI-, CAL-, SVP-, PHYB-, SFR6-, LHY-, ZTL-, PIF4/6-, ABI4-, EIN3-, ETR1-, CCA1-, PIN3-, CDK-, and CO-related gene sets were enriched. Based on sequence homology analysis, we summarized the key genes with significant expression differences in poplar and tea plant. The major molecular pathways involved in tea plant dormancy regulation are consistent with those of poplar to a certain extent; however, the gene expression patterns varied. This study provides the global transcriptome profiles of overwintering buds at different dormancy stages and is meaningful for improving the understanding of bud dormancy in tea plant.

## Introduction

Tea plant is a thermophilic perennial evergreen woody plant, generally cultivated between latitudes 45° north and 35° south (Barua, 1969). Its new shoots are processed as tea, a wholesome and healthy beverage that is enjoyed around the world (Trevisanato and Kim, 2000). As an important cash crop, the yield of fresh leaves is a major concern in tea production. “Dormancy” is a key biological event affecting the yield of tea plant over the course of the year. Tea plant has two kinds of “dormancy,” one called ‘Banjhi dormancy’ and the other called ‘winter dormancy.’ Tea plant cultivated at or near the equator usually grows throughout the year if the environmental conditions are favorable. However, a phenomenon of growth suspension known as ‘Banjhi dormancy’ is observed several times in 1 year in unpruned and even harvested tea plants and is recognized as a specific form of ‘endogenous rhythmic growth’ (Tanton, 1981). Tea plants growing at latitudes beyond approximately 16° north or south will halt their growth in winter, and the duration of growth cessation lasts longer with increasing latitudes. This kind of growth cessation is mainly regulated by short day length and low temperature and is called ‘winter dormancy’ (Barua, 1969; Paul and Kumar, 2011). Photoperiod and temperature are crucial factors affecting tea plant dormancy, and tea plant winter dormancy can begin when the photoperiod is less than 11 h 15 min and the minimum temperature falls below 13°C for at least 6 weeks (Barua, 1969, 1989). During the bud set and dormancy release periods, dynamic changes in endogenous hormones are important factors affecting bud growth status. Free gibberellin (GA) and free auxin (indole-3-butyric acid [IAA]) are maintained at extremely low levels during dormancy initiation and deep dormancy but are stimulated in tea shoots prior to dormancy release (Nagar and Kumar, 2000; Nagar and Sood, 2006). Nevertheless, abscisic acid (ABA) shows inverse change patterns during winter dormancy (Nagar, 1996). High putrescine and low spermidine and spermine levels impose dormancy, whereas high spermidine and spermine levels promote dormancy release in tea plant (Kakkar and Nagar, 1997). Phenols and metabolic superoxide contents and cellular damage have been shown to increase during winter dormancy (Nagar, 1996; Vyas and Kumar, 2005). At the molecular level, the genes of alpha-tubulin (*CsTUA*) and histone H3 (*CsH3*) were cloned from tea plant and were up-regulated and down-regulated, respectively, during winter dormancy. Recently, to determine why tea plant is a non-deciduous species, Paul and Kumar (2011) used suppression subtractive hybridization to identify a total of 490 expressed sequence tags (ESTs) showing differential expression in the apical bud and the associated two leaves (two and a bud) between the active growth and winter dormancy phases. In addition, Paul A. et al. (2014) performed RNA-Seq analysis using the same experimental materials and finally identified 5,204 differentially expressed genes (DEGs) out of 24,700 unigenes.

Generally, perennial plants stop active growth and enter dormancy to endure unfavorable environmental conditions in winter. In most woody species, overwintering buds are produced before winter arrives, remain dormant throughout winter and sprout in spring when the external environment becomes favorable. Photoperiod and temperature are the primary environmental cues in dormancy induction; in addition, growth cessation during the dormancy cycle is controlled autonomously in some species (Cooke et al., 2012). Lang (1987) pioneered a classification of dormancy from a physiological viewpoint, namely, paradormancy, endodormancy, and ecodormancy. In paradormancy, growth-promoting environmental or endogenous signals from other plant structures are perceived by the dormant structure, which will resume growth when the signal-producing structure is removed. In endodormancy, a meristem-containing dormant structure perceives growth-promoting environmental or endogenous signals from itself and will not resume growth even if the external adverse factors are removed. In ecodormancy, a growth-competent structure is dormant due to unfavorable external factors, such as nutrients, water and low temperature, and will quickly resume growth in the absence of these unfavorable factors (Lang, 1987; Lang et al., 1987). Rinne et al. (2001) later proposed a new model for dormancy cycling depending on states of cellular communication affected by the accumulation and hydrolyzation of 1,3-beta-d-glucan at the plasmodesmata. Currently, bud dormancy is defined as the inability of a meristem to resume growth under favorable conditions (Rohde and Bhalerao, 2007). A quantitative trait locus (QTL) approach was carried out in poplar and other species to explore the candidate genes involved in bud set and bud flush (Pelgas et al., 2011; Rohde et al., 2011). The explored genes included several *MADS* genes, *PHYTOCHROME B, ABSCISIC ACID INSENSITIVE 1/3, LATE ELONGATED HYPOCOTYL, CONSTANS, FLOWERING LOCUS T, GIGANTEA, PSEUDORESPONSE REGULATOR 5*. These genes were also identified as DEGs through transcriptomic and metabolomic profiling analyses during dormancy transition (Ruttink et al., 2007; El Kayal et al., 2011; Howe et al., 2015). Moreover, the signaling of hormones and their metabolites, such as ABA, cytokinins (CKs), auxin, ethylene and GA, was also important for the regulation of bud dormancy. The roles of epigenetic regulation, including in histone modification, DNA methylation and microRNA, in the bud activity-dormancy cycle were highlighted recently (Cooke et al., 2012; Ríos et al., 2014). In particular, several key genes and microRNAs involved in epigenetic regulation were identified in poplar studies, including *HDA14, SUVR3, HUB2, FIE, CDC48-LIKE, HISTONE1-3, PICKLE*, miRNA156, and miRNA172 (Ruttink et al., 2007; Karlberg et al., 2010; El Kayal et al., 2011; Wang et al., 2011; Howe et al., 2015). The functional discovery of the FT/CO regulatory module in growth cessation made us reconsider the relationship between flowering and dormancy regulation (Bohlenius et al., 2006; Hsu et al., 2011). The dormancy regulation mechanism is complex and variable in different species, especially between evergreen and deciduous species.

Winter dormancy in tea plant is manifested as bud dormancy, and as a typical thermophilic perennial evergreen woody plant, its dormancy mechanism has not been well studied. In this study, we collected axillary buds at the dormancy and sprouting stages to comprehensively study their differential expression profiles by RNA-seq analysis. We also compared the transcriptome analysis results with the latest bud dormancy study in *Populus* with a similar experimental design (Howe et al., 2015). We expect to provide a better understanding of winter dormancy regulation in tea plant through extensive transcriptome analysis.

## Materials and Methods

### Plant Material

The 10-year-old tea cultivar “Longjing 43” (*Camellia sinensis* (L.) O. Kuntze cv. Longjing 43) grown in a field in Hangzhou, Zhejiang Province, China (N30°18′, E120°10′), was used as plant material. The tea plants were provided thorough pest and fertilizer management. We sampled axillary buds from the middle position of the annual branches on different dates between October 2013 and October 2014. Buds collected from adjacent multiple plants were pooled as one sample, and samples collected from three different locations in the field were used as biological replicates. All sampled buds were frozen immediately in liquid nitrogen after being detached from the branches and then stored at -80°C until RNA isolation. The temperature and sunshine duration of each day from September 2013 to April 2014 are shown in Supplementary Material 1-S1.

### RNA Isolation, Library Construction, and Sequencing

RNAs were isolated separately from the samples collected on December 1, 2013, February 14, 2014, March 14, 2014, and June 4, 2014, referring to the CTAB method described by Chang et al. (1993). The appearances of shoot and axillary buds on the above sampling dates are recorded in Supplementary Material 1-S2. The extracted RNAs were treated with DNase I (Invitrogen, Carlsbad, CA, USA) to remove contaminating genomic DNA, and then the RNA quality was verified by 1% gel electrophoresis. High-quality RNAs were treated with a Poly(A) Purist^TM^-MAG Magnetic mRNA Purification Kit (Ambion, Life Technologies, Lithuania) following the manufacturer’s protocol to enrich mRNA. The enriched mRNAs were used to prepare cDNA libraries using a TruSeq RNA Sample Prep Kit (Illumina, San Diego, CA, USA) following the manufacturer’s instruction. The fragment length distributions of the prepared cDNA libraries were examined with an Agilent 2100 Bioanalyzer (Agilent Technologies, Palo Alto, CA, USA). A total of twelve cDNA libraries were sequenced using a whole lane of an Illumina HiSeq 2000 sequencer after qRT-PCR normalization.

### *De Novo* Assembly and Functional Annotation

After sequencing data were acquired, the adapter sequences were removed, and low-quality reads were removed by the “Sickle” quality-based-trimming program^1^ with default settings. The coverage of repeat reads was decreased using the “Trinity normalize by Kmer coverage r2103-08-14” program with parameters of “K-mer size = 25, maximum coverage = 30, maximum pct of mean for stdev of coverage across read = 100”^2^. Finally, the Trinity (v2.2.0) pipeline was run on Kmer-normalized reads. The *de novo* assembly transcripts were evaluated by “Compute Contig Statistics” and “BUSCO” in the Discovery Environment of CyVerse^3^. By BlastX analysis (BLAST 2.2.30+) with the NR database^4^ and protein databases of *Populus* (Ptrichocarpa_210_v3.0.protein.fa and Ptrichocarpa_210_v3.0.annotation_info.txt) and *Arabidopsis* (Athaliana_167_TAIR10.protein.fa and Athaliana_167_TAIR10. annotation_info.txt) released as parts of Phytozome v11.0.8^5^, the best hits (with a significant *E*-value of < 1e^-5^) were assigned to the assembled transcripts of tea plant.

### Gene Expression Pattern and Identification of Differentially Expressed Genes (DEGs)

Using the *de novo* assembled transcripts as reference sequences, transcript abundance from different sets of RNA-seq reads were quantified using the RSEM v1.2.11 program (Li and Dewey, 2011). The expression patterns and “PPEE” values of each gene and contig were estimated by EBSeq v1.1.5, a useful R package for differential expression analysis of RNA-seq data (Leng et al., 2015). “PPEE” stands for the posterior probability that a gene/transcript is equally expressed. Meanwhile, EBSeq also provides the normalized mean count value for each gene/transcript under each condition, which can be used to calculate fold changes between conditions. Finally, a total of 15 expression patterns were generated to describe the expression changes between four specific conditions in this study (Supplementary Material 2). Each row refers to a different expression pattern, and each column gives the expression status of a different condition. Two conditions are equally expressed if and only if their statuses are the same. We summarized the DEGs among the comparisons of Para vs. Endo, Endo vs. Eco, and Eco vs. Flush (Supplementary Material 2).

### Sample Clustering Analysis and Characterization of Bud Dormancy States

A total of 12 samples used in RNA-seq analysis were collected on four different dates. According to the gene expression pattern analysis results, a total of 16,126 DEGs among four different sampling dates were used for clustering analysis. The log2 values of normalized mean counts were used as fold changes of gene/transcript expression level. Using “heatmap.2” in R packages, we made a heat map to show the expression changes of all DEGs between conditions. In this analysis, all DEGs were clustered by the Pearson method, and all samples were clustered by the Spearman method. This information was used to support the dormancy status assessments: paradormant (Para, June 4, 2014), endodormant (Endo, December 1, 2013), ecodormant (Eco, February 14, 2014), and bud flush (Flush, March 14, 2014). To further verify the dormancy states of bud samples on December 1, 2013, and February 14, 2014, we used the following regrowth method. The 9-year-old potted tea cultivar “Longjing 43” grown in the field at the same location where the bud sampling was conducted was used to identify the dormancy status of axillary buds by moving the plants from the field to a greenhouse (14 h of light at 23°C and 10 h of dark at 20°C) after light pruning. Dormancy identification was performed almost every half month from September 2013 to March 2014.

### Gene Set Enrichment Analysis (GSEA) of DEGs

Gene set enrichment analysis was used to identify gene sets that were overrepresented among the DEGs, especially in the comparisons of Para vs. Endo, Endo vs. Eco, and Eco vs. Flush. GSEA is a useful statistical approach for identifying DEG sets (Subramanian et al., 2005). In the above comparisons, we analyzed the up-regulated genes and down-regulated genes separately; in addition, we analyzed the DEGs without considering the direction of change of the individual genes. The GSEA was performed using the Java application GSEA v2.0.13 (Broad Institute, Cambridge, MA, USA); the parameters were set up following the description by Howe et al. (2015). Gene sets were considered statistically significant at an FDR *p*-value of 0.05. Three Gene Ontology (GO) datasets including biological process, cellular component and molecular function were analyzed. The GO terms of tea genes were assigned using the annotation information of Arabidopsis through available functional annotation. Moreover, signaling pathway enrichment analysis was also included. For the Pathway Studio dataset, six sets of genes, including expression targets, miRNA targets, binding partners, protein modification targets, proteins regulating cell processes and disease/cell process-associated proteins, were used.

### Key DEG Identification

We identified the transcription factor genes involved in DEGs using the *Arabidopsis thaliana* Plant Transcription Factor Database v4.0 (PlantTFDB 4.0^6^; Jin et al., 2017). The chromatin-associated genes and phytohormone-associated genes were also identified based on the previous functional annotation and the available classification data in an extensive transcriptome study of *Populus* (Howe et al., 2015). The expression changes (log2 value) of all the identified chromatin-associated and phytohormone-associated genes among the four different developmental phases are shown as heat maps produced by the R program. We estimated the standard deviation (SD) of log2 values of mean count values of samples under four different conditions and then ranked the deviation values. The differentially expressed phytohormone-associated genes with *SD* > 1.5 are shown in the heat map analysis; moreover, the top 60 differentially expressed transcription factor genes with large SDs are shown in the heat map as well.

### Comparison Analysis of DEGs from Poplar and Tea Plant Studies

Recently, extensive transcriptome changes have been investigated during the natural onset and release of vegetative bud dormancy in *Populus* (Howe et al., 2015). To compare the results of transcriptome analyses in *Populus* with this study, we summarized the matching DEGs in both studies by sequence homology. All the DEGs among the paradormancy, endodormancy and ecodormancy phases in this study were analyzed together with the data in Supplemental ‘Data File S1’ provided by Howe et al. (2015). In tea plant, the expression patterns of DEGs during two dormancy transitions of Para/Endo and Endo/Eco were identified following the standards described by Howe et al. (2015). The difference in the significance and direction of expression during transitions was identified based on previous pattern analysis in Supplementary Material 2.

### Gene Expression Validation by qRT-PCR

A total of 15 DEGs were selected from different expression patterns for qRT-PCR validation. Five micrograms of the total RNA used in the previous RNA-Seq library construction was used for cDNA synthesis. A DNase I kit (Invitrogen, Carlsbad, CA, USA) and a SuperScript III First-Strand Synthesis System (Invitrogen, Carlsbad, CA, USA) were used for cDNA synthesis following the exact instructions of the kits. The products were diluted 20-fold with ultrapure water and used as templates in qRT-PCR analysis on a ViiA^TM^ 7 Real-Time PCR System (Thermo Fisher Scientific, USA). The primer information for qRT-PCR is listed in Supplementary Material 3-S1. qRT-PCR reactions were set up with 10 μl of 2 X SYBR^^®^^ Select Master Mix (4472908, Applied Biosystems^^®^^, USA), 1 μl of forward primer (10 pM/μl), 1 μl of reverse primer (10 pM/μl), 1 μl of cDNA template and 7 μl of water with the following program: 95°C for 2 min and 50 cycles of 95°C for 10 s, annealing at 60°C for 10 s, and elongation at 72°C for 40 s. A heat dissociation protocol from 55 to 95°C was added to detect the specificity of primers. Each reaction was performed in triplicate, and *PTB* was chosen as a reference gene (Hao et al., 2014). The expression levels of tested genes were calculated by the delta-delta Ct method. For RNA-Seq analysis, we calculated the log2 value using the average of mean count values from three biological replicates and used it as fold change in expression. The expression levels of each detected transcript under different conditions were normalized to the paradormancy phase. The expression levels detected by RNA-Seq and qRT-PCR are shown in the same bar chart.

## Results

### Developmental Phase Assignment to Sampled Axillary Buds

In this study, the axillary buds collected on December 1, 2013, February 14, 2014, March 14, 2014, and June 4, 2014, were used for RNA-Seq analysis. The appearances of shoots and axillary buds on the above sample collection dates are recorded in Supplementary Material 1. In June, without pruning, tea plants had already produced long shoots after sprouting in the spring. In practice, regrowth always appears after pruning at this time, therefore the axillary buds collected at June were in paradormancy. On March 14, the ambient temperature rapidly increased (Supplementary Material 1-S1), and the dormant buds entered flush after a long, cold winter. On December 1, the axillary buds had undergone months of dormancy induction in early winter after bud set and should have been in endodormancy. On February 14, the dormant axillary buds had already endured a long period of cold, but the outside temperature was still fairly low. The axillary buds should have been in ecodormancy at that time.

**FIGURE 1:**
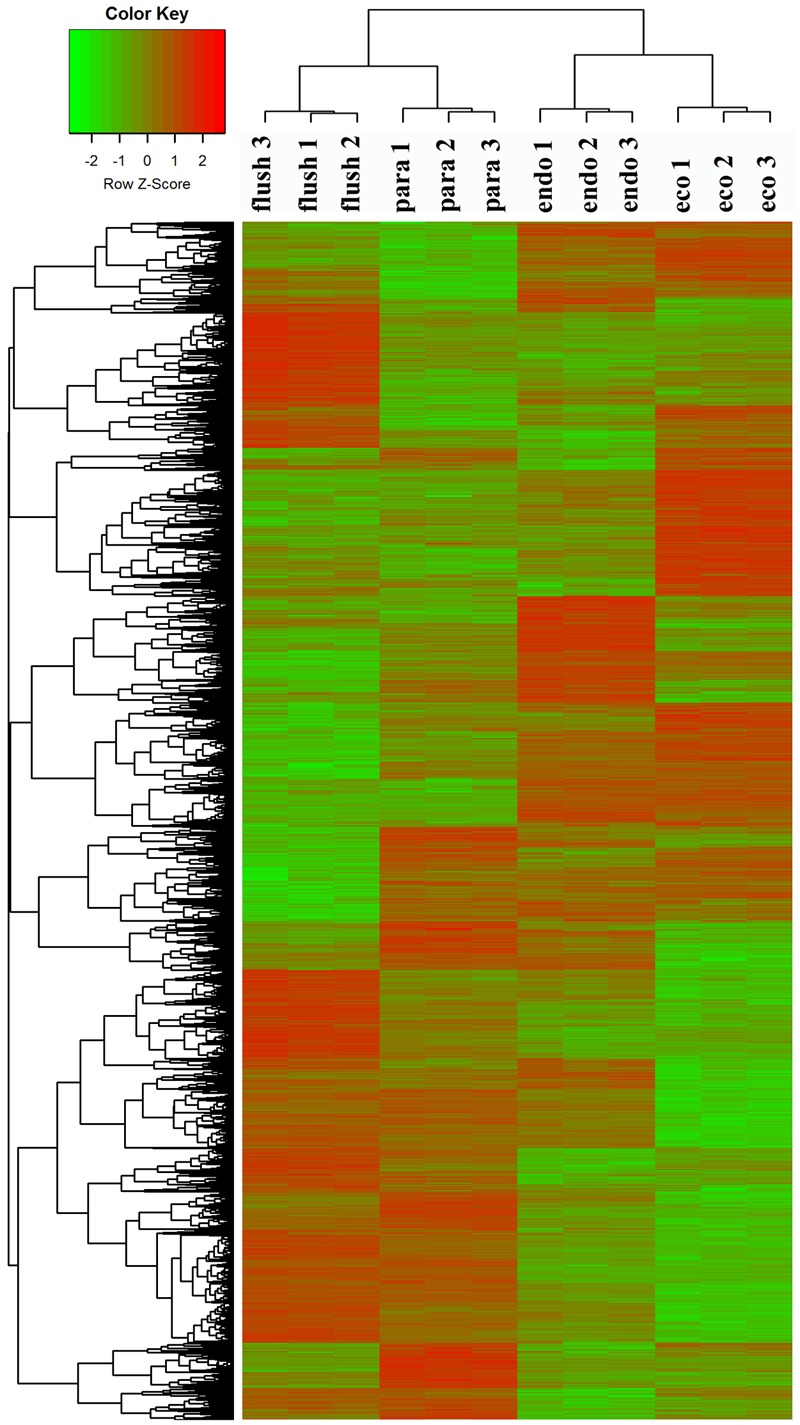
**Clustering analysis of differentially expressed genes under different conditions: paradormant (Para), endodormant (Endo), ecodormant (Eco), and bud flush (Flush).** Clustering was based on the relative expression of 16,125 transcripts with differential expression among the four detect conditions (Para, Endo, Eco, and Flush). Red indicates high relative gene expression and green indicates low relative gene expression.

To confirm the dormancy status assignment on collected buds, we performed regrowth experiments (Supplementary Material 1-S3). The time to bud flush by the plants collected on February 14 (11 days) was more than that of buds from pruning on the eve of bud flush (March 7th) to “one bud and two leaves” (8 days). Likewise, significantly more days (about 28 days) to bud flush were required by the plants collected on December 1. Therefore, the axillary buds collected on December 1, 2013, February 14, 2014, March 14th, 2014, and June 4th, 2014, were in likely in endodormancy (Endo), ecodormancy (Eco), bud flush (Flush), and paradormancy (Para), respectively. Clustering analysis of DEGs showed that the samples collected at different dates were classified into different group (**Figure 1**), indicates that the employed axillary buds were in different developmental phases. This further validated our assumption.

### RNA-seq and *De Novo* Assembly

The total RNAs extracted from more than twelve axillary bud samples were used for RNA-Seq analysis. In total, twelve RNA-Seq libraries were constructed and sequenced on an Illumina HiSeq 2000 platform. Approximately 70 GB of raw data were harvested (Submission number in NCBI: SRR5040773 to SRR5040784), and more than 60 GB of clean data were obtained after quality-based trimming. In total, 313,388 transcripts were produced after *de novo* assembly by Trinity (**Table 1**). The average length of all transcripts was 849 bp, and N50 (length for which half of the total bases are in contigs of this length or longer) was 1538 bp.

**Table 1:** Statistical results of the RNA-seq *de novo* assembly.

Count	Sum_length	N50	Min_length	Max_length	Ave_length	Sd_length	%Completeness
313388	2.66E+08 bp	1538 bp	201 bp	19,609 bp	849 bp	934 bp	95.2

### Functional Annotation and DEG Analysis

All transcripts were annotated by BlastX using the NR database, *Arabidopsis* protein database and *Populus* protein database with 50.60, 38.90, and 37.53% of the transcripts annotated, respectively. The four different conditions produced a total of 15 expression patterns (Supplementary Material 2). Only the transcripts assigned as “pattern 1” showed no difference in expression among the four conditions. In this study, we only focused on the DEGs in the comparisons of Para vs. Endo, Endo vs. Eco, and Eco vs. Flush. The expression patterns involved in the above comparisons are listed in Supplementary Material 2, and the DEGs are listed on the following pages. The number of DEGs involved in these comparisons is shown in **Table 2**. We used hierarchal clustering of expression patterns to group all transcripts (16,125 in total; pattern 2 to pattern 15) by different conditions (**Figure 1**). The biological replicates collected at each time point had very close expression levels and clustered together. As expected, the samples collected on different dates were clustered into different branches. All the DEGs among the four different conditions that had similar expression patterns were clustered as well. The DEGs clustered into multiple groups that showed clearly different expression patterns under different conditions. These results are consistent with the previous developmental phase assignment.

**Table 2:** The number of differentially expressed genes among the comparisons of Para vs. Endo, Endo vs. Eco, and Eco vs. Flush.

	Para vs. Endo	Endo vs. Eco	Eco vs. Flush
Down-regulated genes	4677	4389	7591
Up-regulated genes	5628	5393	6388
Differentially expressed genes in total	10,305	9782	13,979

### Validation of RNA-seq by qRT-PCR

To validate RNA-seq, we performed cDNA synthesis using the total RNAs for RNA-seq and used the cDNA as a template for qRT-PCR. The expression levels of 15 selected transcripts were detected under four different conditions. Most selected transcripts were found through RNA-Seq analysis to have different expression patterns. The expression results are shown in Supplementary Material 3-S2. The 15 selected transcripts had similar expression patterns between RNA-Seq and qRT-PCR, suggesting reliable expression data by RNA-Seq.

### Gene Set Enrichment Analysis (GSEA)

During the DEG analysis, many gene/transcripts were significantly different at the RNA level. It is difficult to identify the major pathways or metabolic processes responsible for axillary bud development regulation by investigating individual genes. In this study, DEG sets were estimated by the GSEA method, which is a powerful tool for analyzing the expression of large numbers of genes (Subramanian et al., 2005; Howe et al., 2015). Using GSEA, GO term enrichment, and Pathway Studio, signaling pathway analyses were carried out according to gene set enrichment identification. Detailed information is provided in Supplementary Materials 4-S1–S3.

### GO Term Enrichment Analysis

**FIGURE 2:**
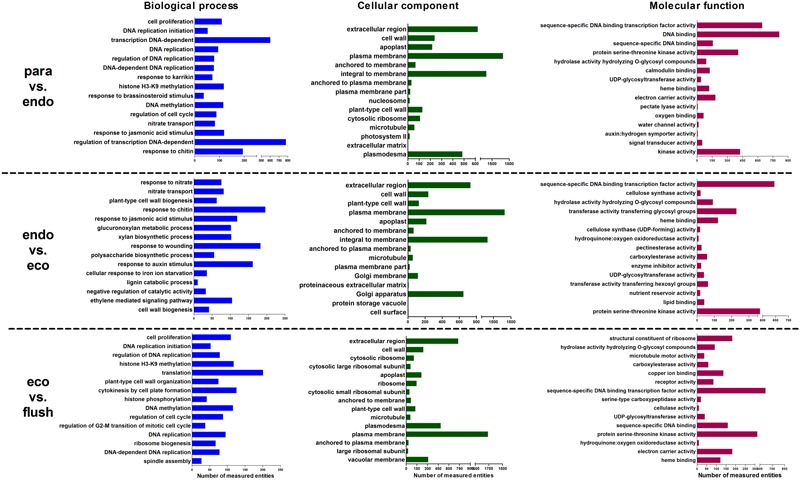
**Gene ontology term enrichment analysis based on GSEA analysis of DEGs in detected comparisons: the top 15 enriched GO terms are listed.** Paradormant (Para), endodormant (Endo), ecodormant (Eco), and bud flush (Flush).

We analyzed the enriched gene sets based on GO category including biological process, cellular component and molecular function. The up-regulated, down-regulated, and up-/down-regulated transcripts were considered separately. More GO terms were down-regulated in Para vs. Endo and Endo vs. Eco than in Eco vs. Flush, with a *p-*value < 0.001 (Supplementary Materials 4-S1–S3). Without considering the direction of change of the individual transcripts, the top 15 enriched GO terms in the biological process, cellular component and molecular function categories among the three comparisons are shown in **Figure 2**. We also identified the GO term enrichment analysis while limiting the gene sets according to the direction of gene expression, the results of enrichment analysis according to the up- or down-regulated genes provided additional insights into the changes that occurred in axillary buds (Supplementary Materials 4-S1–S3).

### Differential Expression of Chromatin-associated Genes

**FIGURE 3:**
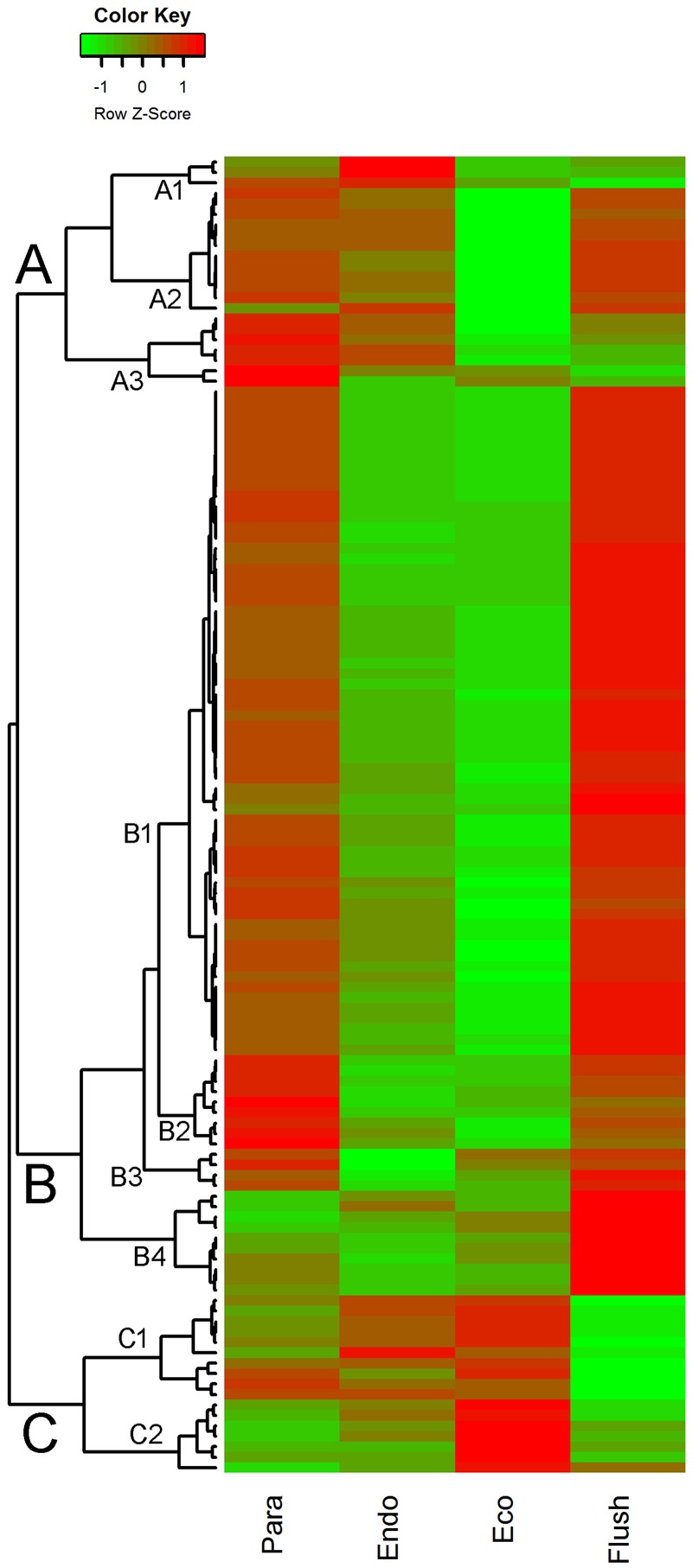
**Expression profile cluster analysis of the chromatin-associated genes with significant difference in expression.** Clustering was based on the relative expression of chromatin-associated genes with differential expression among the four detect conditions: paradormant (Para), endodormant (Endo), ecodormant (Eco), and bud flush (Flush). Red indicates high relative gene expression and green indicates low relative gene expression.

In GO term analysis, the terms ‘histone lysine methylation,’ ‘regulation of DNA replication,’ ‘DNA methylation,’ and ‘DNA replication’ were significantly enriched, especially in comparisons of Para vs. Endo and Eco vs. Flush. This result indicates that the chromatin-associated genes may play important roles in regulating axillary bud dormancy formation and release. In total, we identified 127 chromatin-associated genes with differential expression among different developmental phases (Supplementary Material 5). The expression of these genes at each phase is shown in **Figure 3**. The gene sets related to chromatin in GSEA are listed in Supplementary Materials 4-S5–S7.

#### Chromatin-associated Gene Sets

Aside from the gene sets assigned in GO terms, chromatin-associated gene sets were also enriched in Pathway Studio analysis. In particular, the terms ‘binding partners of HD1 (histone deacetylase 1),’ ‘neighbors of maintenance of DNA methylation,’ ‘neighbors of DNA methyltransferases,’ and ‘neighbors of MET1 (methyltransferase 1)’ were down-regulated in Para vs. Endo. The terms ‘expression targets of histone H3,’ ‘binding partners of helicase,’ and ‘neighbors of N-acetyltransferase’ were enriched in Endo vs. Eco without considering the direction of DEGs. Moreover, the terms ‘binding partners of histone H3,’ ‘neighbors of MET1’ and ‘neighbors of maintenance of DNA methylation’ were enriched in Eco vs. Flush.

#### Chromatin-associated Genes

The expression patterns of chromatin-associated genes were mainly classified into groups A, B and C, and several subgroups were further assigned. Out of 127 DEGs, 87 were classified into group B. Except for subgroup B4, the DEGs involved in group B were significantly down-regulated in buds at endodormancy and ecodormancy compared with expression in the paradormancy and bud flush phases. The group A genes were significantly down-regulated in ecodormancy; in contrast, the group C genes were clearly up-regulated in ecodormancy. Detailed information about chromatin-associated genes is presented in Supplementary Material 5. In group B, the top five DEGs were histone-lysine N-methyltransferase (comp88317_c0_seq2; histone-associated gene), HTA7 (comp100738_c0_seq1, comp76911_c0_seq1; histone H2A 7), CHR32 (comp92368_c0_seq1; helicase protein with RING/U-box domain), and chromatin-associated gene (comp77433_c0_seq1; chromatin accessibility complex protein 1); the first two genes had expression pattern 15 (significantly differential between each comparison), and the other three genes had expression pattern 9 (significantly differential between Para vs. Endo and Eco vs. Flush). The top three DEGs in group C were FLT1 (comp61866_c0_seq1; PEBP (phosphatidylethanolamine-binding protein) family protein), HMGB13 (comp91822_c0_seq1; HMG (high mobility group) box protein), and HFO8 (comp93722_c0_seq7; histone superfamily protein). All of these genes were in subgroup C1. In group A, two of the top three DEGs were in subgroup A2, namely, DNG1 (comp96904_c0_seq1; HhH-GPD base excision DNA repair family protein) and ABHF10 (comp97148_c0_seq4; soluble epoxide hydrolase). The other gene was in subgroup A3, namely, AGO4 (comp37668_c0_seq1; Argonaute family 4 protein).

### Differential Expression of Transcription Factor Genes

**FIGURE 4:**
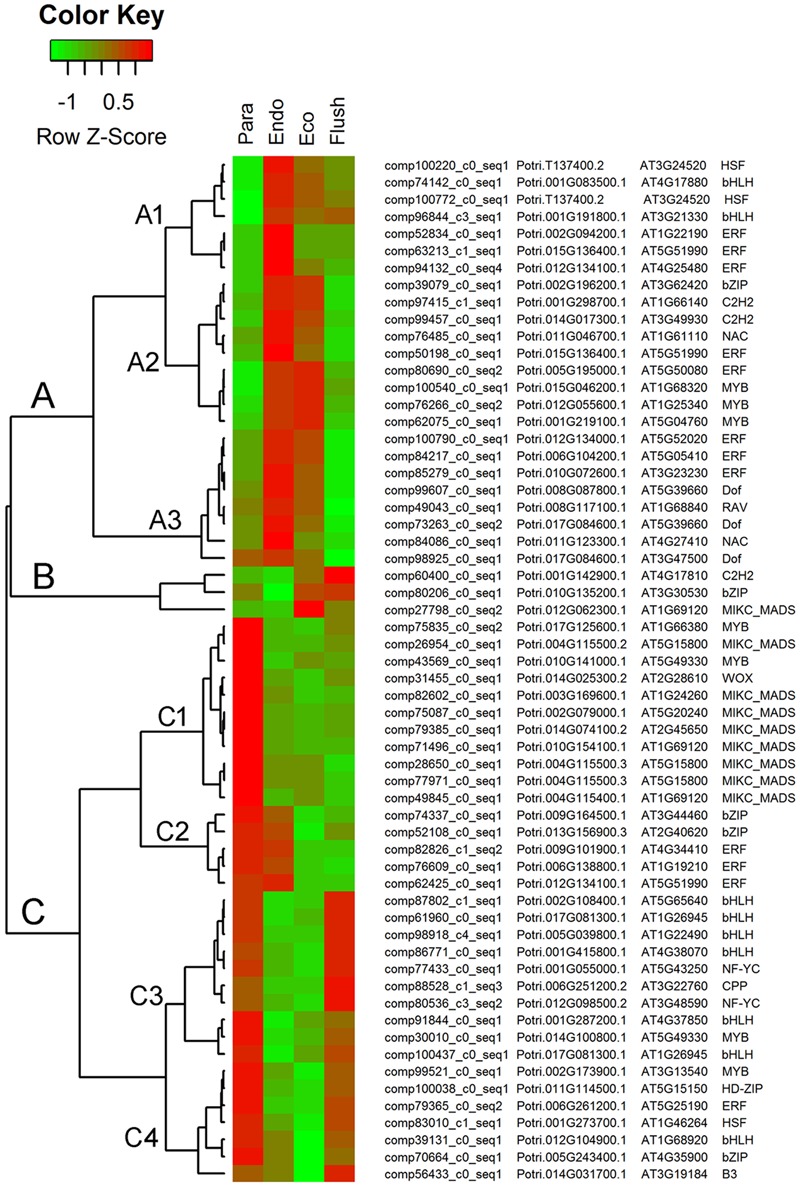
**Expression profile cluster analysis of the transcription factors with significant difference in expression.** Clustering was based on the relative expression of transcription factors with differential expression among the four detect conditions: paradormant (Para), endodormant (Endo), ecodormant (Eco), and bud flush (Flush). The information listed on the right of heat map are the transcripts’ name in this study, the transcript’s annotation by poplar protein database, the transcript’s annotation by *Arabidopsis* protein database and the symbols of transcription factors. Red indicates high relative gene expression and green indicates low relative gene expression.

The GO term of ‘sequence-specific DNA binding transcription factor activity’ contained the largest number of entities in the molecular function catalog in GSEA. Therefore, we identified all the transcription factors in DEGs among the four conditions. In total, 798 transcription factors were identified from DEGs (Supplementary Material 6), and we chose the top 60 differentially expressed transcription factors and show their expression levels in **Figure 4**.

#### Transcription Factor Gene Sets

Pathway Studio analysis let us examine the gene sets enriched in particular nodes of signaling pathways. Many gene sets related to transcription factors were enriched in the three comparisons (Supplementary Materials 4-S5–S7). In Para vs. Endo, transcription-factor-related gene sets were significantly up-regulated, such as ‘expression targets of SFR6 (sensitive to freezing 6),’ ‘expression targets of LHY (late elongated hypocotyl),’ ‘expression targets of ZTL (zeitlupe),’ ‘expression targets of ABI4 (ABA insensitive 6),’ ‘expression targets of ELF3 (early flowering 3),’ ‘binding partners of AHP1 (histidine-containing phosphotransmitter 1),’ ‘binding partners of MADS box protein’ and ‘binding partners of PHYB,’ while more gene sets were down-regulated, such as ‘expression targets of LFY,’ ‘expression targets of BRI1 (brassinosteroid insensitive 1),’ ‘expression targets of SVP,’ ‘expression targets of PIF4 (phytochrome interacting factor 4),’ ‘expression targets of PIL6 (phytochrome interacting factor 3-like 6),’ ‘expression targets of EIN3 (ETHYLENE-INSENSITIVE 3),’ ‘expression targets of SHY2 (SHORT HYPOCOTYL 2),’ ‘neighbors of CAL’ and ‘expression targets of CCA1 (circadian clock associated 1).’ Similarly, in Endo vs. Eco, more transcription-factor-related gene sets were significantly down-regulated, such as ‘binding partners of CESA 1 and 6 (cellulose synthase 1 and 6),’ ‘neighbors of CAL,’ ‘neighbors of EIN2 (ETHYLENE-INSENSITIVE 2),’ ‘expression targets of ETR1 (ETHYLENE RESPONSE 1),’ ‘binding partners of IRX1 and 3 (irregular xylem 1 and 3),’ ‘binding partners of cellulose synthase (GDP-forming),’ ‘binding partners of CDK (cyclin-dependent kinase),’ ‘expression targets of MYC2,’ ‘neighbors of PIN3 (PIN-FORMED 3),’ ‘expression targets of BRI1’ and ‘expression targets of bZIP transcription factor.’ When the ecodormant buds entered bud flush, the gene sets of ‘binding partners of CDK,’ ‘expression targets of HB-8 (homeobox-8),’ ‘expression targets of BRI1,’ ‘binding partners of CDC2 (cell division control 2),’ ‘expression targets of PIF4,’ ‘binding partners of basic-helix-loop-helix protein (bHLH),’ ‘expression targets of PIL6,’ ‘neighbors of CAL,’ and ‘binding partners of CO (CONSTANS)’ were significantly up-regulated.

#### Transcription Factor Genes

The top 60 transcription factor DEGs shown in **Figure 4** were clustered in groups A, B, and C. Each group contains subgroups. In group A, the DEGs were significantly up-regulated in the endodormancy and ecodormancy phases. The major transcription factors involved in this group were ‘ERF (ethylene responsive element binding factor),’ ‘MYB (myb domain protein),’ ‘Dof (DNA binding with one finger)’ and ‘bHLH.’ In group B, three kinds of transcription factor were up-regulated in the ecodormancy and bud flush phases, namely, ‘C2H2 (C2H2 zinc finger regulators),’ ‘bZIP (basic leucine-zipper),’ and ‘MIKC-MADS.’ In group C, differentially expressed transcription factors in subgroup C1 only had high expression levels in the paradormancy phase. ‘MIKC-MADS’ was the major one in subgroup C1. The transcription factors in subgroup C2 had high expression in the paradormancy and endodormancy phases and were significantly down-regulated in the ecodormancy and bud flush phases. ‘bZIP’ and ‘ERF’ were the two in subgroup C2. The differentially expressed transcription factors involved in subgroups C3 and C4 had similar expression patterns; however, subgroups C3 and C4 had much higher expression levels in the bud flush and paradormancy phases, respectively. ‘bHLH’ was the major transcription factor in subgroup C3, and multiple transcription factors were grouped in C4.

### Differential Expression of Phytohormone-associated Genes

To determine the major signaling pathways involved in regulating dormancy status transition, signaling pathway enrichment analysis was performed (Supplementary Material 4-S4). Phytohormone-associated gene sets were mainly enriched in all three comparisons. In Para vs. Endo, ‘cytokinins (CK) signaling,’ and ‘salicylic acid signaling’ were up-regulated; in contrast, ‘gibberellin (GA) signaling’ and ‘plant growth auxin signaling’ were down-regulated. In Endo vs. Eco, ‘cytokinins signaling,’ ‘plant growth auxin signaling,’ and ‘stress ABA signaling’ were up-regulated, while ‘salicylic acid signaling’ and ‘jasmonic acid, ethylene, and salicylic acid crosstalk signaling’ were significantly down-regulated. In Eco vs. Flush, ‘gibberellin signaling’ was down-regulated. These indicated that phytohormones play important roles in dormancy transition regulation. Furthermore, phytohormone-associated genes were identified in all DEGs among the four different conditions. In total, 38 ABA-associated genes, 35 GA-associated genes, 119 auxin-associated genes, 33 CK-associated genes, 31 brassinosteroid (BR)-associated genes, 41 ET-associated genes, and 31 JA-associated genes were identified. The expression patterns of these hormone-associated genes are listed separately in Supplementary Materials 7-S1–S7.

#### Phytohormone-associated Gene Sets

Pathway Studio gene set analysis further showed that many phytohormone-associated gene sets were enriched across different comparisons. The auxin-associated gene sets ‘neighbors of auxin metabolism’ and ‘neighbors of auxin’ were up-regulated in Para vs. Endo and Eco vs. Flush. In contrast, the gene sets related to auxin polar transport were down-regulated in Endo vs. Eco, such as ‘neighbors of PIN3,’ and ‘neighbors of auxin polar transport.’ The ABA-related gene sets of ‘expression targets of ABI4 (ABA insensitivity 4)’ and ‘neighbors of ABA’ were down-regulated in Eco vs. Flush, and ‘expression targets of ABI4’ was up-regulated in Para vs. Endo. GA-associated gene sets were mainly enriched in Endo vs. Eco and Eco vs. Flush. The ethylene-associated gene sets ‘neighbors of EIN2/3 (ethylene insensitivity 2/3)’ and ‘expression targets of ETR1 (ethylene response 1)’ were down-regulated in Endo vs. Eco. The brassinosteroid-associated gene sets ‘expression targets of BRI1 (brassinosteroids insensitivity 1),’ and ‘neighbors of brassinosteroids’ were down-regulated when the bud status transitioned into endodormancy and ecodormancy; interestingly, they were up-regulated in the bud flush phase. The CK-associated gene set ‘neighbors of cytokinin content’ was down-regulated in Para vs. Endo, while ‘neighbors of cytokinin’ and ‘cytokinin metabolism’ were up-regulated in Endo vs. Eco.

#### Phytohormone-associated Genes

**FIGURE 5:**
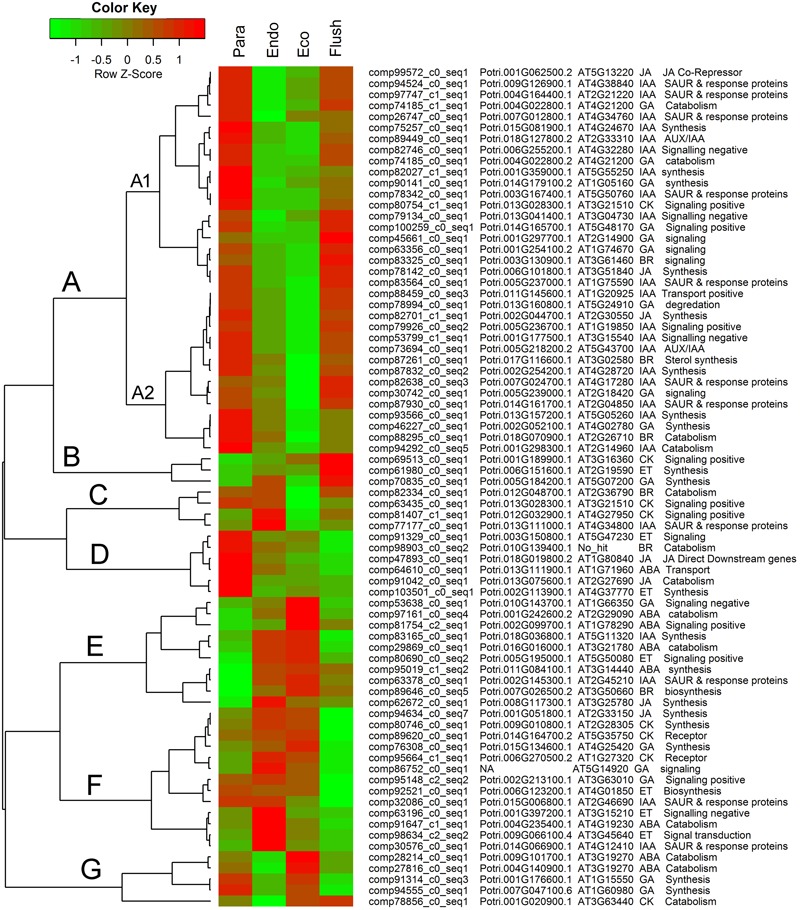
**Expression profile cluster analysis of phytohormone–associated genes with significant difference in expression.** Clustering was based on the relative expression of phytohormone–associated genes with differential expression among the four detect conditions: paradormant (Para), endodormant (Endo), ecodormant (Eco), and bud flush (Flush). The information listed on the right of heat map are the transcripts’ name in this study, the transcript’s annotation by poplar protein database, the transcript’s annotation by *Arabidopsis* protein database, hormone and hormone function. Red indicates high relative gene expression and green indicates low relative gene expression. ABA, abscisic acid; BR, Brassinosteroid; CK, cytokinin; ET, ethylene; GA, gibberellin; IAA, indole-3-butyric acid, and JA, jasmonic acid.

Additionally, we summarized the phytohormone-associated genes with *SD* > 1.5 (**Figure 5**). The expression patterns of these genes were mainly grouped into groups A to G, and group A contained subgroups A1 and A2. Group A had the largest number of DEGs, which all showed high expression levels in the paradormancy and bud flush phases and had low expression levels in the endodormancy and ecodormancy phases. Genes in subgroup A1 showed much lower expression in endodormancy, and genes in subgroup A2 showed much lower expression in ecodormancy. The DEGs in group A were majorly engaged in IAA and GA signaling or synthesis/catabolism pathways. Three DEGs (comp69513_c0_seq1, comp61980_c0_seq1, comp70835_c0_seq1) involved in CK-, ET- and GA–associated pathways were in group B, which showed fairly high expression in the bud flush phase. In group D, the DEGs only had high expression in the paradormancy phase. The members of group D included two ET-associated genes (comp91329_c0_seq1, comp103510_c0_seq1), two JA-associated genes (comp47893_c0_seq1, comp91042_c0_seq1), a BR-associated gene (comp98903_c0_seq2), and an ABA-associated gene (comp64610_c0_seq1). The DEGs in group E had very low expression in paradormancy; however, the gene expression was significantly up-regulated in endodormancy and ecodormancy. More members in this group were ABA-associated genes (comp97161_c0_seq1, comp81754_c2_seq1, comp29869_c0_seq1, comp95019_c1_seq2). The DEGs in group F showed much lower expression in the bud flush phase, and CK-, ET- and GA-associated genes were mainly involved. In group G, the genes were down-regulated in endodormancy and clearly up-regulated in ecodormancy; this group comprised two ABA-associated genes (comp28214_c0_seq1, comp27816_c0_seq1), two GA-associated genes (comp91314_c0_seq3, comp94555_c0_seq1) and a CK-associated gene (comp78856_c0_seq1). Interestingly, the genes in group C showed reverse expression patterns between endodormancy and ecodormancy. Two CK-associated genes (comp63435_c0_seq1, comp81407_c1_seq1), a BR-associated gene (comp82334_c0_seq1) and an IAA-associated gene (comp77177_c0_seq1) were involved.

### Comparisons of Key DEGs between *Camellia sinensis* and *Populus* during Endodormancy Formation and Release

In extensive transcriptome analysis of vegetative buds in *Populus*, 1,362 DEGs were identified during the dormancy transitions Para/Endo and Endo/Eco. By functional annotation, 417 DEGs in dormancy transitions from Para to Eco in tea plant were matched to 394 out of 1362 DEGs from the poplar study, in which two or more DEGs in tea plant could be matched to same gene in poplar (Supplementary Material 8). The chromatin-associated genes, phytohormone-associated genes, dormancy-related QTL neighbors and transcription factors were examined in the poplar study. Therefore, we statistically analyzed the number of DEGs involved in the above gene groups during endodormancy onset and release (**Table 3**). In total, 6 out of 13 chromatin-associated genes were enriched in both the poplar and tea plant studies. IAA and JA were the top two hormone-associated signaling pathways enriched by large numbers of DEGs in both studies. In poplar, more DEGs were located in dormancy-related QTLs of LG3, LG5, LG8a and LG8b. Similarly, relatively more DEGs from tea plant were matched to the dormancy-related QTLs. Many DEGs were identified as transcription factors in both the poplar and tea plant studies. In particular, ‘MYB’ was the most enriched transcription factor in both studies. ‘ERF,’ ‘bHLH,’ ‘C2h2,’ ‘WRKY’ and ‘HD-ZIP’ were enriched in sequence. Although the major enriched terms in the poplar study were also matched by DEGs in the tea plant study, only approximately 60% of terms were matched in total. Additionally, the expression patterns of matching DEGs did not show much consistency between poplar and tea plant.

**Table 3:** Statistical results of the matching differentially expressed genes from the poplar and tea plant studies.

Classification	Name	Number of entities in poplar	Number of entities in tea plant	Classification	Name	Number of entities in poplar	Number of entities in tea plant
Chromatin	ABHF10	1	1	TF	BBR-BPC	1	0
Chromatin	ABHF4	2	0	TF	bHLH	8	4
Chromatin	AGO4	1	1	TF	bZIP	1	0
Chromatin	DNG3	1	0	TF	C2H2	7	3
Chromatin	DNG5	1	0	TF	C3H	2	0
Chromatin	EBP1	1	0	TF	CAMTA	1	1
Chromatin	FLT4	1	1	TF	CO-like	2	1
Chromatin	GTA2	1	1	TF	DBB	1	1
Chromatin	GTB1	1	0	TF	Dof	7	2
Chromatin	HMGA3	1	0	TF	ERF	9	5
Chromatin	PATPA1	1	1	TF	FAR1	1	0
Chromatin	RDR1	1	0	TF	GATA	1	0
Chromatin	RHEL1	1	1	TF	GRAS	4	2
Hormone	ABA	1	0	TF	HB-other	1	0
Hormone	IAA	17	6	TF	HD-ZIP	7	3
Hormone	BR	5	1	TF	HRT-like	1	0
Hormone	CK	3	1	TF	LBD	2	0
Hormone	ET	2	0	TF	MIKC	6	0
Hormone	JA	6	2	TF	MYB	17	5
Hormone	SA	11	1	TF	NAC	3	1
QTL	LG13	6	1	TF	NF-YA	1	0
QTL	LG3	14	4	TF	RAV	1	1
QTL	LG5	12	3	TF	SBP	4	2
QTL	LG6	7	2	TF	SRS	1	0
QTL	LG8a	14	2	TF	TALE	1	0
QTL	LG8b	17	5	TF	TCP	4	2
TF	AP2	3	0	TF	Trihelix	3	2
TF	ARF	4	1	TF	WRKY	6	4
TF	B3	1	0	TF	ZF-HD	2	2

## Discussion

### *De novo* Assembly and DEG Analysis

A total of 12 libraries were constructed in RNA-Seq analysis, and 313,388 transcripts were produced after *de novo* assembly. This transcriptome set was substantially larger than previous dormancy-related RNA-Seq analysis, where only 42,916 assembled sequences were identified from a two leaves and a bud samples (Paul A. et al., 2014). Additionally, our data set appears to be more complete as our average length and N50 of transcripts were up to 849 bp and 1538 bp, respectively, whereas in the previous study, the average length of sequences was approximately 459 bp (Paul A. et al., 2014). Other recent global transcriptome profile analysis on the same tea cultivar reported a total of 127,094 unigenes with a mean length of 355 and an N50 of 506 were obtained using different tea organs as materials, and later a total of 216,831 transcripts, with an average length of 356 bp and an N50 of 529 bp, were harvested from mature leaves (Shi et al., 2011; Wang et al., 2013). In view of the statistical results of our assembly (**Table 1**), 95.2% of conserved eukaryotic genes that were identified as complete or partial in our assembly, therefore we obtained high-quality sequencing and assembly. The large number of transcripts produced in this study could be attributed to the deep RNA sequencing and large genome size of tea plant (Tanaka et al., 2006). The obtained transcripts were subsequently annotated using three different protein databases.

Although expression profile analyses of buds in paradormancy, endodormancy and ecodormancy have been repeatedly reported in other species, little attention has been paid to buds in the sprouting stage (Ruttink et al., 2007; Horvath et al., 2008; Bai et al., 2013). The introduction of the bud flush phase here helps to draw a relatively complete development cycle of axillary buds. In a previous study, a total of 5,204 DEGs were identified when comparing gene expression in actively growing shoots and dormant shoots of tea plant (Paul A. et al., 2014). Compared with previous studies, more DEGs were identified in this study, and all the DEGs were employed in the subsequent GSEA analysis.

### Chromatin-associated Epigenetic Regulation in Bud Dormancy Transition

Aside from transcription factor-mediated expression regulation, DNA methylation, histone modifications, and RNA-based mechanisms are the three main epigenetic mechanisms operating in plant development, and they are particularly prominent in plant dormancy studies (Cooke et al., 2012; Ríos et al., 2014). In this study, chromatin-associated gene set analysis showed that DNA methylation- and histone modification-related terms were enriched in the three comparisons. In particular, the terms associated with methylation maintenance and deacetylation, such as ‘neighbors of maintenance of DNA methylation,’ ‘neighbors of MET1’ and ‘binding partners of HD1,’ were down-regulated in Para- vs. Endo-dormancy. In *Arabidopsis, MET1* and *CMT3* are major maintenance-type DNA methyltransferases, and *DRM2* is the major *de novo*-type DNA methyltransferase; histone H3-K9 methyltransferase KYP and the catalytically non-active protein DRM3 are their functionally related proteins (Ashapkin et al., 2016). Reversible histone acetylation and deacetylation at the N-termini of histone tails constitute an important histone modification mechanism playing a crucial role in the regulation of gene activity (Roth et al., 2001). Histone acetyltransferases (HATs) and histone deacetylases (HDACs) catalyze histone acetylation and deacetylation reactions, respectively, which play essential roles in regulation of gene expression in plant development and plant responses to environmental stresses (Liu et al., 2016). Santamaría et al. (2009) measured DNA methylation and acetylation levels genome-wide during bud set and bud burst and discovered their general association with bud dormancy. Recently, RNA-Seq analysis in the apple tree further reported that high expression levels of DNA methyltransferases and histone methyltransferases were detected during the dormancy and fruit set stages (Kumar et al., 2016). In *Picea glauca* after short-day induction, HDAC and CMT-type DNA methyltransferase were down-regulated (El Kayal et al., 2011).

The histone methylation and histone H3 acetylation levels in the promoter or intron regions of *DAM*, a gene involved in dormancy regulation, are associated with dormancy transition (Horvath et al., 2010; Leida et al., 2012; Saito et al., 2015). These findings suggest a significant association between bud development regulation and epigenetic changes, especially in dormancy transitions, that have been observed in other perennials appears to be conserved in the evergreen broad-leafed tea plant as well. In apple buds, *HTA8* and *HTA12* together with *DAM* were identified as major dormancy-related genes (Falavigna et al., 2014). Histone modification was investigated in the *DAM* homolog *PpMADS13-1* from Japanese pear; the loss of histone variant H2A.Z coincided with the down-regulation of *PpMADS13-1* (Saito et al., 2015). The functions of CHR32 and chromatin-associated genes still require further study, but they might play important roles in regulating DNA unwinding and transcription, depending on functional annotation. The genes FLT1, HMGB13 and HFO8 were the top three DEGs in group C1 and showed significantly elevated expression in the ecodormancy stage.

This study also presents expression profiling of all identified differentially expressed chromatin-associated genes. Most chromatin-associated DEGs were grouped into group B. In this group, DEGs had obvious low expression levels at the endodormancy and ecodormancy stages. Histone-lysine *N*-methyltransferase, HTA7, CHR32 and chromatin-associated gene were the most enriched four DEGs in group B. In *Populus* transcriptomic analysis, the GO molecular function gene set of ‘Histone lysine N-methyltransferase’ was also down-regulated from paradormancy to endodormancy (Howe et al., 2015).

Barakat et al. (2012) identified chilling-responsive microRNAs genome-wide in *Prunus persica*, and histone lysine *N*-methyltransferase was listed as an important target of four microRNA families (miR5021, miR164, miR396 and miR2919) in the vegetative to reproductive phase transition of the meristem during winter dormancy. Interestingly, in our gene set analysis (Supplementary Material 4) for the comparison of Para vs. Endo, ‘miRNA targets of MIR164A’ was highly enriched.

The functional identification of the CO/FT module in flowering and seasonal growth cessation regulation in trees was a breakthrough in the study of dormancy (Bohlenius et al., 2006). Methylation of H3K27 and H3K4 in *FT* chromatin regulates its expression level (Jiang et al., 2008; Jeong et al., 2009). Polycomb Repressive Complex 2 (PRC2) and LIKE-HETEROCHROMATIN PROTEIN (LHP1) play roles in the abovementioned methylation regulation mechanism (Turck et al., 2007; Jiang et al., 2008). Histone mark readers MRG1/2 can even modulate *FT* expression through interaction with CO (Bu et al., 2014). The HMGB family is one group of chromosomal high-mobility-group (HMG) proteins in plants and contains a single HMG-box DNA-binding domain (Grasser, 2003). Members of the HMGB family act as versatile modulators of chromatin function, including transcriptional regulation (Grasser et al., 2006). Histone H4 (HFO) is an important carrier of chromatin modifications on the nucleosome core (Iglesias and Cerdan, 2016).

The chromatin-associated DEGs that showed specific low expression at the ecodormancy phase were mainly grouped into group A. DNG1, ABHF10 and AGO4 were the top genes with expression variation. Similarly, ABHF4 and DNG1 were important chromatin-associated genes identified in poplar transcriptomic analysis; these genes had the same expression patterns during dormancy transition (Howe et al., 2015). The only two Argonaute (AGO) genes with significant expression differences were clustered into Group A in this study. AGO proteins are key players in RNA silencing and can bind small non-coding RNAs, control protein synthesis, and affect messenger RNA stability (Hutvagner and Simard, 2008). The gene sets related to miRNA targets were enriched in the three comparisons, and the AGO proteins play important roles in miRNA function execution. According to the gene set enrichment and chromatin-associated DEG analyses, epigenetic mechanisms may play important roles in tea plant bud development and dormancy regulation.

### Key Transcription Factors Involved in Bud Dormancy Regulation

Many phytohormone-associated transcriptome factors were identified among comparisons, and the expression targets or neighbors of GAI, ABI4, MYC2, BRI1, EIN2/3, ETR1, and PIN3 were majorly enriched in gene set analysis. ABA is a phytohormone regulating many aspects of plant growth and development, including tolerance of a variety of environment stresses (And and Giraudat, 1998). Changes in GA metabolism and signaling occur during early dormancy induction and release (Cooke et al., 2012). ABI4 is an important regulator in seed dormancy by regulating the biogenesis of ABA and GA in *Arabidopsis* (Shu et al., 2013). MYC2 and other ABA-related transcription factors are up-regulated after 1–2 weeks of short-day induction (Ruttink et al., 2007). Aside from ABA and GA, the roles of BR in promoting germination have attracted more attention in recent seed dormancy studies (Steber and McCourt, 2001). In this study during the bud flush stage, BRI1-related expression targets were mainly up-regulated, which indicated the possible roles of BR in bud sprouting regulation. Implicating BR signaling in tea plant bud dormancy transitions provides further evidence for a potential role for BR first noted in poplar (Howe et al., 2015). Ethylene metabolism- and signaling-related gene sets (expression targets of EIN2/3, ETR1) were enriched in comparison of Endo vs. Eco. Moreover, 12 ERFs were included in the top 60 differentially expressed transcription factors. Based on the expression profiling of the top 60 transcription factors with different expression, three ERFs had high expression levels in the paradormancy and endodormancy stages, and another 9 ERFs showed high expression levels in the endodormancy and ecodormancy stages. Therefore, ERFs might play important roles at the dormancy initiation and deep dormancy stages. APETALA2-ethylene-responsive element binding protein (AP2-EREBP) was identified as a differentially expressed transcription factor among active and dormant tea shoots (Paul A. et al., 2014). In the poplar study, five of six differentially expressed ERFs had high expression levels in endodormancy; in addition, gene sets associated with EIN2 and EIN4 were expressed at high levels during endodormancy (Howe et al., 2015). The abovementioned expression analysis confirmed the hypothesis about the importance of the ethylene signaling pathway in previous dormancy studies (Rohde and Bhalerao, 2007; Ruttink et al., 2007).

Light signal transduction- and circadian clock-associated gene sets and transcription factors were significantly enriched during dormancy transition. The photoreceptor-associated gene sets of ZTL and PHYB were mainly up-regulated in the comparison of Para vs. Endo. ZTL family proteins together with cryptochromes and phototropins are well-known receptors for blue light and phytochromes, particularly PHYA and PHYB, for red and far-red light (Kami et al., 2010). PIFs are bHLH family transcription factors and can directly interact with PHYs (Duek and Fankhauser, 2005; Leivar and Quail, 2011). The transcription of PIF4/PIF5, both negative regulators of PHYB signaling, is regulated by circadian clock-mediated coincidence mechanisms, while PIF3 is not (Huq and Quail, 2002; Nomoto et al., 2012; Soy et al., 2012). The gene sets of PIF4 and PIL6 were significantly down-regulated during the transition from paradormancy to endodormancy in this study. This coincides with the low expression levels of bHLH family genes at the endodormancy and ecodormancy stages in expression profiling analysis (**Figure 4**). PIF4 also functions in plant responses to environmental cues, increasing auxin levels to the point necessary to promote elongation growth at the proper temperature (Nomoto et al., 2012). PIF4 is even involved downstream of GA signaling and directly interacts with BZR1, a brassinosteroid-activated transcription factor (de Lucas et al., 2008; Oh et al., 2012).

The gene sets related to GI, CCA1, LHY, ELF3, and ZTL were enriched among the dormancy transitions in this study. These gene sets are major components of the circadian clock, a network of transcription-translation negative feedback loops (Horvath, 2009; Cooke et al., 2012). GI-, ZTL- and LHY-related gene sets were up-regulated when buds went into para- and endodormancy. In leafy spurge, several circadian regulators, such as *ELF4, GI, FLAVIN-BINDING, KELCHREPEAT, F BOX 1* (*FKF1*) and *CAA1*, were significantly up-regulated during endodormancy and ecodormancy. In poplar, repression of *LHY* delayed bud set was observed; however, the expression of the clock genes *LHY1/2, TOC1*, and *PRR5* varied from bud dormancy formation to release (Ibáñez et al., 2010). The function of the CO/FT module in flowering regulation has been well described (Pin and Nilsson, 2012). Regulation of CO expression and protein stability by light signaling and circadian clock is a critical mechanism in *Arabidopsis* flowering regulation (Golembeski and Imaizumi, 2015; Hajdu et al., 2015; Lazaro et al., 2015). However, the recent functional discovery of *FT* in seasonal growth regulation indicated the multiple roles of the CO/FT module in regulating both flowering and dormancy in perennial plants (Bohlenius et al., 2006; Horvath, 2009; Hsu et al., 2011). CO can interact directly with DELLA protein and histone mark readers MRG1/2 during *FT* regulation (Bu et al., 2014; Xu et al., 2016). When the ecodormant tea buds sprouted, the gene set ‘binding partners of CO’ was significantly up-regulated; this indicated that the CO/FT module may play important roles in seasonal growth regulation in tea plant as well.

MADS-box genes are possible downstream components of CO/FT signaling that are closely related to bud dormancy regulation (Cooke et al., 2012). *Dormancy-associated MADS-box* (*DAM*) genes, which are MIKC^C^-type MADS-box genes and homologs to *SVP* and *AGL24* in *Arabidopsis*, were identified in multiple species (Bielenberg et al., 2008; Horvath et al., 2010; Ubi et al., 2010). In leafy spurge, the expression levels of *DAM* genes were up-regulated during dormancy formation, especially at the endodormancy stage (Horvath et al., 2010). In peach and Japanese apricot trees, the multiple *DAM* genes showed different seasonal and photoperiodic expression patterns (Sasaki et al., 2011; Yamane et al., 2011). In an extensive expression profiling analysis of poplar, several identified DAM-like genes surprisingly showed lower expression levels during endodormancy (Howe et al., 2015). In our study, several MADS-box genes showed significantly differential expression under the measured conditions (**Figure 4**). Except for one MADS-box gene (comp27798_c0_seq2) that showed very high expression at ecodormancy, all other MADS-box genes only had high expression in paradormancy, suggesting possible regulatory roles more in line with similar genes from poplar. Further studies should be performed to determine if these MADS-box genes had similar functions in bud dormancy to known *DAMs* in other species.

Seven MYB genes were involved in the top 60 differentially expressed transcription factors in this study, and three of them (comp100540_c0_seq1, comp76266_c0_seq2, comp62057_c0_seq1) showed high expression levels in endodormancy and ecodormancy. Similarly, *MYB62* and *MYB4* showed differential expression during short-day induction or dormancy transition in poplar studies (Ruttink et al., 2007; Howe et al., 2015). Functional identification of *MYB* genes in *Arabidopsis* indicated that *MYB* genes control many aspects of plant secondary metabolism, as well as the identity and fate of plant cells (Stracke et al., 2001). However, functional study of the roles of *MYB* genes in bud dormancy is required.

### Regulation of Cellular Communication during Dormancy Transition

In plants, FT, auxin and sugars are typical moveable molecules associated with activity-dormancy transitions (Cooke et al., 2012). Plasmodesmata are important channels for cell-to-cell signaling within the apex (van der Schoot and Rinne, 2011). The dynamic accumulation and hydrolyzation of callose on the plasmodesmata are similar to a switch for cellular communication that is closely associated with the states of bud dormancy (Rinne et al., 2001). 1,3-Beta-glucan synthases and 1,3-beta-glucanases are the key enzymes involved in the abovementioned processes (Rinne et al., 2011; van der Schoot and Rinne, 2011). In this study, the GO term ‘plasmodesma’ was up-regulated in paradormant and endodormant buds and down-regulated during the bud flush period. Moreover, the gene set ‘neighbors of CAL’ was up-regulated in the paradormancy and bud flush periods and down-regulated in the endodormancy and ecodormancy periods. More attention has been paid to the β-1,3-glucanase gene family in recent studies. In *Arabidopsis*, at least fifty β-1,3-glucanase genes mainly grouped into α, β and γ clades were identified, all of which contain an N-terminal signal peptide and a glycosyl hydrolase family 17 domain (Doxey et al., 2007). Approximately one hundred β-1,3-glucanase genes were identified genome-wide in poplar (Rinne et al., 2016). However, only the members of the α-clade in *Arabidopsis* and the α-clade and γ-clade members in hybrid aspen can localize to the plasmodesma (Knox and Benitez-Alfonso, 2014; Paul L. K. et al., 2014). In addition, their expression patterns vary in response to chilling and short days (Rinne et al., 2011). Therefore, it is crucial to analyze both the expression data and phylogenetic information when we consider the function of a β-1,3-glucanase gene. GA is the most important signaling hormone in β-1,3-glucanase induction during dormancy release; it acts by promoting the hydrolyzation of callose at sieve plates and plasmodesmata (Rinne et al., 2011, 2016). In tea shoots, the level of active GA is extremely low in the early and deep dormancy periods and dramatically increases prior to dormancy release (Nagar and Kumar, 2000). Our current observations also show that the overwintering buds of tea plant have fairly low substance exchange with adjacent organs in deep dormancy periods (the result is in press). The abovementioned evidence indicates that plasmodesma-mediated cellular communication is an important regulation mechanism in the dormancy transition of tea plant. In addition, the regulation of cell wall modification should be considered. The GO terms and gene sets related to the cellulose synthesis pathway were differentially expressed during dormancy transition. *Cellulase 2* and *polygalacturonase* were also identified as DEGs involved in cell wall modification in dormant tea shoots in a previous study. Although the two glucan polymers cellulose and callose are synthesized at the plasma membrane by cellulose or callose synthase complexes, respectively, much cross-talk occurs between the two processes (Schneider et al., 2016). Additionally, cell wall modification can affect the structure of plasmodesmata, causing rapid change in intercellular communication in response to environmental signals (Knox and Benitez-Alfonso, 2014).

### Major Phytohormones Involved in Dormancy Regulation

Phytohormones are a group of naturally occurring, organic substances that influence multiple physiological processes, mainly growth, differentiation and development, at low concentrations. The important roles of phytohormones in bud dormancy regulation have been frequently reviewed (Horvath et al., 2003; Cooke et al., 2012; Singh et al., 2017). GA and ABA attract substantial attention in early dormancy studies, and their functions in the dormancy mechanism are better understood. An extensive analysis of metabolite and gene expression dynamics was conducted following the timetable of dormancy initiation in poplar (Ruttink et al., 2007). GA signaling repression-related modulators were dramatically up-regulated immediately after the onset of dormancy induction. GA signaling, light signal transduction, and chromatin remodeling were recognized as major early actions stimulated by dormancy induction. The decrease in active GA levels in the early stage of dormancy formation is closely related to growth cessation (Olsen, 2010; Hoffman, 2011). As discussed above, GA is also an important regulator during dormancy release by directly inducing β-1,3-glucanases, playing key roles in the hydrolyzation of callose around plasmodesmata (Rinne et al., 2011). Flowering uses a similar strategy; the induction of GA by decapitation is also important in regrowth regulation of paradormant axillary buds in hybrid aspen (Rinne et al., 2016) and leafy spurge (Chao et al., 2006). ABA is another important hormone induced by short days in poplar following GA signaling (Ruttink et al., 2007). More than 60 genes involved in ABA signal transduction have been studied, and approximately 146 genes have been classified as ectopic ABI3 targets in poplar. In this study, signaling pathway, gene set and DEG analyses generally showed that the GA signal was repressed in the endodormancy and ecodormancy stages while the ABA signal was stimulated. Interestingly, unlike ABI3 in poplar, the gene set ‘expression targets of ABI4’ was significantly enriched in tea plant. Recently, the roles of GA and ABA signaling in poplar endodormancy maintenance were highlighted by Howe et al. (2015). This evidence indicates that GA and ABA are important hormones involved in bud dormancy initiation and maintenance.

Ethylene biosynthesis and signal transduction are triggered between GA and ABA signal peaks during dormancy induction in poplar (Ruttink et al., 2007). Furthermore, ethylene-associated gene set is up-regulated during endodormancy in poplar (Howe et al., 2015). However, the ethylene-associated gene sets were down-regulated during the transitions to endodormancy and ecodormancy in our study. A previous RNA-seq study also showed that transcriptome factor ERF2 was significantly down-regulated in dormant shoots of tea plant compared to active shoots and identified ERF2 as an important DEG related to leaf senescence (Paul A. et al., 2014). The up-regulation of *ERFs* was closely related to the phenotype of precocious leaf senescence in *Arabidopsis* (Koyama et al., 2013). Unlike poplar, the mature leaves of tea plant do not fall in autumn and winter. The differences in expression patterns of ethylene-related genes between tea plant and poplar may be caused by the unique features of deciduous plants.

Brassinosteroids (BRs) and jasmonates (JAs) are also universal hormones in plants. BRs play important roles in endogenous regulation of growth and development and JAs in plant defense as well as growth, seed germination, senescence and abscission (Davies, 2010). In previous bud dormancy studies, little attention has been paid to BRs and JAs (Rohde and Bhalerao, 2007; Cooke et al., 2012; Singh et al., 2017). Recently, BR- and JA-associated DEGs were described in detail in a poplar transcriptome analysis (Howe et al., 2015). Both signaling pathways showed differential modulation during transition to endodormancy. BRs together with auxin metabolism can be affected by temperature (Olsen, 2010). JAs are inhibitory signals for cyclin-dependent kinase activity in both the G1–S-phase and the G2–M-phase transitions (Horvath et al., 2003). Based on GSEA results in this study, the BR signaling pathway was mainly down-regulated at the endodormancy and ecodormancy stages, and the JA pathway was down-regulated at paradormancy and endodormancy but stimulated at the ecodormancy and bud flush stages. This clear differential modulation related to dormancy status transition indicates the roles of BRs and JAs in bud dormancy regulation.

Auxin was the first identified plant hormone and is now well known, playing roles in multiple biological processes in plants (Mockaitis and Estelle, 2008; Vanneste and Friml, 2009). The positive roles of auxin in removing dormancy callose from phloem were validated early on by exogenous application of synthetic auxin in *Magnolia kobus* and *Vitis vinifera* (Aloni et al., 1991; Aloni and Peterson, 1997). Subsequently, putative polar auxin transport genes, including *PttLAX1*-*PttLAX3* and *PttPIN1*-*PttPIN3*, were identified from hybrid aspen, and their expression reduction related to dormancy formation was attributed to induction by environmental cues at the end of the growing season (Schrader et al., 2003). Horvath et al. (2003) drew a comprehensive pathway model for bud dormancy regulation and highlighted the interactions of auxin with other hormone signals. In this study, the enriched gene set of PIN3, CDK and CDC2 indicated the functional roles of auxin during dormancy transition. In total, 119 auxin-associated DEGs were identified during dormancy transitions; they composed the largest group of hormone-related genes (Supplementary Material 7). Many of the auxin associated DEGs with large expression differences were classified into Group A, which clearly showed lower expression levels in endodormancy and ecodormancy buds compared with paradormancy and bud-flush buds (**Figure 5**). Generally, the GO term ‘plant growth auxin signaling’ was down-regulated in Para vs. Endo. Similarly, the formation of endodormancy in poplar buds was consistent with the low expression of auxin-associated genes in multiple reports (Horvath et al., 2008; Baba et al., 2011; Howe et al., 2015). Low auxin levels during the deep dormancy stage were observed in tea and other species (Li et al., 2003; Nagar and Sood, 2006). These findings suggest that changes in auxin content and auxin-associated genes are necessary for dormancy transitions in tea plant as has been observed in other systems. In particular, a large proportion of auxin-associated genes with large expression differences were auxin early response factors, such as SAUR and its response proteins. The auxin polar transport-related gene sets were mainly down-regulated in Endo vs. Eco. These findings indicate the greater importance of auxin early response factors and auxin polar transport-related genes in dormancy regulation.

In tea plant, from paradormancy to bud flush, the size of overwintering buds gradually increased (Wang et al., 2014). It is unclear from our results if bud expansion is due to cell division, however, many cell cycle regulators are up-regulated as the buds transitioned from endodormancy to paradormancy, suggesting that there may be cell division occurring in the winter once paradormancy is released (Supplementary Material 2). CK signaling was up-regulated in both endodormant and ecodormant buds, while a couple of particular CK-related gene sets were down-regulated in Para vs. Endo and up-regulated in Endo vs. Eco. Interestingly, compared with other hormones, the CK-associated genes with large expression differences did not show clear expression tendencies. CK likely plays important roles in cell division in dormant buds. However, weak linkage has been constructed between CK and dormancy regulation (Horvath et al., 2003; Cooke et al., 2012). Obviously, dormancy relies on a complex regulation network between hormones in plants, and most physiological processes involve multiple hormones (Vanstraelen and Benková, 2012). For example, cytokinin signaling directly interacts with auxin by regulating PIN-FORMED auxin transporters, and cytokinin-auxin cross-talk may function in outgrowth of paradormant buds (Muller and Leyser, 2011; Simaskova et al., 2015). D-type cyclins and cyclin-dependent kinase involved in cell cycle regulation are regulated collectively by various hormones, including auxin, CK, BR and GA (Horvath et al., 2003). Therefore, the functions of hormones in dormancy regulation should be investigated from a broad viewpoint.

### Comparing Dormancy Regulation in Poplar and Other Deciduous Perennials to Tea Plant

Tea plant is a thermophilic evergreen woody species that only goes into dormancy when exposed to a cold winter (beyond approximately 16° north or south) (Barua, 1969). Poplar is a deciduous woody species that is mainly distributed in temperate and cold temperate zones. As in peach, plum, and pear trees, winter cold/dormancy is necessary for the normal life cycle of poplar, but it is not necessary for tea plant. Its winter dormancy feature may have developed over the course of its slow, progressive northward migration because tea plant originates in the warm southern region of China (Yu, 1986). Moreover, the dormancy of poplar can be induced by short days, and tea plant dormancy is induced by cold and short days (Barua, 1969; Resman et al., 2010; Paul and Kumar, 2011). The mechanism of bud dormancy has been well studied in poplar, and many genes involved in dormancy regulation have been identified (Ruttink et al., 2007; Anderson et al., 2010; Singh et al., 2017). Recently, an extensive transcriptome study discovered the most important dormancy-related genes in poplar following a long time scale (Howe et al., 2015). This gives us a chance to compare the major dormancy-associated genes in tea plant and poplar on a global transcriptomic level. Based on the sequence homology analysis, we annotated the transcripts of tea plant using a poplar protein database and then summarized the DEGs, focusing particularly on chromatin, hormone, QTL and transcription factor classifications in both species (Table last one). Surprisingly, the enriched genes or gene families with a large number of members were almost the same in both species. Epigenetic mechanisms are important regulators in bud dormancy regulation (Cooke et al., 2012; Singh et al., 2017). Approximately half of the chromatin-associated genes identified in poplar also show significantly different expression in tea plant. One example is the AGO4 gene, which shows clear down-regulation during endodormancy in poplar and leafy spurge and can form RNA-induced silencing complexes with short interfering RNAs to mediate DNA methylation and transcriptional gene silencing (Horvath et al., 2008; Matzke and Mosher, 2014; Howe et al., 2015). AGO4 (comp37668_c0_seq1, comp42629_c0_seq1) also showed significant down-regulation during endodormancy in this study. Regarding hormone signaling pathways, auxin-associated genes had the most members in both poplar and tea plant. The auxin-associated DEGs were comprehensively summarized, and their potential roles in dormancy were highlighted in our study and previous studies (Wang et al., 2014; Howe et al., 2015). Moreover, the DEGs located near dormancy-related QTLs had a high match rate with DEGs in tea plant. Although there is no validated dormancy-related QTL in tea plant yet, this result provides us important inspiration for tea plant dormancy research. bHLH, C2H2, ERF, HD-ZIP, MYB, and WRKY are transcription factor families with large numbers of DEGs in both poplar and tea plant. Numerous reports about these transcription factors in current and previous dormancy studies indicate their general and important roles in dormancy regulation (Ruttink et al., 2007; Paul A. et al., 2014; Wang et al., 2014; Howe et al., 2015). However, the differentially expressed MIKC^C^-type MADS-box transcription factors, including DAM-like genes in poplar, did not have matching targets in tea plant. This suggests the functional variation of *DAM*-related genes in tea plant. It is speculated that the molecular pathways involved in tea plant dormancy regulation are consistent with those of poplar to a certain extent. However, the specific functions of individual genes in dormancy regulation of tea plant require further identification. Moreover, the lack of intact transcript information from tea plant limits homology analysis with poplar; therefore, more reliable comparison is still meaningful to improve our understanding of tea plant dormancy when tea plant genome information is available.

## Conclusion

RNA-Seq analysis was performed to discover the major molecular mechanism in bud dormancy regulation of tea plant. Buds confirmed to be in the paradormancy, endodormancy, ecodormancy and bud flush stages were used for expression profiling analysis. GSEA was carried out using the DEGs among the different comparisons. Generally, chromatin-associated genes and gene sets were significantly enriched, which indicates the important roles of epigenetic regulation mechanisms in bud dormancy transition. Through transcription factor identification and expression cluster analyses, the key transcription factors involved in bud dormancy regulation were highlighted, consisting chiefly of the phytohormone-associated pathways, light signal- and circadian clock-associated pathways, and MADS-box related gene family. We further noted that callose-related cellular communication regulation showed an important function in bud dormancy maintenance and release. Moreover, dynamic changes in the auxin signal pathways together with other hormone signaling showed strong association with bud dormancy transition. Based on sequence homology analysis, first compared and summarized the key transcription factors, chromatin-related genes, and dormancy-related QTLs. These results will provide important information for the study of bud dormancy and a better understanding of the bud dormancy mechanism of tea plant.

## Author Contributions

XH, XW, and DH conceived and designed the experiments. XH, YY, CY, and LW performed the experiments and analyzed the data. XH wrote the paper. DH and XW together with other authors revised and approved the final manuscript.

## Conflict of Interest Statement

The authors declare that the research was conducted in the absence of any commercial or financial relationships that could be construed as a potential conflict of interest.

## Footnotes

1 https://github.com/najoshi/sickle

2 https://github.com/trinityrnaseq/trinityrnaseq

3 http://www.cyverse.org/discovery-environment

4 ftp://ftp.ncbi.nlm.nih.gov/blast/db/

5 https://phytozome.jgi.doe.gov/

6 http://planttfdb.cbi.pku.edu.cn/
