# Supplementary material for: Comprehensive Transcriptome Analyses Reveal Differential Gene Expression Profiles of Camellia sinensis Axillary Buds at Para-, Endo-, Ecodormancy, and Bud Flush Stages
Source: Front Plant Sci. 2017 Apr 18;8:553. doi: 10.3389/fpls.2017.00553 (PMC5394108; doi:10.3389/fpls.2017.00553)
Supplement: Supplementary file 1 [file Data_Sheet_1.DOCX]

S1. Temperature and sunshine duration of each day during the sample collection periods (error bar shows the minimum and maximum daily temperature)


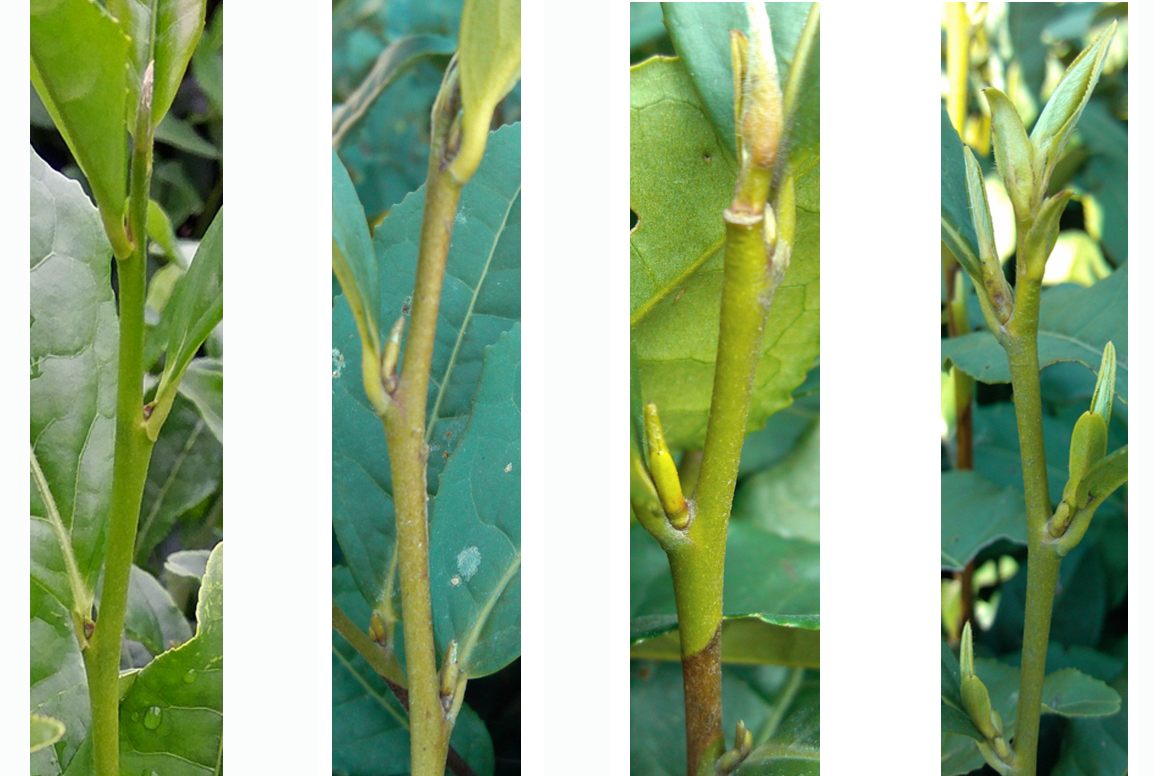


February 14th 4th

March 14th

December 1st

June 4th

S2. Appearances of shoot and axillary buds at sample collection points.





S3. Regrowth identification by moving the tea plants to green house after light pruning at partial sample collection points and the eve of bud flush (March 7th). ** stands for highly significant difference (P>0.01) between the time points of December 1^st^ and February 14^th^ by *t-test*.
