# Supplementary material for: Comprehensive Transcriptome Analyses Reveal Differential Gene Expression Profiles of Camellia sinensis Axillary Buds at Para-, Endo-, Ecodormancy, and Bud Flush Stages
Source: Front Plant Sci. 2017 Apr 18;8:553. doi: 10.3389/fpls.2017.00553 (PMC5394108; doi:10.3389/fpls.2017.00553)
Supplement: Supplementary file 3 [file Data_Sheet_3.DOCX]

S1 Primer information used in qRT-PCR detection

| **Detected transcripts** | **Forward/Reverse** | **Primer sequence (5' to 3')** |
| --- | --- | --- |
| comp28371_c0_seq1 | Forward | ATCGGCACATTGAACCCGTC |
|  | Reverse | TCCGCGAGTGCCAAGCTTAT |
| comp35019_c0_seq1 | Forward | CAGGAATGCAGCCTGAGCAT |
|  | Reverse | GCCATGGACCCTCACCTTCT |
| comp48402_c0_seq1 | Forward | TTCTCGTCGGCGAACTTTGA |
|  | Reverse | TCATCGCGTGATTCCTGGAT |
| comp58542_c0_seq1 | Forward | TCTCCCCACCCAAAGTCAGA |
|  | Reverse | ATGCGCTTACCGATGCCTAC |
| comp58768_c0_seq1 | Forward | CAGCGGCAAAGAACAGACAG |
|  | Reverse | TGGTGAAGCCCCTTAAATGG |
| comp72607_c0_seq1 | Forward | CCCTAAACATCCCCATGACC |
|  | Reverse | GCCATGGACGTTGTTTATGC |
| comp77148_c0_seq1 | Forward | GCCTCCATTGAACCAGCACA |
|  | Reverse | TACGATCGCTGATTGCGGTC |
| comp85224_c0_seq1 | Forward | CACGAAAGAAAGCATGAAGC |
|  | Reverse | ATCGTCAAAACACCTCTCCC |
| comp86513_c0_seq3 | Forward | AACCACCCCGGCTTAAGATT |
|  | Reverse | CATGCTGTTAGCATGTGCCA |
| comp89049_c0_seq1 | Forward | CCGTCCCGGAGTCAAAAATT |
|  | Reverse | TGTTCGGCATTAGAGTCGGC |
| comp89186_c0_seq5 | Forward | TCATGTTAGCAAACGGCCCC |
|  | Reverse | CAGCCAACTGCACAGACCCA |
| comp90059_c0_seq4 | Forward | CACAAGCGCTGGACATGTTG |
|  | Reverse | TGCCATTTTGGACCAAGTGG |
| comp96631_c1_seq1 | Forward | TCTCAATGCACCTTGGAGCA |
|  | Reverse | GGTATTAATCCCGAAGCCGC |
| comp96647_c1_seq3 | Forward | AAGTGTCTTGCCAAAAGCCA |
|  | Reverse | GCAACGATGTCGGACAATCT |
| comp98237_c2_seq28 | Forward | GGACCCATATCCCCAGAACA |
|  | Reverse | ACGGAAGGACTTTGGCTGAC |
| PTB | Forward | TGACCAAGCACACTCCACACTATCG |
|  | Reverse | TGCCCCCTTATCATCATCCACAA |

S2 Expression detection of selected transcripts by RNA-Seq and qRT-PCR.
