# Supplementary material for: Comprehensive Transcriptome Analyses Reveal Differential Gene Expression Profiles of Camellia sinensis Axillary Buds at Para-, Endo-, Ecodormancy, and Bud Flush Stages
Source: Front Plant Sci. 2017 Apr 18;8:553. doi: 10.3389/fpls.2017.00553 (PMC5394108; doi:10.3389/fpls.2017.00553)
Supplement: Supplementary file 4 [file Data_Sheet_4.docx]

**S1. Gene ontology (GO) enrichment analysis of differentially expressed genes between paradormancy and endodormancy.** Comparing with the gene expression in paradormancy buds, “up-regulated” and “down-regulated” gene sets are enriched for genes that either increased or decreased in gene expression in endodormancy buds respectively. Gene sets that were significant at p-value < 0.001 are presented in table. The additional parameters for up-regulated and down-regulated gene sets are marked by “-U” and “-D” tails respectively. BP, CC and MF stand for biological process, cellular component and molecular function.

| **Up-regulated (U) GO terms** | **# of Measured Entities-U** | **p-value-U** | **Hit type-U** |  | **Down-regulated (D) GO terms** | **# of Measured Entities-D** | **p-value-D** | **Hit type-D** |
| --- | --- | --- | --- | --- | --- | --- | --- | --- |
| transcription, DNA-dependent | 314 | 4.77E-11 | BP |  | cell proliferation | 102 | 4.59E-16 | BP |
| regulation of transcription, DNA-dependent | 408 | 3.20E-09 | BP |  | DNA replication initiation | 49 | 1.27E-15 | BP |
| response to water deprivation | 119 | 3.38E-09 | BP |  | regulation of DNA replication | 72 | 5.98E-14 | BP |
| ethylene mediated signaling pathway | 68 | 8.96E-08 | BP |  | DNA replication | 81 | 1.51E-13 | BP |
| response to jasmonic acid stimulus | 68 | 1.08E-07 | BP |  | histone H3-K9 methylation | 104 | 1.21E-11 | BP |
| response to chitin | 132 | 1.60E-07 | BP |  | DNA-dependent DNA replication | 72 | 3.24E-11 | BP |
| hyperosmotic salinity response | 61 | 2.50E-07 | BP |  | regulation of cell cycle | 77 | 1.61E-10 | BP |
| nitrate transport | 52 | 5.71E-07 | BP |  | DNA methylation | 104 | 1.37E-07 | BP |
| transition metal ion transport | 20 | 5.96E-07 | BP |  | regulation of G2-M transition of mitotic cell cycle | 36 | 1.99E-07 | BP |
| response to karrikin | 47 | 6.75E-07 | BP |  | cytokinesis by cell plate formation | 106 | 4.74E-07 | BP |
| response to wounding | 117 | 2.72E-06 | BP |  | regulation of meristem growth | 72 | 6.26E-07 | BP |
| response to abscisic acid stimulus | 155 | 3.56E-06 | BP |  | plant-type cell wall loosening | 8 | 4.81E-06 | BP |
| response to nitrate | 48 | 7.71E-06 | BP |  | spindle assembly | 26 | 8.15E-06 | BP |
| abscisic acid mediated signaling pathway | 97 | 1.98E-05 | BP |  | histone phosphorylation | 41 | 9.17E-06 | BP |
| circadian rhythm | 59 | 2.85E-05 | BP |  | response to brassinosteroid stimulus | 27 | 9.93E-06 | BP |
| intracellular signal transduction | 70 | 2.88E-05 | BP |  | pectin catabolic process | 16 | 1.04E-05 | BP |
| defense response | 108 | 3.24E-05 | BP |  | gene silencing | 39 | 1.33E-05 | BP |
| response to auxin stimulus | 78 | 3.95E-05 | BP |  | negative regulation of catalytic activity | 20 | 3.67E-05 | BP |
| response to cold | 149 | 7.24E-05 | BP |  | response to gibberellin stimulus | 28 | 4.77E-05 | BP |
| response to brassinosteroid stimulus | 14 | 8.05E-05 | BP |  | gibberellic acid mediated signaling pathway | 24 | 5.31E-05 | BP |
| nitrate assimilation | 13 | 9.86E-05 | BP |  | anthocyanin-containing compound biosynthetic process | 15 | 5.49E-05 | BP |
| response to stress | 44 | 0.000114 | BP |  | regulation of cell proliferation | 17 | 6.78E-05 | BP |
| response to insect | 7 | 0.000139 | BP |  | flavonoid biosynthetic process | 29 | 7.14E-05 | BP |
| anthocyanin-containing compound biosynthetic process | 13 | 0.00018 | BP |  | histone lysine methylation | 60 | 7.59E-05 | BP |
| carbohydrate metabolic process | 91 | 0.000206 | BP |  | response to karrikin | 42 | 0.000114 | BP |
| defense response to fungus | 102 | 0.000254 | BP |  | lignin catabolic process | 8 | 0.000121 | BP |
| response to ethylene stimulus | 79 | 0.000311 | BP |  | transmembrane receptor protein tyrosine kinase signaling pathway | 61 | 0.000126 | BP |
| response to mechanical stimulus | 19 | 0.000488 | BP |  | response to cyclopentenone | 42 | 0.00013 | BP |
| long-day photoperiodism, flowering | 8 | 0.000591 | BP |  | defense response | 105 | 0.000173 | BP |
| cellular response to iron ion starvation | 20 | 0.000782 | BP |  | syncytium formation | 12 | 0.00018 | BP |
| transmembrane receptor protein tyrosine kinase signaling pathway | 30 | 0.00082 | BP |  | DNA unwinding involved in DNA replication | 5 | 0.0002 | BP |
| extracellular region | 392 | 5.10E-08 | CC |  | stamen development | 28 | 0.000219 | BP |
| cell wall | 134 | 4.43E-07 | CC |  | meristem development | 16 | 0.000334 | BP |
| cytosolic ribosome | 72 | 2.53E-05 | CC |  | microtubule-based movement | 21 | 0.00036 | BP |
| plasma membrane | 961 | 8.53E-05 | CC |  | petal formation | 40 | 0.000406 | BP |
| plasmodesma | 289 | 0.000202 | CC |  | sepal formation | 40 | 0.000406 | BP |
| cytosolic small ribosomal subunit | 31 | 0.000309 | CC |  | response to light stimulus | 61 | 0.000417 | BP |
| pollen tube | 8 | 0.000764 | CC |  | response to sucrose stimulus | 78 | 0.000435 | BP |
| sequence-specific DNA binding transcription factor activity | 348 | 3.40E-17 | MF |  | response to UV-B | 38 | 0.000438 | BP |
| DNA binding | 386 | 1.63E-06 | MF |  | pattern specification process | 32 | 0.000444 | BP |
| sequence-specific DNA binding | 86 | 4.93E-05 | MF |  | iron ion transport | 16 | 0.000491 | BP |
| oxidoreductase activity, acting on paired donors, with oxidation of a pair of donors resulting in the reduction of molecular oxygen to two molecules of water | 6 | 0.000241 | MF |  | unidimensional cell growth | 34 | 0.000494 | BP |
| phosphorelay response regulator activity | 13 | 0.000363 | MF |  | nuclear division | 23 | 0.00051 | BP |
| hydrolase activity, hydrolyzing O-glycosyl compounds | 50 | 0.000485 | MF |  | lignin biosynthetic process | 19 | 0.000651 | BP |
| nutrient reservoir activity | 9 | 0.00087 | MF |  | polarity specification of adaxial-abaxial axis | 38 | 0.000677 | BP |
| oxidoreductase activity | 88 | 0.000937 | MF |  | phenylpropanoid biosynthetic process | 5 | 0.000736 | BP |
| carbohydrate binding | 51 | 0.000988 | MF |  | chromatin silencing | 73 | 0.000739 | BP |
|  |  |  |  |  | transcription, DNA-dependent | 403 | 0.000846 | BP |
|  |  |  |  |  | lipid catabolic process | 35 | 0.000857 | BP |
|  |  |  |  |  | petal development | 9 | 0.000941 | BP |
|  |  |  |  |  | extracellular region | 451 | 9.13E-18 | CC |
|  |  |  |  |  | cell wall | 152 | 1.41E-08 | CC |
|  |  |  |  |  | apoplast | 141 | 8.90E-06 | CC |
|  |  |  |  |  | anchored to membrane | 38 | 5.98E-05 | CC |
|  |  |  |  |  | microtubule | 45 | 0.000207 | CC |
|  |  |  |  |  | integral to membrane | 807 | 0.000403 | CC |
|  |  |  |  |  | anchored to plasma membrane | 20 | 0.000801 | CC |
|  |  |  |  |  | sequence-specific DNA binding transcription factor activity | 411 | 8.00E-12 | CC |
|  |  |  |  |  | DNA binding | 542 | 1.40E-05 | CC |
|  |  |  |  |  | water channel activity | 12 | 3.40E-05 | CC |
|  |  |  |  |  | oxygen binding | 44 | 7.83E-05 | CC |
|  |  |  |  |  | protein serine-threonine kinase activity | 262 | 7.97E-05 | CC |
|  |  |  |  |  | microtubule motor activity | 34 | 8.83E-05 | CC |
|  |  |  |  |  | calmodulin binding | 86 | 8.86E-05 | CC |
|  |  |  |  |  | sequence-specific DNA binding | 101 | 0.000126 | CC |
|  |  |  |  |  | heme binding | 74 | 0.000134 | CC |
|  |  |  |  |  | pectate lyase activity | 8 | 0.000163 | CC |
|  |  |  |  |  | electron carrier activity | 103 | 0.000244 | CC |
|  |  |  |  |  | UDP-glycosyltransferase activity | 27 | 0.00037 | CC |
|  |  |  |  |  | hydroquinone:oxygen oxidoreductase activity | 7 | 0.000397 | CC |

**S2. Gene ontology (GO) enrichment analysis of differentially expressed genes between endodormancy and ecodormancy.** Comparing with the gene expression in endodormancy buds, “up-regulated” and “down-regulated” gene sets are enriched for genes that either increased or decreased in gene expression in ecodormancy buds respectively. Gene sets that were significant at p-value < 0.001 are presented in table. The additional parameters for up-regulated and down-regulated gene sets are marked by “-U” and “-D” tails respectively. BP, CC and MF stand for biological process, cellular component and molecular function.

| **Up-regulated (U) GO terms** | **# of Measured Entities-U** | **p-value-U** | **Hit type-U** |  | **Down-regulated (D) GO terms** | **# of Measured Entities-D** | **p-value-D** | **Hit type-D** |
| --- | --- | --- | --- | --- | --- | --- | --- | --- |
| response to nitrate | 36 | 2.31E-08 | BP |  | plant-type cell wall biogenesis | 52 | 1.60E-08 | BP |
| nitrate transport | 39 | 2.8E-08 | BP |  | xylan biosynthetic process | 76 | 4.91E-08 | BP |
| response to salt stress | 203 | 1.06E-07 | BP |  | glucuronoxylan metabolic process | 76 | 4.91E-08 | BP |
| response to cadmium ion | 155 | 1.33E-06 | BP |  | response to chitin | 141 | 5.85E-08 | BP |
| cellular response to iron ion starvation | 18 | 5.43E-06 | BP |  | polysaccharide biosynthetic process | 43 | 7.13E-08 | BP |
| iron ion transport | 18 | 2.26E-05 | BP |  | cell tip growth | 38 | 8.79E-08 | BP |
| response to abscisic acid stimulus | 138 | 0.000138 | BP |  | negative regulation of catalytic activity | 25 | 6.20E-07 | BP |
| oxidation-reduction process | 138 | 0.00033 | BP |  | anthocyanin accumulation in tissues in response to UV light | 52 | 6.48E-07 | BP |
| hydrogen peroxide catabolic process | 34 | 0.00036 | BP |  | regulation of hormone levels | 34 | 7.10E-07 | BP |
| calcium ion transport | 36 | 0.000365 | BP |  | lignin catabolic process | 11 | 1.17E-06 | BP |
| water transport | 43 | 0.000424 | BP |  | response to wounding | 144 | 1.18E-06 | BP |
| glycolysis | 73 | 0.000464 | BP |  | cell proliferation | 79 | 1.54E-06 | BP |
| cell wall macromolecule metabolic process | 18 | 0.000558 | BP |  | cell wall biogenesis | 39 | 2.35E-06 | BP |
| oligopeptide transport | 28 | 0.000576 | BP |  | defense response | 129 | 2.39E-06 | BP |
| response to zinc ion | 16 | 0.000584 | BP |  | response to nitrate | 50 | 3.21E-06 | BP |
| extracellular region | 263 | 1.43E-06 | CC |  | secondary cell wall biogenesis | 21 | 5.22E-06 | BP |
| apoplast | 89 | 2.49E-05 | CC |  | ethylene biosynthetic process | 40 | 5.49E-06 | BP |
| cell wall | 101 | 0.00093 | CC |  | pectin catabolic process | 21 | 9.86E-06 | BP |
| sequence-specific DNA binding transcription factor activity | 368 | 1.37E-07 | MF |  | response to auxin stimulus | 106 | 1.35E-05 | BP |
| heme binding | 61 | 1.72E-07 | MF |  | response to water deprivation | 127 | 1.40E-05 | BP |
| monooxygenase activity | 36 | 2.13E-06 | MF |  | nitrate transport | 55 | 3.64E-05 | BP |
| oxygen binding | 35 | 2.18E-06 | MF |  | pattern specification process | 30 | 3.64E-05 | BP |
| iron ion binding | 55 | 3.61E-05 | MF |  | regulation of meristem growth | 85 | 3.76E-05 | BP |
| cellulose synthase activity | 7 | 0.000229 | MF |  | cytokinesis by cell plate formation | 95 | 4.61E-05 | BP |
| UDP-glycosyltransferase activity | 20 | 0.000371 | MF |  | defense response to fungus | 107 | 4.77E-05 | BP |
| amino acid transmembrane transporter activity | 19 | 0.000469 | MF |  | root morphogenesis | 26 | 5.56E-05 | BP |
| electron carrier activity | 88 | 0.000562 | MF |  | multidimensional cell growth | 48 | 5.59E-05 | BP |
| cellulose synthase (UDP-forming) activity | 6 | 0.00072 | MF |  | response to jasmonic acid stimulus | 83 | 7.37E-05 | BP |
|  |  |  |  |  | auxin polar transport | 44 | 9.90E-05 | BP |
|  |  |  |  |  | growth | 35 | 9.97E-05 | BP |
|  |  |  |  |  | lignin biosynthetic process | 25 | 0.0001 | BP |
|  |  |  |  |  | ethylene mediated signaling pathway | 76 | 0.000107 | BP |
|  |  |  |  |  | carbohydrate biosynthetic process | 24 | 0.000146 | BP |
|  |  |  |  |  | regulation of cell size | 30 | 0.000149 | BP |
|  |  |  |  |  | microtubule cytoskeleton organization | 75 | 0.000157 | BP |
|  |  |  |  |  | lipid catabolic process | 45 | 0.000275 | BP |
|  |  |  |  |  | histone H3-K9 methylation | 86 | 0.000281 | BP |
|  |  |  |  |  | transmembrane receptor protein tyrosine kinase signaling pathway | 65 | 0.000289 | BP |
|  |  |  |  |  | intracellular signal transduction | 67 | 0.000332 | BP |
|  |  |  |  |  | abscisic acid mediated signaling pathway | 94 | 0.000359 | BP |
|  |  |  |  |  | pectin biosynthetic process | 13 | 0.000374 | BP |
|  |  |  |  |  | cell wall macromolecule catabolic process | 13 | 0.000446 | BP |
|  |  |  |  |  | cellulose metabolic process | 17 | 0.000449 | BP |
|  |  |  |  |  | response to cyclopentenone | 46 | 0.000475 | BP |
|  |  |  |  |  | response to bacterium | 48 | 0.000581 | BP |
|  |  |  |  |  | regulation of G2-M transition of mitotic cell cycle | 27 | 0.000748 | BP |
|  |  |  |  |  | acetyl-CoA metabolic process | 42 | 0.000781 | BP |
|  |  |  |  |  | root hair elongation | 76 | 0.000809 | BP |
|  |  |  |  |  | extracellular region | 569 | 1.32E-32 | CC |
|  |  |  |  |  | cell wall | 178 | 1.93E-10 | CC |
|  |  |  |  |  | plant-type cell wall | 113 | 2.14E-07 | CC |
|  |  |  |  |  | anchored to membrane | 57 | 1.08E-05 | CC |
|  |  |  |  |  | integral to membrane | 928 | 1.35E-05 | CC |
|  |  |  |  |  | anchored to plasma membrane | 25 | 5.19E-05 | CC |
|  |  |  |  |  | plasma membrane | 1176 | 5.42E-05 | CC |
|  |  |  |  |  | Golgi membrane | 86 | 7.28E-05 | CC |
|  |  |  |  |  | apoplast | 162 | 0.000312 | CC |
|  |  |  |  |  | cell surface | 8 | 0.002143 | CC |
|  |  |  |  |  | microtubule | 51 | 0.00518 | CC |
|  |  |  |  |  | proteinaceous extracellular matrix | 8 | 0.011606 | CC |
|  |  |  |  |  | Golgi cisterna membrane | 14 | 0.012569 | CC |
|  |  |  |  |  | plant-type vacuole | 20 | 0.015181 | CC |
|  |  |  |  |  | Golgi apparatus | 472 | 0.031761 | CC |
|  |  |  |  |  | plasma membrane part | 17 | 0.04282 | CC |
|  |  |  |  |  | cellulose synthase activity | 17 | 2.02E-06 | MF |
|  |  |  |  |  | hydrolase activity, hydrolyzing O-glycosyl compounds | 72 | 2.69E-06 | MF |
|  |  |  |  |  | hydroquinone:oxygen oxidoreductase activity | 10 | 3.99E-06 | MF |
|  |  |  |  |  | transferase activity, transferring glycosyl groups | 162 | 1.98E-05 | MF |
|  |  |  |  |  | carboxylesterase activity | 54 | 2.07E-05 | MF |
|  |  |  |  |  | cellulose synthase (UDP-forming) activity | 14 | 2.25E-05 | MF |
|  |  |  |  |  | pectinesterase activity | 21 | 2.36E-05 | MF |
|  |  |  |  |  | enzyme inhibitor activity | 19 | 3.88E-05 | MF |
|  |  |  |  |  | lipid binding | 29 | 6.32E-05 | MF |
|  |  |  |  |  | quercetin 3-O-glucosyltransferase activity | 7 | 0.000105 | MF |
|  |  |  |  |  | aspartyl esterase activity | 16 | 0.00015 | MF |
|  |  |  |  |  | heme binding | 74 | 0.000188 | MF |
|  |  |  |  |  | sequence-specific DNA binding transcription factor activity | 411 | 0.000261 | MF |
|  |  |  |  |  | protein kinase activity | 114 | 0.000309 | MF |
|  |  |  |  |  | transferase activity, transferring acyl groups other than amino-acyl groups | 14 | 0.000402 | MF |
|  |  |  |  |  | UDP-glucosyltransferase activity | 12 | 0.000472 | MF |
|  |  |  |  |  | UDP-glycosyltransferase activity | 28 | 0.000511 | MF |
|  |  |  |  |  | serine-type carboxypeptidase activity | 17 | 0.000524 | MF |
|  |  |  |  |  | protein serine-threonine kinase activity | 288 | 0.00062 | MF |
|  |  |  |  |  | transferase activity, transferring hexosyl groups | 46 | 0.000691 | MF |
|  |  |  |  |  | nutrient reservoir activity | 17 | 0.00091 | MF |

**S3. Gene ontology (GO) enrichment analysis of differentially expressed genes between ecodormancy and bud flush.** Comparing with the gene expression in ecodormancy buds, “up-regulated” and “down-regulated” gene sets are enriched for genes that either increased or decreased in gene expression in buds at bud flush phase respectively. Gene sets that were significant at p-value < 0.001 are presented in table. The additional parameters for up-regulated and down-regulated gene sets are marked by “-U” and “-D” tails respectively. BP, CC and MF stand for biological process, cellular component and molecular function.

| **Up-regulated (U) GO terms** | **# of Measured Entities-U** | **p-value-U** | **Hit type-U** |  | **Down-regulated (D) GO terms** | **# of Measured Entities-D** | **p-value-D** | **Hit type-D** |
| --- | --- | --- | --- | --- | --- | --- | --- | --- |
| cell proliferation | 101 | 5.5E-25 | BP |  | translation | 89 | 8.98E-24 | BP |
| DNA replication initiation | 51 | 5.22E-21 | BP |  | RNA methylation | 41 | 2.64E-15 | BP |
| histone H3-K9 methylation | 108 | 8.83E-20 | BP |  | ribosome biogenesis | 24 | 5.63E-15 | BP |
| regulation of DNA replication | 74 | 5.18E-19 | BP |  | response to cadmium ion | 149 | 8.54E-10 | BP |
| regulation of cell cycle | 74 | 6.42E-16 | BP |  | response to salt stress | 220 | 7.81E-07 | BP |
| cytokinesis by cell plate formation | 105 | 5.54E-14 | BP |  | hyperosmotic salinity response | 63 | 5.88E-06 | BP |
| DNA methylation | 103 | 8.55E-13 | BP |  | oxidation-reduction process | 150 | 9.34E-06 | BP |
| histone phosphorylation | 39 | 2.02E-12 | BP |  | response to water deprivation | 120 | 9.51E-06 | BP |
| DNA replication | 84 | 6.32E-12 | BP |  | response to nitrate | 51 | 1.16E-05 | BP |
| regulation of G2-M transition of mitotic cell cycle | 36 | 9.15E-12 | BP |  | response to gibberellin stimulus | 32 | 1.18E-05 | BP |
| spindle assembly | 25 | 2.87E-10 | BP |  | response to cold | 131 | 1.22E-05 | BP |
| DNA-dependent DNA replication | 70 | 2.2E-09 | BP |  | cell wall modification | 26 | 1.36E-05 | BP |
| microtubule cytoskeleton organization | 82 | 2.49E-09 | BP |  | gluconeogenesis | 47 | 3.23E-05 | BP |
| lipid catabolic process | 39 | 4.43E-09 | BP |  | plant-type cell wall organization | 30 | 3.86E-05 | BP |
| regulation of meristem growth | 84 | 1.27E-08 | BP |  | translational elongation | 6 | 9.61E-05 | BP |
| plant-type cell wall organization | 60 | 1.78E-08 | BP |  | nitrate transport | 57 | 0.000104 | BP |
| nuclear division | 22 | 6.91E-08 | BP |  | glycolysis | 65 | 0.000172 | BP |
| transmembrane receptor protein tyrosine kinase signaling pathway | 63 | 3.31E-07 | BP |  | amino acid transport | 50 | 0.000177 | BP |
| microtubule-based movement | 19 | 3.87E-07 | BP |  | response to chitin | 139 | 0.000274 | BP |
| gene silencing | 42 | 6.57E-07 | BP |  | defense response to fungus | 103 | 0.000281 | BP |
| histone lysine methylation | 69 | 6.72E-07 | BP |  | aerobic respiration | 8 | 0.000314 | BP |
| chromatin silencing | 75 | 6.85E-07 | BP |  | response to abscisic acid stimulus | 167 | 0.000421 | BP |
| regulation of cell size | 31 | 7.51E-07 | BP |  | response to karrikin | 42 | 0.000534 | BP |
| petal formation | 41 | 2.46E-06 | BP |  | nitrate assimilation | 18 | 0.000605 | BP |
| sepal formation | 41 | 2.46E-06 | BP |  | abscisic acid mediated signaling pathway | 97 | 0.000779 | BP |
| regulation of cell proliferation | 19 | 4.72E-06 | BP |  | anthocyanin-containing compound biosynthetic process | 10 | 0.000799 | BP |
| growth | 35 | 7.71E-06 | BP |  | response to sucrose stimulus | 58 | 0.000863 | BP |
| translation | 151 | 1.11E-05 | BP |  | cytosolic ribosome | 66 | 4.37E-24 | CC |
| cytokinesis | 22 | 1.89E-05 | BP |  | cytosolic large ribosomal subunit | 41 | 8.52E-21 | CC |
| root morphogenesis | 28 | 2.97E-05 | BP |  | ribosome | 54 | 8.96E-20 | CC |
| microtubule nucleation | 41 | 5.08E-05 | BP |  | cytosolic small ribosomal subunit | 28 | 1.77E-15 | CC |
| nucleolus organization | 15 | 5.56E-05 | BP |  | cell wall | 102 | 5.73E-11 | CC |
| lignin catabolic process | 10 | 9.99E-05 | BP |  | plasmodesma | 260 | 6.79E-10 | CC |
| cell wall modification | 42 | 0.000104 | BP |  | large ribosomal subunit | 11 | 2.88E-08 | CC |
| response to cyclopentenone | 40 | 0.000105 | BP |  | plasma membrane | 874 | 4.03E-07 | CC |
| chromatin silencing by small RNA | 57 | 0.000112 | BP |  | nucleolus | 87 | 7.16E-07 | CC |
| stamen development | 28 | 0.000176 | BP |  | cytoplasm | 858 | 2.91E-05 | CC |
| methylation-dependent chromatin silencing | 64 | 0.000194 | BP |  | apoplast | 102 | 9.3E-05 | CC |
| polysaccharide biosynthetic process | 47 | 0.000203 | BP |  | cytosol | 551 | 0.000192 | CC |
| acetyl-CoA metabolic process | 48 | 0.000229 | BP |  | vacuolar membrane | 157 | 0.000416 | CC |
| auxin polar transport | 45 | 0.000408 | BP |  | membrane | 399 | 0.000863 | CC |
| nucleosome assembly | 20 | 0.000428 | BP |  | structural constituent of ribosome | 80 | 3.09E-31 | MF |
| pectin catabolic process | 21 | 0.000475 | BP |  | sequence-specific DNA binding transcription factor activity | 407 | 9.3E-07 | MF |
| cellulose catabolic process | 11 | 0.000483 | BP |  | oxidoreductase activity | 88 | 1.44E-06 | MF |
| asymmetric cell division | 18 | 0.000499 | BP |  | oxygen binding | 39 | 8.95E-06 | MF |
| negative regulation of catalytic activity | 27 | 0.000542 | BP |  | electron carrier activity | 99 | 1.88E-05 | MF |
| cellular glucan metabolic process | 8 | 0.000828 | BP |  | heme binding | 68 | 4.55E-05 | MF |
| plant-type cell wall loosening | 11 | 0.000836 | BP |  | hydrolase activity, hydrolyzing O-glycosyl compounds | 37 | 5.94E-05 | MF |
| extracellular region | 529 | 2.51E-28 | CC |  | copper ion binding | 59 | 9.35E-05 | MF |
| cell wall | 186 | 9.35E-15 | CC |  | symporter activity | 29 | 0.000158 | MF |
| microtubule | 46 | 2.48E-12 | CC |  | translation elongation factor activity | 9 | 0.000173 | MF |
| anchored to membrane | 50 | 7.58E-10 | CC |  | sequence-specific DNA binding | 103 | 0.000208 | MF |
| plant-type cell wall | 111 | 3.68E-09 | CC |  | monooxygenase activity | 42 | 0.000259 | MF |
| apoplast | 155 | 5.25E-09 | CC |  | rRNA binding | 10 | 0.000452 | MF |
| cytosolic ribosome | 72 | 3.13E-06 | CC |  | structural constituent of cytoskeleton | 8 | 0.000705 | MF |
| cytosolic large ribosomal subunit | 40 | 5.29E-06 | CC |  |  |  |  |  |
| anchored to plasma membrane | 27 | 6.4E-06 | CC |  |  |  |  |  |
| nucleosome | 17 | 3.81E-05 | CC |  |  |  |  |  |
| ribosome | 123 | 0.000123 | CC |  |  |  |  |  |
| cytosolic small ribosomal subunit | 33 | 0.000418 | CC |  |  |  |  |  |
| microtubule associated complex | 7 | 0.000728 | CC |  |  |  |  |  |
| microtubule motor activity | 33 | 1.31E-09 | MF |  |  |  |  |  |
| hydrolase activity, hydrolyzing O-glycosyl compounds | 68 | 7.99E-07 | MF |  |  |  |  |  |
| structural constituent of ribosome | 141 | 1.16E-06 | MF |  |  |  |  |  |
| carboxylesterase activity | 50 | 3.09E-06 | MF |  |  |  |  |  |
| receptor activity | 58 | 6.03E-05 | MF |  |  |  |  |  |
| cellulase activity | 10 | 7.36E-05 | MF |  |  |  |  |  |
| pectate lyase activity | 6 | 0.0001 | MF |  |  |  |  |  |
| hydroquinone:oxygen oxidoreductase activity | 9 | 0.000144 | MF |  |  |  |  |  |
| copper ion binding | 103 | 0.000192 | MF |  |  |  |  |  |
| UDP-glycosyltransferase activity | 26 | 0.000352 | MF |  |  |  |  |  |
| transmembrane transporter activity | 11 | 0.000711 | MF |  |  |  |  |  |
| xyloglucan:xyloglucosyl transferase activity | 8 | 0.000828 | MF |  |  |  |  |  |
| serine-type carboxypeptidase activity | 18 | 0.00092 | MF |  |  |  |  |  |

**S4.** **Signaling pathway enrichment analysis of differentially expressed genes between the four comparisons of paradormancy (Para) and endodormancy (Endo), endodormancy (Endo) vs. ecodormancy (Eco), and ecodormancy (Eco) vs. bud flush (Flush).** “Up- or down-regulated” gene sets are enriched for genes that changed in gene expression, without considering the direction of change of the individual genes. “Up-regulated” and “down-regulated” gene sets are enriched for genes that either increased or decreased in gene expression respectively.

| **Comparisons** | **Up- or down-regulated** | |  | **Up-regulated** | |  | **Down-regulated** | |
| --- | --- | --- | --- | --- | --- | --- | --- | --- |
|  | **Signaling pathway** | **p-value** |  | **Signaling pathway** | **p-value** |  | **Signaling pathway** | **p-value** |
| Para vs. Endo | Phytochrome Signaling | 0.000195 |  | Flagellin Signaling | 0.004664 |  | Gibberellin Signaling | 0.002784 |
|  | Cryptochrome Signaling | 0.001434 |  | Cryptochrome Signaling | 0.00786 |  | Phytochrome Signaling | 0.005852 |
|  | SCARECROW Signaling | 0.010778 |  | Seed Development ABA Signaling | 0.010056 |  | Plant Growth Auxin Signaling | 0.008652 |
|  | Cytokinins Signaling | 0.028011 |  | Phytochrome Signaling | 0.010338 |  | Cryptochrome Signaling | 0.009662 |
|  | Plant Growth Auxin Signaling | 0.028331 |  | Cold-Stress Signaling | 0.0113 |  | Phosphate Deprivation Signaling | 0.019167 |
|  | Phototropin Signaling | 0.034064 |  | Stress ABA Signaling | 0.015385 |  |  |  |
|  | Gibberellin Signaling | 0.040507 |  | Phototropin Signaling | 0.020708 |  |  |  |
|  | Seed Development ABA Signaling | 0.043701 |  | Calcium Signaling | 0.024449 |  |  |  |
|  | Calcium Signaling | 0.049175 |  | Cytokinins Signaling | 0.040613 |  |  |  |
|  |  |  |  | Salicylic Acid Signaling | 0.042502 |  |  |  |
| Endo vs. Eco | Cytokinins Signaling | 0.004821 |  | Cytokinins Signaling | 0.002292 |  | Salicylic Acid Signaling | 0.001733 |
|  | Salicylic Acid Signaling | 0.00769 |  | Plant Growth Auxin Signaling | 0.026839 |  | Phosphate Deprivation Signaling | 0.011467 |
|  | Systemin Signaling | 0.012084 |  | Stress ABA Signaling | 0.028487 |  | Jasmonic Acid, Ethylene, and Salicylic Acid Crosstalk Signaling | 0.034735 |
|  | Ethylene Signaling | 0.012326 |  | SCARECROW Signaling | 0.029523 |  |  |  |
|  | Jasmonic Acid Signaling | 0.036754 |  |  |  |  |  |  |
|  | Cold-Stress Signaling | 0.043326 |  |  |  |  |  |  |
|  | SCARECROW Signaling | 0.045963 |  |  |  |  |  |  |
|  | Phototropin Signaling | 0.047335 |  |  |  |  |  |  |
|  | Phosphate Deprivation Signaling | 0.049894 |  |  |  |  |  |  |
| Eco vs. Flush | Cryptochrome Signaling | 0.035395 |  |  |  |  | Cryptochrome Signaling | 0.006052 |
|  |  |  |  |  |  |  | Gibberellin Signaling | 0.012505 |
|  |  |  |  |  |  |  | Phosphate Deprivation Signaling | 0.046033 |
|  |  |  |  |  |  |  |  |  |

**S5. Pathway studio gene sets and subnetworks differentially expressed between paradormancy and endodormancy.** “Up- or down-regulated” gene sets are enriched for genes that changed in gene expression, without considering the direction of change of the individual genes. “Up-regulated” and “down-regulated” gene sets are enriched for genes that either increased or decreased in gene expression respectively. Gene sets and subnetworks that were significant at p-value < 0.05 are presented in table.

| **Up- or down-regulated** | |  | **Up-regulated** | |  | **Down-regulated** | |
| --- | --- | --- | --- | --- | --- | --- | --- |
| **Gene sets and subnetworks** | **p-value** |  | **Gene sets and subnetworks** | **p-value** |  | **Gene sets and subnetworks** | **p-value** |
| Expression targets of SFR6 | 0.000512 |  | Expression targets of SFR6 | 0.001606 |  | Expression targets of LFY | 0.00791 |
| Expression targets of CCA1 | 0.001543 |  | Expression targets of LHY | 0.008166 |  | Expression targets of BRI1 | 0.007916 |
| Expression targets of SPL3 | 0.00484 |  | Expression targets of ZTL | 0.017426 |  | Expression targets of SVP | 0.008556 |
| Expression targets of ZTL | 0.009216 |  | Expression targets of ABI4 | 0.0183 |  | Expression targets of PIF4 | 0.025232 |
| Expression targets of ATAN11 | 0.009551 |  | Expression targets of ELF3 | 0.025772 |  | Expression targets of PIL6 | 0.026512 |
| Expression targets of BRI1 | 0.012182 |  | miRNA targets of MIR164A | 0.045246 |  | Expression targets of EIN3 | 0.02845 |
| Expression targets of FKF1 | 0.017113 |  | Binding partners of AHP1 | 0.0013 |  | Expression targets of SHY2 | 0.032045 |
| Expression targets of LHY | 0.017464 |  | Binding partners of AHP3 | 0.003861 |  | Expression targets of CCA1 | 0.033382 |
| Expression targets of ELF3 | 0.020145 |  | Binding partners of calmodulin | 0.014386 |  | Expression targets of RPL | 0.035169 |
| Expression targets of SHY2 | 0.02019 |  | Binding partners of CHIP | 0.018293 |  | Expression targets of STM | 0.048356 |
| Expression targets of LFY | 0.025158 |  | Binding partners of MADS box protein | 0.043166 |  | miRNA targets of MIR159A | 0.023894 |
| Expression targets of SVP | 0.027485 |  | Binding partners of PHYB | 0.047207 |  | miRNA targets of MIR157D | 0.037408 |
| Expression targets of STM | 0.027747 |  | Protein modification targets of mitogen-activated protein kinase | 0.039634 |  | Binding partners of TTG1 | 0.003197 |
| Expression targets of PIF4 | 0.032093 |  | Neighbors of senescence | 0.00058 |  | Binding partners of SEU | 0.008308 |
| Expression targets of PI | 0.033536 |  | Neighbors of stomata conductance | 0.000773 |  | Binding partners of SVP | 0.010205 |
| Expression targets of PRR9 | 0.041452 |  | Neighbors of drought tolerance | 0.003244 |  | Binding partners of MADS box protein | 0.01092 |
| Expression targets of bZIP transcription factor | 0.044368 |  | Neighbors of photoperiodism, flowering | 0.012971 |  | Binding partners of CDC2 | 0.016639 |
| Expression targets of AP3 | 0.044613 |  | Neighbors of shoot development | 0.013428 |  | Binding partners of TFL2 | 0.019583 |
| miRNA targets of MIR157D | 0.030509 |  | Neighbors of organ growth | 0.014532 |  | Binding partners of heat shock protein | 0.019754 |
| miRNA targets of MIR159A | 0.041998 |  | Neighbors of imbibition | 0.015005 |  | Binding partners of HD1 | 0.022207 |
| Binding partners of SEU | 0.001073 |  | Neighbors of transmembrane potential | 0.016829 |  | Binding partners of basic-helix-loop-helix protein | 0.022573 |
| Binding partners of SVP | 0.003999 |  | Neighbors of response to dehydration | 0.020394 |  | Binding partners of thioredoxin | 0.026318 |
| Binding partners of MADS box protein | 0.005946 |  | Neighbors of phloem loading | 0.024162 |  | Binding partners of calmodulin | 0.030374 |
| Binding partners of AHP1 | 0.006491 |  | Neighbors of response to phosphate starvation | 0.024341 |  | Binding partners of KNAT1 | 0.034776 |
| Binding partners of TTG1 | 0.013611 |  | Neighbors of light response | 0.024355 |  | Binding partners of CO | 0.037746 |
| Binding partners of AHP3 | 0.015431 |  | Neighbors of somatic embryogenesis | 0.024989 |  | Binding partners of KNAT2 | 0.042162 |
| Binding partners of CDK | 0.025495 |  | Neighbors of xylem loading | 0.028035 |  | Binding partners of SGT1B | 0.04957 |
| Binding partners of calmodulin | 0.027038 |  | Neighbors of auxin metabolism | 0.031962 |  | Neighbors of petal development | 0.000181 |
| Binding partners of AHP2 | 0.027274 |  | Neighbors of fruit growth | 0.033621 |  | Neighbors of DNA replication initiation | 0.000235 |
| Binding partners of CDC2 | 0.027805 |  | Neighbors of leaf shape | 0.042593 |  | Neighbors of DNA replication | 0.000361 |
| Binding partners of lectin | 0.028397 |  | Neighbors of sugar response | 0.043892 |  | Neighbors of meristem identity | 0.001427 |
| Binding partners of leucine zipper | 0.030949 |  | Neighbors of fertilization | 0.044153 |  | Neighbors of meristem growth | 0.002299 |
| Binding partners of TFL2 | 0.031165 |  | Neighbors of acid sensitivity | 0.045852 |  | Neighbors of fruit growth | 0.003479 |
| Binding partners of CO | 0.031237 |  | Neighbors of senescence | 0.00058 |  | Neighbors of organ formation | 0.004416 |
| Binding partners of RPL | 0.032739 |  | Neighbors of stomata conductance | 0.000773 |  | Neighbors of response to heat shock | 0.004563 |
| Binding partners of Rho | 0.033286 |  | Neighbors of drought tolerance | 0.003244 |  | Neighbors of ripening | 0.005907 |
| Binding partners of CHIP | 0.038887 |  | Neighbors of photoperiodism, flowering | 0.012971 |  | Neighbors of pigment biosynthesis | 0.006535 |
| Binding partners of CBL3 | 0.049394 |  | Neighbors of shoot development | 0.013428 |  | Neighbors of epidermal cell differentiation | 0.007302 |
| Protein modification targets of MPK6 | 0.018915 |  | Neighbors of organ growth | 0.014532 |  | Neighbors of shoot regeneration | 0.007542 |
| Neighbors of ABA insensitivity | 0.044055 |  | Neighbors of imbibition | 0.015005 |  | Neighbors of flower color | 0.009402 |
| Neighbors of DNA replication initiation | 0.000256 |  | Neighbors of transmembrane potential | 0.016829 |  | Neighbors of cotyledon growth | 0.010161 |
| Neighbors of callus initiation | 0.000554 |  | Neighbors of response to dehydration | 0.020394 |  | Neighbors of shade avoidance | 0.010215 |
| Neighbors of organ growth | 0.001213 |  | Neighbors of phloem loading | 0.024162 |  | Neighbors of flower identity | 0.010605 |
| Neighbors of shoot development | 0.00134 |  | Neighbors of response to phosphate starvation | 0.024341 |  | Neighbors of proanthocyanidin metabolism | 0.011084 |
| Neighbors of fruit growth | 0.001364 |  | Neighbors of light response | 0.024355 |  | Neighbors of water transport | 0.013058 |
| Neighbors of petal development | 0.001556 |  | Neighbors of somatic embryogenesis | 0.024989 |  | Neighbors of trichome development | 0.015676 |
| Neighbors of meristem identity | 0.002156 |  | Neighbors of xylem loading | 0.028035 |  | Neighbors of cytokinesis | 0.017769 |
| Neighbors of calcium ion homeostasis | 0.002679 |  | Neighbors of auxin metabolism | 0.031962 |  | Neighbors of trichome differentiation | 0.018031 |
| Neighbors of organ formation | 0.002774 |  | Neighbors of fruit growth | 0.033621 |  | Neighbors of cytokinin content | 0.018348 |
| Neighbors of meristem size | 0.004597 |  | Neighbors of leaf shape | 0.042593 |  | Neighbors of lignin biosynthesis trait | 0.018728 |
| Neighbors of stomata conductance | 0.006181 |  | Neighbors of sugar response | 0.043892 |  | Neighbors of meristem size | 0.01924 |
| Neighbors of xylem loading | 0.008607 |  | Neighbors of fertilization | 0.044153 |  | Neighbors of primordium initiation | 0.02111 |
| Neighbors of shade avoidance | 0.008695 |  | Neighbors of acid sensitivity | 0.045852 |  | Neighbors of trichome yield | 0.02355 |
| Neighbors of flower color | 0.009065 |  |  |  |  | Neighbors of cell homeostasis | 0.024166 |
| Neighbors of auxin metabolism | 0.009693 |  |  |  |  | Neighbors of specification of petal identity | 0.024389 |
| Neighbors of shoot regeneration | 0.011084 |  |  |  |  | Neighbors of leaf initiation | 0.024896 |
| Neighbors of meristem growth | 0.011186 |  |  |  |  | Neighbors of root differentiation | 0.024995 |
| Neighbors of response to heat shock | 0.011293 |  |  |  |  | Neighbors of flower patterning | 0.025447 |
| Neighbors of carpel development | 0.012323 |  |  |  |  | Neighbors of pigmentation | 0.025509 |
| Neighbors of leaf initiation | 0.015265 |  |  |  |  | Neighbors of maintenance of DNA methylation | 0.027924 |
| Neighbors of G1/S transition checkpoint | 0.015278 |  |  |  |  | Neighbors of fruit development | 0.02798 |
| Neighbors of meristem function | 0.015623 |  |  |  |  | Neighbors of G1/S transition | 0.028902 |
| Neighbors of response to dehydration | 0.015891 |  |  |  |  | Neighbors of lignification | 0.030273 |
| Neighbors of cytokinin content | 0.016422 |  |  |  |  | Neighbors of G1 phase | 0.030383 |
| Neighbors of tuber development | 0.018199 |  |  |  |  | Neighbors of root patterning | 0.030456 |
| Neighbors of trichome development | 0.020611 |  |  |  |  | Neighbors of HRR | 0.036609 |
| Neighbors of flower patterning | 0.020755 |  |  |  |  | Neighbors of carpel development | 0.036662 |
| Neighbors of cell homeostasis | 0.02155 |  |  |  |  | Neighbors of xylem loading | 0.037457 |
| Neighbors of photoperiodism, flowering | 0.02195 |  |  |  |  | Neighbors of intracellular pH | 0.042291 |
| Neighbors of water transport | 0.023338 |  |  |  |  | Neighbors of inflorescence patterning | 0.042514 |
| Neighbors of fruit set | 0.023507 |  |  |  |  | Neighbors of calcium ion homeostasis | 0.045717 |
| Neighbors of lignification | 0.025238 |  |  |  |  | Neighbors of flower type | 0.046897 |
| Neighbors of response to phosphate starvation | 0.025739 |  |  |  |  | Neighbors of gravitropism | 0.048485 |
| Neighbors of proanthocyanidin metabolism | 0.028084 |  |  |  |  | Neighbors of H2O | 1.22E-05 |
| Neighbors of cold acclimation | 0.030731 |  |  |  |  | Neighbors of anthocyanins | 4.59E-05 |
| Neighbors of root differentiation | 0.032249 |  |  |  |  | Neighbors of CAL | 5.78E-05 |
| Neighbors of HRR | 0.032652 |  |  |  |  | Neighbors of petal development | 0.000194 |
| Neighbors of anthocyanin metabolism | 0.033289 |  |  |  |  | Neighbors of cytochrome P450 | 0.000237 |
| Neighbors of cytokinin metabolism | 0.033481 |  |  |  |  | Neighbors of Flavonol | 0.000239 |
| Neighbors of primordium initiation | 0.033817 |  |  |  |  | Neighbors of DNA replication initiation | 0.000266 |
| Neighbors of flower identity | 0.035147 |  |  |  |  | Neighbors of DNA replication | 0.000337 |
| Neighbors of nitrate uptake | 0.035853 |  |  |  |  | Neighbors of Sinapinic acid | 0.001303 |
| Neighbors of G1 phase | 0.036872 |  |  |  |  | Neighbors of meristem identity | 0.001366 |
| Neighbors of inflorescence patterning | 0.04066 |  |  |  |  | Neighbors of AGL24 | 0.001402 |
| Neighbors of cell fate determination | 0.040747 |  |  |  |  | Neighbors of flavonoids | 0.00151 |
| Neighbors of anther development | 0.042204 |  |  |  |  | Neighbors of Proanthocyanidin | 0.002186 |
| Neighbors of heat tolerance | 0.042548 |  |  |  |  | Neighbors of meristem growth | 0.002252 |
| Neighbors of intracellular pH | 0.043008 |  |  |  |  | Neighbors of aromadendrin | 0.002302 |
| Neighbors of stomata density | 0.046404 |  |  |  |  | Neighbors of PAI2 | 0.003343 |
| Neighbors of asymmetric cytokinesis | 0.049245 |  |  |  |  | Neighbors of fruit growth | 0.003478 |
| Neighbors of H2O | 2.61E-05 |  |  |  |  | Neighbors of AP3 | 0.003489 |
| Neighbors of CAL | 0.00011 |  |  |  |  | Neighbors of Taxifoliol | 0.003534 |
| Neighbors of DNA replication initiation | 0.000278 |  |  |  |  | Neighbors of CO2 | 0.00392 |
| Neighbors of KCS1 | 0.000509 |  |  |  |  | Neighbors of organ formation | 0.004295 |
| Neighbors of callus initiation | 0.000623 |  |  |  |  | Neighbors of response to heat shock | 0.004654 |
| Neighbors of anthocyanins | 0.000723 |  |  |  |  | Neighbors of DFR | 0.00474 |
| Neighbors of flavonoids | 0.000777 |  |  |  |  | Neighbors of ripening | 0.005458 |
| Neighbors of Flavonol | 0.001493 |  |  |  |  | Neighbors of NADP+ | 0.005842 |
| Neighbors of organ growth | 0.00152 |  |  |  |  | Neighbors of pigment biosynthesis | 0.006295 |
| Neighbors of fruit growth | 0.001529 |  |  |  |  | Neighbors of Cyanidin | 0.006694 |
| Neighbors of DFR | 0.001609 |  |  |  |  | Neighbors of epidermal cell differentiation | 0.006921 |
| Neighbors of shoot development | 0.00165 |  |  |  |  | Neighbors of Ca2+ | 0.006934 |
| Neighbors of petal development | 0.001768 |  |  |  |  | Neighbors of shoot regeneration | 0.007256 |
| Neighbors of CUC3 | 0.001877 |  |  |  |  | Neighbors of RBR1 | 0.007935 |
| Neighbors of aromadendrin | 0.002353 |  |  |  |  | Neighbors of PI | 0.008242 |
| Neighbors of meristem identity | 0.002556 |  |  |  |  | Neighbors of flower color | 0.009183 |
| Neighbors of monosaccharides | 0.002603 |  |  |  |  | Neighbors of TTG2 | 0.009211 |
| Neighbors of calcium ion homeostasis | 0.003015 |  |  |  |  | Neighbors of shade avoidance | 0.009775 |
| Neighbors of organ formation | 0.003218 |  |  |  |  | Neighbors of cotyledon growth | 0.009912 |
| Neighbors of phenylpropanoids | 0.003513 |  |  |  |  | Neighbors of flower identity | 0.010643 |
| Neighbors of PAI2 | 0.003721 |  |  |  |  | Neighbors of proanthocyanidin metabolism | 0.010838 |
| Neighbors of Taxifoliol | 0.003746 |  |  |  |  | Neighbors of adenylate dimethylallyltransferase | 0.011013 |
| Neighbors of steroids | 0.003896 |  |  |  |  | Neighbors of DNA methyltransferases | 0.012358 |
| Neighbors of Sinapinic acid | 0.004541 |  |  |  |  | Neighbors of Naringenin | 0.012782 |
| Neighbors of HSF1 | 0.004551 |  |  |  |  | Neighbors of water transport | 0.012969 |
| Neighbors of meristem size | 0.005227 |  |  |  |  | Neighbors of MYB0 | 0.013076 |
| Neighbors of CO2 | 0.005441 |  |  |  |  | Neighbors of AGL8 | 0.01343 |
| Neighbors of DNA methyltransferases | 0.005605 |  |  |  |  | Neighbors of trichome development | 0.015175 |
| Neighbors of succinate | 0.005645 |  |  |  |  | Neighbors of cytokinesis | 0.015976 |
| Neighbors of BGL2 | 0.006091 |  |  |  |  | Neighbors of AP1 | 0.01622 |
| Neighbors of CCR2 | 0.006367 |  |  |  |  | Neighbors of trichome differentiation | 0.01667 |
| Neighbors of Ca2+ | 0.006705 |  |  |  |  | Neighbors of meristem size | 0.016872 |
| Neighbors of Proanthocyanidin | 0.006777 |  |  |  |  | Neighbors of cytokinin content | 0.017558 |
| Neighbors of stomata conductance | 0.006794 |  |  |  |  | Neighbors of lignin biosynthesis trait | 0.018094 |
| Neighbors of suberin | 0.006885 |  |  |  |  | Neighbors of steroids | 0.018631 |
| Neighbors of Cyanidin | 0.007192 |  |  |  |  | Neighbors of AFO | 0.020315 |
| Neighbors of cytochrome P450 | 0.008286 |  |  |  |  | Neighbors of primordium initiation | 0.020926 |
| Neighbors of chitinase | 0.00842 |  |  |  |  | Neighbors of LOB | 0.021104 |
| Neighbors of RBR1 | 0.008583 |  |  |  |  | Neighbors of trichome yield | 0.022022 |
| Neighbors of AS2 | 0.008754 |  |  |  |  | Neighbors of root differentiation | 0.023056 |
| Neighbors of NADP+ | 0.00894 |  |  |  |  | Neighbors of cell homeostasis | 0.023081 |
| Neighbors of xylem loading | 0.009009 |  |  |  |  | Neighbors of leaf initiation | 0.023122 |
| Neighbors of PRR7 | 0.009359 |  |  |  |  | Neighbors of flower patterning | 0.024245 |
| Neighbors of flower color | 0.009559 |  |  |  |  | Neighbors of specification of petal identity | 0.024308 |
| Neighbors of shade avoidance | 0.009648 |  |  |  |  | Neighbors of pigmentation | 0.024731 |
| Neighbors of MET1 | 0.010833 |  |  |  |  | Neighbors of WOX5 | 0.025447 |
| Neighbors of auxin metabolism | 0.010911 |  |  |  |  | Neighbors of fruit development | 0.027292 |
| Neighbors of adenylate dimethylallyltransferase | 0.011178 |  |  |  |  | Neighbors of succinate | 0.027436 |
| Neighbors of shoot regeneration | 0.012121 |  |  |  |  | Neighbors of G1/S transition | 0.028266 |
| Neighbors of response to heat shock | 0.012142 |  |  |  |  | Neighbors of maintenance of DNA methylation | 0.028375 |
| Neighbors of meristem growth | 0.01238 |  |  |  |  | Neighbors of lignification | 0.02853 |
| Neighbors of carpel development | 0.013202 |  |  |  |  | Neighbors of G1 phase | 0.028692 |
| Neighbors of Naringenin | 0.013661 |  |  |  |  | Neighbors of root patterning | 0.029308 |
| Neighbors of quercetin | 0.015575 |  |  |  |  | Neighbors of lignin | 0.03082 |
| Neighbors of leaf initiation | 0.015871 |  |  |  |  | Neighbors of phenylpropanoids | 0.032078 |
| Neighbors of G1/S transition checkpoint | 0.016077 |  |  |  |  | Neighbors of CRC | 0.032223 |
| Neighbors of pectinesterase | 0.016148 |  |  |  |  | Neighbors of cell cycle regulator | 0.035958 |
| Neighbors of MUTE | 0.016611 |  |  |  |  | Neighbors of pectinesterase | 0.036159 |
| Neighbors of GNC | 0.01682 |  |  |  |  | Neighbors of carpel development | 0.036335 |
| Neighbors of cytokinin content | 0.016872 |  |  |  |  | Neighbors of xylem loading | 0.036435 |
| Neighbors of CKX3 | 0.018213 |  |  |  |  | Neighbors of HRR | 0.036849 |
| Neighbors of meristem function | 0.018422 |  |  |  |  | Neighbors of Homeo | 0.038851 |
| Neighbors of response to dehydration | 0.01849 |  |  |  |  | Neighbors of MET1 | 0.039836 |
| Neighbors of WUS | 0.018725 |  |  |  |  | Neighbors of inflorescence patterning | 0.040573 |
| Neighbors of tuber development | 0.018823 |  |  |  |  | Neighbors of NADPH | 0.040608 |
| Neighbors of DRM2 | 0.021215 |  |  |  |  | Neighbors of catechin | 0.042117 |
| Neighbors of LOB | 0.021569 |  |  |  |  | Neighbors of intracellular pH | 0.042716 |
| Neighbors of flower patterning | 0.022396 |  |  |  |  | Neighbors of SEP3 | 0.042978 |
| Neighbors of cell homeostasis | 0.022526 |  |  |  |  | Neighbors of AS2 | 0.043065 |
| Neighbors of trichome development | 0.022617 |  |  |  |  | Neighbors of calcium ion homeostasis | 0.043234 |
| Neighbors of photoperiodism, flowering | 0.023459 |  |  |  |  | Neighbors of flower type | 0.043692 |
| Neighbors of water transport | 0.024391 |  |  |  |  | Neighbors of E2F | 0.044538 |
| Neighbors of D-glucose | 0.024523 |  |  |  |  | Neighbors of leaf morphogenesis | 0.044569 |
| Neighbors of lignin | 0.024554 |  |  |  |  | Neighbors of gravitropism | 0.045585 |
| Neighbors of AGL24 | 0.024842 |  |  |  |  | Neighbors of alpha-amylase | 0.046664 |
| Neighbors of sesquiterpenes | 0.025103 |  |  |  |  | Neighbors of amino acid metabolism | 0.048873 |
| Neighbors of fruit set | 0.025212 |  |  |  |  |  |  |
| Neighbors of SEP3 | 0.025997 |  |  |  |  |  |  |
| Neighbors of LFY | 0.02696 |  |  |  |  |  |  |
| Neighbors of response to phosphate starvation | 0.027176 |  |  |  |  |  |  |
| Neighbors of RGL3 | 0.027938 |  |  |  |  |  |  |
| Neighbors of lignification | 0.028225 |  |  |  |  |  |  |
| Neighbors of TT10 | 0.028609 |  |  |  |  |  |  |
| Neighbors of EDS16 | 0.028757 |  |  |  |  |  |  |
| Neighbors of BPEp | 0.029762 |  |  |  |  |  |  |
| Neighbors of proanthocyanidin metabolism | 0.030006 |  |  |  |  |  |  |
| Neighbors of Homeo | 0.030756 |  |  |  |  |  |  |
| Neighbors of WRKY | 0.032036 |  |  |  |  |  |  |
| Neighbors of GA20 | 0.032722 |  |  |  |  |  |  |
| Neighbors of GA9 | 0.032722 |  |  |  |  |  |  |
| Neighbors of anthocyanin metabolism | 0.034094 |  |  |  |  |  |  |
| Neighbors of cytokinin metabolism | 0.034233 |  |  |  |  |  |  |
| Neighbors of HSFA3 | 0.034559 |  |  |  |  |  |  |
| Neighbors of phytoalexin | 0.034763 |  |  |  |  |  |  |
| Neighbors of WRKY46 | 0.034876 |  |  |  |  |  |  |
| Neighbors of cold acclimation | 0.034922 |  |  |  |  |  |  |
| Neighbors of expansin | 0.035061 |  |  |  |  |  |  |
| Neighbors of PRR9 | 0.035129 |  |  |  |  |  |  |
| Neighbors of HRR | 0.035602 |  |  |  |  |  |  |
| Neighbors of primordium initiation | 0.035783 |  |  |  |  |  |  |
| Neighbors of PCNA2 | 0.036607 |  |  |  |  |  |  |
| Neighbors of root differentiation | 0.03691 |  |  |  |  |  |  |
| Neighbors of flower identity | 0.037459 |  |  |  |  |  |  |
| Neighbors of nitrate uptake | 0.0375 |  |  |  |  |  |  |
| Neighbors of GA20OX2 | 0.038692 |  |  |  |  |  |  |
| Neighbors of G1 phase | 0.038821 |  |  |  |  |  |  |
| Neighbors of DME | 0.038839 |  |  |  |  |  |  |
| Neighbors of heavy metal ion | 0.041155 |  |  |  |  |  |  |
| Neighbors of inflorescence patterning | 0.041259 |  |  |  |  |  |  |
| Neighbors of cell fate determination | 0.042495 |  |  |  |  |  |  |
| Neighbors of anther development | 0.044344 |  |  |  |  |  |  |
| Neighbors of PRR5 | 0.04435 |  |  |  |  |  |  |
| Neighbors of intracellular pH | 0.045782 |  |  |  |  |  |  |
| Neighbors of PAP1 | 0.046198 |  |  |  |  |  |  |
| Neighbors of HSFA2 | 0.046359 |  |  |  |  |  |  |
| Neighbors of catechin | 0.04814 |  |  |  |  |  |  |
| Neighbors of HYH | 0.048325 |  |  |  |  |  |  |
| Neighbors of heat tolerance | 0.048479 |  |  |  |  |  |  |
| Neighbors of stomata density | 0.049065 |  |  |  |  |  |  |
| Neighbors of E2F | 0.049563 |  |  |  |  |  |  |

**S6. Pathway studio gene sets and subnetworks differentially expressed between endodormancy and ecodormancy.** “Up- or down-regulated” gene sets are enriched for genes that changed in gene expression, without considering the direction of change of the individual genes. “Up-regulated” and “down-regulated” gene sets are enriched for genes that either increased or decreased in gene expression respectively. Gene sets and subnetworks that were significant at p-value < 0.05 are presented in table.

| **Up- or down-regulated** | |  | **Up-regulated** | |  | **Down-regulated** | |
| --- | --- | --- | --- | --- | --- | --- | --- |
| **Gene sets and subnetworks** | **p-value** |  | **Gene sets and subnetworks** | **p-value** |  | **Gene sets and subnetworks** | **p-value** |
| Neighbors of cellulose | 2.41567E-05 |  | nitrogen metabolism | 0.00020752 |  | lignification | 2.44E-05 |
| Neighbors of CAL | 8.9284E-05 |  | Neighbors of nitrogen metabolism | 0.000276653 |  | Neighbors of lignification | 2.78785E-05 |
| Neighbors of D-Glucurono-D-xylan | 9.38702E-05 |  | Neighbors of HSFA2 | 0.004090635 |  | Neighbors of cellulose | 4.86659E-05 |
| Neighbors of flavonoids | 0.00010237 |  | Neighbors of TAA1 | 0.005313385 |  | Neighbors of flavonoids | 8.2021E-05 |
| Neighbors of H2O | 0.000129087 |  | specification of petal identity | 0.006315193 |  | Neighbors of D-Glucurono-D-xylan | 0.00013704 |
| Neighbors of lignification | 0.000286443 |  | Neighbors of ABA | 0.006522711 |  | wall integrity | 0.000200544 |
| Neighbors of secondary cell wall biosynthesis | 0.000323723 |  | root patterning | 0.007394559 |  | Neighbors of wall integrity | 0.000213048 |
| Neighbors of anthocyanins | 0.000363346 |  | Neighbors of specification of petal identity | 0.007514483 |  | secondary cell wall biosynthesis | 0.000482475 |
| Neighbors of secondary cell wall biosynthesis | 0.000368239 |  | fruit development | 0.008031851 |  | Neighbors of secondary cell wall biosynthesis | 0.000501709 |
| Neighbors of ripening | 0.000378588 |  | Neighbors of HSF | 0.008471104 |  | cellulose biosynthesis | 0.000516946 |
| Neighbors of lignification | 0.000410825 |  | Neighbors of root patterning | 0.008839146 |  | Neighbors of cellulose biosynthesis | 0.000600586 |
| Neighbors of cellulose biosynthesis | 0.00045724 |  | Neighbors of cytokinin | 0.00914057 |  | phenylpropanoid metabolism | 0.000608582 |
| Neighbors of UDP | 0.000497288 |  | Neighbors of nitrate reductase (NADH) | 0.010030428 |  | Neighbors of pectin | 0.000636047 |
| Neighbors of ripening | 0.000591668 |  | Binding partners of LUG | 0.010388733 |  | Neighbors of phenylpropanoid metabolism | 0.000655749 |
| Neighbors of RHD6 | 0.000611258 |  | Neighbors of fruit development | 0.010795193 |  | hypocotyl shape | 0.000779806 |
| Neighbors of cellulose biosynthesis | 0.000654737 |  | Neighbors of phosphatidic acid | 0.012363847 |  | Neighbors of hypocotyl shape | 0.000843869 |
| Neighbors of wall integrity | 0.000899986 |  | Neighbors of GolS2 | 0.013102852 |  | Neighbors of anthocyanins | 0.001147087 |
| Neighbors of wall integrity | 0.001031557 |  | Neighbors of L-tryptophan | 0.013117327 |  | Neighbors of PPi | 0.001240908 |
| Neighbors of lignin | 0.001114822 |  | Binding partners of bZIP transcription factor | 0.013229775 |  | tip growth | 0.001638893 |
| Neighbors of lignin biosynthesis trait | 0.001163431 |  | symport | 0.013851034 |  | Neighbors of tip growth | 0.001799238 |
| Neighbors of expansin | 0.001279616 |  | Binding partners of F-actin | 0.014256069 |  | Neighbors of lignin | 0.002081655 |
| Binding partners of CESA6 | 0.001433958 |  | heat tolerance | 0.014484901 |  | Neighbors of 1,3-xylan | 0.002166165 |
| Neighbors of phytoalexin | 0.001500651 |  | Binding partners of actins | 0.01485959 |  | Binding partners of CESA6 | 0.002386075 |
| Neighbors of lignin biosynthesis trait | 0.00151118 |  | lipid metabolism | 0.015707744 |  | Binding partners of IRX1 | 0.002407625 |
| Binding partners of IRX1 | 0.001642547 |  | Neighbors of D-glucose | 0.015742539 |  | Neighbors of PDF1.2 | 0.003014316 |
| Neighbors of TRY | 0.001678127 |  | Neighbors of symport | 0.016225113 |  | Neighbors of phytoalexin | 0.003261904 |
| Expression targets of SVP | 0.001778101 |  | Neighbors of H2O | 0.016271245 |  | ripening | 0.00366821 |
| Neighbors of pedicel growth | 0.001932228 |  | leaf size | 0.017619776 |  | Binding partners of CESA1 | 0.003727843 |
| Expression targets of SPL3 | 0.002021353 |  | Neighbors of expansin | 0.018185891 |  | Neighbors of K+ | 0.003939296 |
| Neighbors of Flavonol | 0.002187337 |  | response to dehydration | 0.018486782 |  | Neighbors of ripening | 0.004047251 |
| Binding partners of CESA1 | 0.0022231 |  | Neighbors of carotenoids | 0.019805765 |  | defense response | 0.004441717 |
| Neighbors of 1,3-xylan | 0.002223948 |  | Neighbors of lipid metabolism | 0.020666561 |  | Neighbors of UDP | 0.004677188 |
| Neighbors of pedicel growth | 0.002338301 |  | organ formation | 0.020760163 |  | Neighbors of TRY | 0.005230675 |
| Neighbors of aromadendrin | 0.002651087 |  | Neighbors of heat tolerance | 0.021262784 |  | Neighbors of defense response | 0.005306572 |
| Neighbors of anther development | 0.002672571 |  | stem growth | 0.021488519 |  | phosphorelay | 0.005534487 |
| Neighbors of HSFA2 | 0.002836866 |  | Binding partners of actin filament | 0.022526514 |  | Neighbors of EIN2 | 0.005649618 |
| Expression targets of histone H3 | 0.002894116 |  | Neighbors of monosaccharides | 0.022638338 |  | Neighbors of HSP70 | 0.005741877 |
| Neighbors of succinate | 0.003037832 |  | Neighbors of leaf size | 0.022769624 |  | Binding partners of cellulose synthase (GDP-forming) | 0.005759985 |
| Neighbors of meristem growth | 0.00344279 |  | Neighbors of nitrate | 0.022961971 |  | Neighbors of phosphorelay | 0.005837069 |
| Neighbors of pectin | 0.003469873 |  | response to cold | 0.022980294 |  | Neighbors of Proanthocyanidin | 0.005866934 |
| Neighbors of anther development | 0.003485965 |  | response to heat shock | 0.023381941 |  | Neighbors of aromadendrin | 0.00652899 |
| Binding partners of IRX3 | 0.00392768 |  | Neighbors of response to dehydration | 0.024341226 |  | lignin biosynthesis trait | 0.006779067 |
| Neighbors of specification of petal identity | 0.004112628 |  | ripening | 0.02453769 |  | Neighbors of lignin biosynthesis trait | 0.007176472 |
| Neighbors of meristem growth | 0.004132641 |  | anther development | 0.024962852 |  | epidermal cell differentiation | 0.00751733 |
| Neighbors of epidermal cell differentiation | 0.004408905 |  | amino acid metabolism | 0.024965545 |  | pedicel growth | 0.007562101 |
| Neighbors of flower identity | 0.004753571 |  | Neighbors of heat shock protein | 0.025284543 |  | Binding partners of IRX3 | 0.007589086 |
| Neighbors of specification of petal identity | 0.00479142 |  | Neighbors of organ formation | 0.02587752 |  | pathogen interaction | 0.007696565 |
| Neighbors of fruit development | 0.004841944 |  | lignin biosynthesis trait | 0.027023587 |  | Neighbors of epidermal cell differentiation | 0.007830814 |
| Neighbors of ontogeny | 0.005133712 |  | Neighbors of response to heat shock | 0.027225179 |  | Neighbors of pedicel growth | 0.007982736 |
| Neighbors of phenylpropanoid metabolism | 0.005181676 |  | Neighbors of response to cold | 0.027368897 |  | Neighbors of H2O | 0.008100859 |
| Neighbors of flower identity | 0.005223515 |  | Neighbors of amino acid metabolism | 0.028108652 |  | Neighbors of pathogen interaction | 0.008326468 |
| Neighbors of epidermal cell differentiation | 0.005321804 |  | Neighbors of SVP | 0.029037022 |  | Binding partners of CDK | 0.008662986 |
| Neighbors of AGC2-1 | 0.005342414 |  | G2/M transition | 0.029867011 |  | Neighbors of PHYB | 0.009981962 |
| Neighbors of Taxifoliol | 0.005359739 |  | Neighbors of stem growth | 0.030186071 |  | Neighbors of GL2 | 0.010061546 |
| Expression targets of RGA1 | 0.005379665 |  | shoot morphogenesis | 0.030822929 |  | meristem initiation | 0.010526856 |
| Neighbors of GolS2 | 0.005704927 |  | Neighbors of anther development | 0.030932331 |  | membrane depolarization | 0.010812145 |
| Binding partners of bZIP transcription factor | 0.005811405 |  | Neighbors of ripening | 0.031843013 |  | Protein modification targets of MPK6 | 0.010966976 |
| Neighbors of ontogeny | 0.005946429 |  | Neighbors of Na+ | 0.032086517 |  | Neighbors of Coniferyl alcohol | 0.011075538 |
| Binding partners of cellulose synthase (GDP-forming) | 0.005992552 |  | Neighbors of lignin biosynthesis trait | 0.032782014 |  | Neighbors of meristem initiation | 0.011246179 |
| Neighbors of phenylpropanoid metabolism | 0.006017302 |  | Neighbors of GA 20-oxidase | 0.034547673 |  | Neighbors of Cyanidin | 0.011509026 |
| Neighbors of KCS1 | 0.006018826 |  | clathrin-mediated endocytosis | 0.034704883 |  | Neighbors of membrane depolarization | 0.011610345 |
| Neighbors of betaine | 0.006451966 |  | photoperiodism, flowering | 0.035397975 |  | ontogeny | 0.011992422 |
| Neighbors of fruit development | 0.0064834 |  | Neighbors of G2/M transition | 0.037660122 |  | chloroplast relocation | 0.011994828 |
| Neighbors of Proanthocyanidin | 0.006841649 |  | stomatal movement | 0.037743343 |  | turgor | 0.012373871 |
| Neighbors of 4-amino-2,6-dinitrotoluene glucoside | 0.007086122 |  | Neighbors of E2F | 0.037763462 |  | Neighbors of ontogeny | 0.012539667 |
| Neighbors of 4-hydroxylamino-2,6-dinitrotoluene C-glucoside | 0.007086122 |  | Neighbors of NH3 | 0.037809098 |  | Neighbors of Taxifoliol | 0.012586376 |
| Neighbors of 4-hydroxylamino-2,6-dinitrotoluene-O-glucoside | 0.007086122 |  | Neighbors of H+ | 0.037830509 |  | Neighbors of chloroplast relocation | 0.012674953 |
| Neighbors of 2-amino-4,6-dinitrotoluene glucoside | 0.007086122 |  | Neighbors of shoot morphogenesis | 0.037921823 |  | Neighbors of turgor | 0.012796413 |
| Neighbors of 2-hydroxylamino-4,6-dinitrotoluene-C-glucoside | 0.007086122 |  | Neighbors of carbohydrates | 0.039125563 |  | Neighbors of PAD4 | 0.01385741 |
| Neighbors of L-tryptophan | 0.007534444 |  | Neighbors of GA20OX2 | 0.040760922 |  | Neighbors of brassinosteroids | 0.013971328 |
| Binding partners of AP2 | 0.007622251 |  | Neighbors of clathrin-mediated endocytosis | 0.040989861 |  | Neighbors of sinapic alcohol | 0.014526399 |
| Neighbors of AGL24 | 0.007700514 |  | Neighbors of photoperiodism, flowering | 0.041487795 |  | anther development | 0.014672955 |
| Binding partners of helicase | 0.007859945 |  | Binding partners of PKS1 | 0.042498545 |  | cell redox homeostasis | 0.014697181 |
| Expression targets of ABI4 | 0.008154593 |  | Neighbors of COR47 | 0.043005831 |  | Neighbors of CAL | 0.0147654 |
| Neighbors of auxins | 0.008317646 |  | plant growth | 0.043179724 |  | trichome differentiation | 0.014838135 |
| Neighbors of hypocotyl shape | 0.008780597 |  | Neighbors of ABA insensitivity | 0.04321951 |  | Neighbors of EDS1 | 0.014949883 |
| Neighbors of organ formation | 0.008812396 |  | Neighbors of NRT2:1 | 0.045073666 |  | Neighbors of oligosaccharides | 0.014962745 |
| Neighbors of flower color | 0.009378146 |  | miRNA targets of MIR413 | 0.046161485 |  | Neighbors of BAN | 0.015586467 |
| Binding partners of MADS box protein | 0.009469555 |  | cytokinin metabolism | 0.047507885 |  | Neighbors of cell redox homeostasis | 0.015607246 |
| miRNA targets of MIR156A | 0.009794315 |  |  |  |  | Neighbors of anther development | 0.015807845 |
| Neighbors of cell communication | 0.009822179 |  |  |  |  | Neighbors of trichome differentiation | 0.016257689 |
| Neighbors of GA20 | 0.009938936 |  |  |  |  | Neighbors of catechin | 0.016389806 |
| Neighbors of GA9 | 0.009938936 |  |  |  |  | proanthocyanidin metabolism | 0.018453694 |
| Expression targets of MYC2 | 0.009985867 |  |  |  |  | Neighbors of proanthocyanidin metabolism | 0.02004414 |
| Neighbors of SEP3 | 0.010021124 |  |  |  |  | Expression targets of MYC2 | 0.020548361 |
| Binding partners of LUG | 0.010029285 |  |  |  |  | Neighbors of PIN3 | 0.020643787 |
| Neighbors of flower color | 0.010305931 |  |  |  |  | flower color | 0.020882738 |
| Neighbors of hypocotyl shape | 0.010369605 |  |  |  |  | miRNA targets of MIR156A | 0.020955274 |
| Neighbors of BPEp | 0.01074157 |  |  |  |  | trichome branching | 0.021005233 |
| Neighbors of catechin | 0.010837145 |  |  |  |  | plant defense | 0.021287016 |
| Neighbors of organ formation | 0.010939066 |  |  |  |  | pollen tube guidance | 0.021308163 |
| Neighbors of membrane depolarization | 0.01125019 |  |  |  |  | Neighbors of monolignols | 0.021617973 |
| Neighbors of cell communication | 0.011376207 |  |  |  |  | Neighbors of flower color | 0.021699 |
| Neighbors of PP2C | 0.011472016 |  |  |  |  | reproduction | 0.021718069 |
| Binding partners of CDK | 0.011930112 |  |  |  |  | Neighbors of pollen tube guidance | 0.022004755 |
| Neighbors of N-acetyltransferase | 0.012067185 |  |  |  |  | Neighbors of linoleate 13S-lipoxygenase | 0.022035215 |
| Neighbors of tip growth | 0.012783489 |  |  |  |  | Neighbors of trichome branching | 0.022476799 |
| Neighbors of brassinosteroids | 0.012861137 |  |  |  |  | Neighbors of reproduction | 0.022796817 |
| Neighbors of xyloglucan:xyloglucosyl transferase | 0.012921876 |  |  |  |  | organ formation | 0.024044406 |
| Neighbors of root patterning | 0.013177859 |  |  |  |  | Neighbors of plant defense | 0.024091289 |
| Binding partners of AHBP-1B | 0.013403659 |  |  |  |  | cell wall biosynthesis | 0.024472773 |
| Neighbors of cell fate specification | 0.013738666 |  |  |  |  | miRNA targets of MIR397B | 0.025049999 |
| Neighbors of Naringenin | 0.013792317 |  |  |  |  | Neighbors of organ formation | 0.025250124 |
| Neighbors of phosphorelay | 0.014045624 |  |  |  |  | meristem growth | 0.02563714 |
| Neighbors of membrane depolarization | 0.014129796 |  |  |  |  | Neighbors of cell wall biosynthesis | 0.025860192 |
| Neighbors of PDF1.2 | 0.014686443 |  |  |  |  | Neighbors of meristem growth | 0.026620538 |
| Neighbors of CRC | 0.015067151 |  |  |  |  | Neighbors of auxins | 0.026663618 |
| Neighbors of root patterning | 0.01533856 |  |  |  |  | Protein modification targets of MPK4 | 0.026742348 |
| Binding partners of TTG1 | 0.015818071 |  |  |  |  | Binding partners of plus-end-directed kinesin ATPase | 0.027141911 |
| Neighbors of pigment biosynthesis | 0.015998114 |  |  |  |  | miRNA targets of MIR156C | 0.027228462 |
| Neighbors of tip growth | 0.016451195 |  |  |  |  | miRNA targets of MIR156B | 0.027228462 |
| Neighbors of Cyanidin | 0.016464272 |  |  |  |  | miRNA targets of MIR156F | 0.027228462 |
| Neighbors of phosphorelay | 0.016629951 |  |  |  |  | miRNA targets of MIR156D | 0.027228462 |
| Neighbors of cell fate specification | 0.017076632 |  |  |  |  | miRNA targets of MIR156E | 0.027228462 |
| Neighbors of GLU1 | 0.017637812 |  |  |  |  | Expression targets of ETR1 | 0.027430603 |
| Neighbors of nitrate uptake | 0.017788616 |  |  |  |  | Neighbors of PP2C | 0.028473409 |
| Protein modification targets of MPK4 | 0.018073627 |  |  |  |  | Neighbors of Naringenin | 0.029287486 |
| Neighbors of meristem initiation | 0.018239448 |  |  |  |  | miRNA targets of MIR156G | 0.030582225 |
| Expression targets of RPL | 0.018276391 |  |  |  |  | Expression targets of BRI1 | 0.031884487 |
| Neighbors of COR47 | 0.018369969 |  |  |  |  | Neighbors of TTG2 | 0.032165695 |
| Neighbors of pigment biosynthesis | 0.018394873 |  |  |  |  | Neighbors of Flavonol | 0.032735945 |
| Protein modification targets of MPK6 | 0.01865963 |  |  |  |  | Neighbors of gibberellin | 0.034691084 |
| Expression targets of IPK2BETA | 0.018855896 |  |  |  |  | cell communication | 0.035388405 |
| Neighbors of HSP70 | 0.019427327 |  |  |  |  | Neighbors of Putrescine | 0.035617998 |
| Neighbors of nitrate uptake | 0.019494478 |  |  |  |  | Neighbors of BGL2 | 0.035824988 |
| Neighbors of quercetin | 0.019543797 |  |  |  |  | Neighbors of cell communication | 0.036745664 |
| Neighbors of Putrescine | 0.019602079 |  |  |  |  | auxin polar transport | 0.036986585 |
| Neighbors of BGL2 | 0.019948155 |  |  |  |  | Neighbors of RHD6 | 0.037007558 |
| Neighbors of AS2 | 0.020474928 |  |  |  |  | Neighbors of MAP3K | 0.038250395 |
| Neighbors of cell fate determination | 0.021066366 |  |  |  |  | Neighbors of auxin polar transport | 0.03878532 |
| miRNA targets of MIR156H | 0.021449128 |  |  |  |  | pigment biosynthesis | 0.0402564 |
| Neighbors of phosphate import | 0.021832351 |  |  |  |  | Neighbors of transmembrane receptor protein kinase | 0.040619023 |
| Neighbors of inflorescence patterning | 0.02226988 |  |  |  |  | Neighbors of succinate | 0.040648455 |
| Neighbors of meristem initiation | 0.023137838 |  |  |  |  | Neighbors of DFR | 0.041581399 |
| Neighbors of PIN1 | 0.023344516 |  |  |  |  | systemic acquired resistance | 0.041842057 |
| Neighbors of defense response | 0.023378925 |  |  |  |  | Neighbors of pigment biosynthesis | 0.041903723 |
| Neighbors of root development | 0.023737681 |  |  |  |  | Binding partners of EREBP | 0.042194372 |
| Neighbors of TAA1 | 0.023783087 |  |  |  |  | Binding partners of thioredoxin | 0.042275946 |
| Neighbors of ETR1 | 0.02404336 |  |  |  |  | Expression targets of bZIP transcription factor | 0.04295942 |
| Neighbors of inflorescence patterning | 0.024049424 |  |  |  |  | meristem identity | 0.043029516 |
| Neighbors of SVP | 0.024296504 |  |  |  |  | miRNA targets of MIR156H | 0.043895279 |
| Neighbors of PPi | 0.025389423 |  |  |  |  | Neighbors of systemic acquired resistance | 0.044131743 |
| Neighbors of cell fate determination | 0.025519033 |  |  |  |  | Neighbors of AS2 | 0.044448944 |
| Neighbors of meristem identity | 0.025636726 |  |  |  |  | Neighbors of actin filament | 0.044698718 |
| Expression targets of FT | 0.025649478 |  |  |  |  | Neighbors of meristem identity | 0.045555261 |
| Neighbors of phosphate import | 0.025723449 |  |  |  |  | root hair tip growth | 0.045653905 |
| miRNA targets of MIR164A | 0.0263712 |  |  |  |  | Neighbors of root hair tip growth | 0.046649451 |
| Neighbors of linoleate 13S-lipoxygenase | 0.026473617 |  |  |  |  | Neighbors of MYBR1 | 0.047708043 |
| Neighbors of ethylene | 0.026771046 |  |  |  |  | Neighbors of ROS | 0.048873475 |
| Neighbors of HSFA3 | 0.027260119 |  |  |  |  | Binding partners of TIFY1 | 0.049156968 |
| Neighbors of TT10 | 0.028166901 |  |  |  |  | trichome development | 0.049606777 |
| Neighbors of ANT | 0.028961701 |  |  |  |  |  |  |
| Neighbors of proanthocyanidin metabolism | 0.029107739 |  |  |  |  |  |  |
| Neighbors of suberin | 0.029376264 |  |  |  |  |  |  |
| Protein modification targets of PDK1 | 0.030044537 |  |  |  |  |  |  |
| Neighbors of shoot growth | 0.030462748 |  |  |  |  |  |  |
| Neighbors of H+ | 0.030598058 |  |  |  |  |  |  |
| Neighbors of PIN4 | 0.031053633 |  |  |  |  |  |  |
| Neighbors of meristem identity | 0.031731763 |  |  |  |  |  |  |
| Neighbors of BAN | 0.032200026 |  |  |  |  |  |  |
| Neighbors of stem growth | 0.03264742 |  |  |  |  |  |  |
| Neighbors of nitrogen metabolism | 0.032806246 |  |  |  |  |  |  |
| Neighbors of WOX5 | 0.032950802 |  |  |  |  |  |  |
| Neighbors of proanthocyanidin metabolism | 0.034139873 |  |  |  |  |  |  |
| Expression targets of LFY | 0.034232538 |  |  |  |  |  |  |
| Binding partners of EREBP | 0.034720409 |  |  |  |  |  |  |
| Neighbors of pathogen interaction | 0.035431193 |  |  |  |  |  |  |
| Neighbors of gibberellin | 0.035882722 |  |  |  |  |  |  |
| Neighbors of cutin | 0.036147991 |  |  |  |  |  |  |
| Neighbors of intracellular pH | 0.036202698 |  |  |  |  |  |  |
| Binding partners of ARAC1 | 0.036892327 |  |  |  |  |  |  |
| Expression targets of Myb-factors | 0.037426958 |  |  |  |  |  |  |
| Neighbors of IAA19 | 0.038071957 |  |  |  |  |  |  |
| Neighbors of plant defense | 0.038221974 |  |  |  |  |  |  |
| Neighbors of HSF | 0.038320193 |  |  |  |  |  |  |
| Neighbors of nitrogen metabolism | 0.038636789 |  |  |  |  |  |  |
| Neighbors of shoot growth | 0.039222251 |  |  |  |  |  |  |
| Neighbors of panicle type | 0.03941655 |  |  |  |  |  |  |
| Neighbors of glucoxylan | 0.040316458 |  |  |  |  |  |  |
| Expression targets of PAP1 | 0.04052572 |  |  |  |  |  |  |
| Neighbors of intracellular pH | 0.040578109 |  |  |  |  |  |  |
| Neighbors of leaf size | 0.040800754 |  |  |  |  |  |  |
| Neighbors of monolignols | 0.04148542 |  |  |  |  |  |  |
| Neighbors of root development | 0.04150958 |  |  |  |  |  |  |
| Neighbors of phytohormone | 0.041912223 |  |  |  |  |  |  |
| Neighbors of GA20OX2 | 0.041978849 |  |  |  |  |  |  |
| Neighbors of defense response | 0.042546106 |  |  |  |  |  |  |
| Neighbors of phenylpropanoids | 0.042620951 |  |  |  |  |  |  |
| Neighbors of cytokinin content | 0.042749843 |  |  |  |  |  |  |
| Neighbors of stem growth | 0.04291774 |  |  |  |  |  |  |
| Neighbors of TFL1 | 0.042971022 |  |  |  |  |  |  |
| Neighbors of phosphatidic acid | 0.043360974 |  |  |  |  |  |  |
| Neighbors of response to dehydration | 0.04391927 |  |  |  |  |  |  |
| Neighbors of GSTF8 | 0.043941632 |  |  |  |  |  |  |
| Neighbors of panicle type | 0.044040751 |  |  |  |  |  |  |
| Neighbors of cuticle development | 0.044244896 |  |  |  |  |  |  |
| Neighbors of pathogen interaction | 0.045067675 |  |  |  |  |  |  |
| Neighbors of symport | 0.045289303 |  |  |  |  |  |  |
| Neighbors of GA 20-oxidase | 0.045510706 |  |  |  |  |  |  |
| Neighbors of turgor | 0.045652058 |  |  |  |  |  |  |
| Neighbors of cytokinin content | 0.046423419 |  |  |  |  |  |  |
| Neighbors of PAD4 | 0.047618534 |  |  |  |  |  |  |
| Binding partners of ER | 0.048985303 |  |  |  |  |  |  |
| Neighbors of leaf size | 0.049088394 |  |  |  |  |  |  |
| Neighbors of cuticle development | 0.049546948 |  |  |  |  |  |  |
| Neighbors of turgor | 0.049643709 |  |  |  |  |  |  |
| Neighbors of WRKY22 | 0.049650499 |  |  |  |  |  |  |
| Neighbors of Brassinolide | 0.049800482 |  |  |  |  |  |  |
| Neighbors of symport | 0.049892234 |  |  |  |  |  |  |
| Neighbors of FD | 0.049894476 |  |  |  |  |  |  |

**S7. Pathway studio gene sets and subnetworks differentially expressed between ecodormancy and bud flush.** “Up- or down-regulated” gene sets are enriched for genes that changed in gene expression, without considering the direction of change of the individual genes. “Up-regulated” and “down-regulated” gene sets are enriched for genes that either increased or decreased in gene expression respectively. Gene sets and subnetworks that were significant at p-value < 0.05 are presented in table.

| **Up- or down-regulated** | |  | **Up-regulated** | |  | **Down-regulated** | |
| --- | --- | --- | --- | --- | --- | --- | --- |
| **Gene sets and subnetworks** | **p-value** |  | **Gene sets and subnetworks** | **p-value** |  | **Gene sets and subnetworks** | **p-value** |
| Neighbors of anthocyanins | 1.09071E-05 |  | Neighbors of anthocyanins | 9.91112E-05 |  | Neighbors of fruit growth | 0.00117325 |
| Neighbors of DNA replication initiation | 0.000144153 |  | Neighbors of DNA replication initiation | 0.000125989 |  | Neighbors of fruit growth | 0.00155934 |
| Neighbors of DNA replication initiation | 0.000147681 |  | Neighbors of DNA replication initiation | 0.000127186 |  | Neighbors of HSFA3 | 0.002116932 |
| Neighbors of H2O | 0.000500006 |  | Neighbors of DNA replication | 0.00021681 |  | Neighbors of response to heat shock | 0.002558392 |
| Neighbors of Flavonol | 0.001111094 |  | Neighbors of DNA replication | 0.000295955 |  | Neighbors of response to heat shock | 0.00291362 |
| Neighbors of monosaccharides | 0.001164382 |  | Neighbors of cytokinesis | 0.000421107 |  | Neighbors of PAD4 | 0.003617441 |
| Neighbors of GA-signaling | 0.001593649 |  | Neighbors of cytokinesis | 0.000537238 |  | Neighbors of stem growth | 0.004883079 |
| Neighbors of 4-amino-2,6-dinitrotoluene glucoside | 0.001671416 |  | Neighbors of Flavonol | 0.000927317 |  | Neighbors of H+ | 0.005045077 |
| Neighbors of 4-hydroxylamino-2,6-dinitrotoluene C-glucoside | 0.001671416 |  | Neighbors of UDP | 0.001211898 |  | Neighbors of phosphate import | 0.005256772 |
| Neighbors of 4-hydroxylamino-2,6-dinitrotoluene-O-glucoside | 0.001671416 |  | Neighbors of pigmentation | 0.001270061 |  | Neighbors of Tricarboxylic acid cycle | 0.005639786 |
| Neighbors of 2-amino-4,6-dinitrotoluene glucoside | 0.001671416 |  | Binding partners of plus-end-directed kinesin ATPase | 0.001374851 |  | Neighbors of response to dehydration | 0.006482934 |
| Neighbors of 2-hydroxylamino-4,6-dinitrotoluene-C-glucoside | 0.001671416 |  | Neighbors of aromadendrin | 0.00138762 |  | Neighbors of Tricarboxylic acid cycle | 0.00649832 |
| Neighbors of UDP | 0.001841093 |  | Neighbors of pigmentation | 0.001530125 |  | Neighbors of phosphate import | 0.007066312 |
| Neighbors of lignification | 0.001953753 |  | Neighbors of 4-amino-2,6-dinitrotoluene glucoside | 0.001656683 |  | Neighbors of stem growth | 0.007235991 |
| Neighbors of aromadendrin | 0.001970831 |  | Neighbors of 4-hydroxylamino-2,6-dinitrotoluene C-glucoside | 0.001656683 |  | Binding partners of leucine zipper | 0.00770558 |
| Neighbors of GA-signaling | 0.0020504 |  | Neighbors of 4-hydroxylamino-2,6-dinitrotoluene-O-glucoside | 0.001656683 |  | Neighbors of suberin | 0.009250973 |
| Neighbors of xyloglucan:xyloglucosyl transferase | 0.002345376 |  | Neighbors of 2-amino-4,6-dinitrotoluene glucoside | 0.001656683 |  | Neighbors of catalase | 0.009570966 |
| Neighbors of lignification | 0.002473453 |  | Neighbors of 2-hydroxylamino-4,6-dinitrotoluene-C-glucoside | 0.001656683 |  | Neighbors of response to dehydration | 0.009874261 |
| Neighbors of CAL | 0.002695138 |  | Neighbors of flavonoids | 0.001884216 |  | Neighbors of HSF1 | 0.010134361 |
| Neighbors of response to heat shock | 0.002839997 |  | Neighbors of RBR1 | 0.001889358 |  | miRNA targets of MIR169A | 0.010346412 |
| Neighbors of turgor | 0.002857599 |  | Neighbors of microtubule | 0.002008502 |  | Neighbors of Rice blast | 0.010540927 |
| Neighbors of GA20 | 0.003061774 |  | Neighbors of microtubule cytoskeleton assembly | 0.002573377 |  | Neighbors of GolS2 | 0.010599623 |
| Neighbors of GA9 | 0.003061774 |  | Neighbors of Taxifoliol | 0.003252022 |  | Neighbors of lignification | 0.010874951 |
| Neighbors of response to heat shock | 0.003170645 |  | Neighbors of meristem organization | 0.003370265 |  | Neighbors of Rice blast | 0.011271173 |
| Neighbors of turgor | 0.003205151 |  | Neighbors of microtubule cytoskeleton assembly | 0.003373551 |  | Neighbors of GA-signaling | 0.011332241 |
| Binding partners of leucine zipper | 0.003215311 |  | Neighbors of meristem organization | 0.003507603 |  | Neighbors of SLY1 | 0.011703001 |
| Neighbors of flavonoids | 0.003536671 |  | Binding partners of CDK | 0.003507781 |  | Neighbors of xylem loading | 0.011972866 |
| Neighbors of Cd2+ | 0.004196698 |  | Neighbors of PAI2 | 0.003648228 |  | Neighbors of PP2C | 0.012963566 |
| Neighbors of SLY1 | 0.004510597 |  | Neighbors of turgor | 0.00368056 |  | Neighbors of anthocyanins | 0.013683681 |
| Protein modification targets of MPK6 | 0.004517365 |  | Neighbors of turgor | 0.00377075 |  | Neighbors of ABA | 0.013884639 |
| Neighbors of PAI2 | 0.004751798 |  | Neighbors of Cyanidin | 0.004324708 |  | Neighbors of xylem loading | 0.013945653 |
| Neighbors of Taxifoliol | 0.004838847 |  | Neighbors of DNA recombination | 0.004533997 |  | Neighbors of lignification | 0.013974849 |
| Neighbors of stem growth | 0.005198891 |  | Neighbors of brassinosteroids | 0.005316723 |  | Neighbors of phosphatidic acid | 0.014264729 |
| Neighbors of H+ | 0.005345185 |  | Binding partners of histone H3 | 0.005834429 |  | Neighbors of abiotic stress | 0.014299158 |
| Neighbors of hypocotyl shape | 0.005588176 |  | Neighbors of DNA recombination | 0.006416036 |  | Neighbors of GA-signaling | 0.014411275 |
| Neighbors of KCS1 | 0.005717439 |  | Neighbors of root differentiation | 0.006525969 |  | Neighbors of response to cold | 0.015522219 |
| Neighbors of Cyanidin | 0.005759154 |  | Expression targets of HB-8 | 0.006908282 |  | Neighbors of EDS1 | 0.016197816 |
| Neighbors of succinate | 0.006395905 |  | Binding partners of microtubule | 0.007033 |  | Neighbors of tuber development | 0.016689017 |
| Binding partners of RING finger motif | 0.006531013 |  | Neighbors of root differentiation | 0.007620338 |  | Neighbors of oxidative stress | 0.017307256 |
| Neighbors of hypocotyl shape | 0.006552823 |  | Expression targets of BRI1 | 0.008127888 |  | Neighbors of HSP70 | 0.018140824 |
| Neighbors of stem growth | 0.006619722 |  | Neighbors of H2O | 0.008181169 |  | Neighbors of response to cold | 0.018960839 |
| Neighbors of nitrate uptake | 0.006657367 |  | Neighbors of flower color | 0.008396758 |  | Neighbors of tuber development | 0.0192289 |
| Protein modification targets of MPK4 | 0.006813146 |  | Neighbors of Proanthocyanidin | 0.008803305 |  | Neighbors of protein processing | 0.020609774 |
| Neighbors of nitrate uptake | 0.007368784 |  | Binding partners of CDC2 | 0.008904633 |  | Neighbors of TFL1 | 0.020807396 |
| Neighbors of DNA replication | 0.007646203 |  | Neighbors of flower color | 0.009215618 |  | Binding partners of CBL1 | 0.02147304 |
| Neighbors of epidermis development | 0.007699089 |  | Expression targets of PIF4 | 0.009593211 |  | Neighbors of Plant morphology | 0.021537317 |
| Neighbors of betaine | 0.007759182 |  | Neighbors of stomata density | 0.009909752 |  | Neighbors of protein folding | 0.022134854 |
| Neighbors of phosphatidic acid | 0.007942031 |  | Neighbors of auxins | 0.009920713 |  | Neighbors of monosaccharides | 0.022145767 |
| Neighbors of epidermis development | 0.008159515 |  | Binding partners of basic-helix-loop-helix protein | 0.010867998 |  | Neighbors of abiotic stress | 0.022318587 |
| Neighbors of monolignols | 0.00859293 |  | Neighbors of stomata density | 0.011039471 |  | Binding partners of photosystem II reaction center | 0.023154396 |
| Neighbors of metaphase | 0.009131433 |  | Neighbors of catechin | 0.011210887 |  | Neighbors of heat tolerance | 0.023213884 |
| Neighbors of sinapic alcohol | 0.009233899 |  | Neighbors of CAL | 0.011988342 |  | Neighbors of protein processing | 0.02483293 |
| Neighbors of DNA replication | 0.009249068 |  | Neighbors of Naringenin | 0.01357256 |  | Neighbors of protein folding | 0.025271592 |
| Neighbors of phosphate import | 0.009352578 |  | Neighbors of urea | 0.013698794 |  | Neighbors of oxidative stress | 0.025902151 |
| Neighbors of root patterning | 0.009391418 |  | Neighbors of FWA | 0.014355676 |  | Neighbors of Plant morphology | 0.026681591 |
| Neighbors of stomata density | 0.009897522 |  | Neighbors of lignin | 0.01502073 |  | Neighbors of LTI78 | 0.02772223 |
| Neighbors of lignin | 0.009921812 |  | Neighbors of lignification | 0.015056395 |  | Neighbors of CAT3 | 0.028944656 |
| Neighbors of metaphase | 0.010076182 |  | Neighbors of FIS2 | 0.015367418 |  | Neighbors of drought tolerance | 0.030247677 |
| Neighbors of GDP | 0.010178575 |  | Neighbors of cell expansion | 0.015947124 |  | Neighbors of P5CS1 | 0.030607006 |
| Neighbors of osmotic pressure | 0.010415247 |  | Neighbors of cell polarity | 0.016325018 |  | Neighbors of heat shock protein | 0.031316271 |
| Neighbors of phosphate import | 0.010449576 |  | Neighbors of NPH4 | 0.016407841 |  | Neighbors of hypocotyl shape | 0.031809577 |
| Neighbors of root patterning | 0.010676113 |  | Neighbors of cell redox homeostasis | 0.017070933 |  | Neighbors of heat tolerance | 0.032731852 |
| Neighbors of DFR | 0.010692878 |  | Neighbors of MEA | 0.018388532 |  | Neighbors of leaf growth | 0.034864282 |
| Neighbors of flower color | 0.011064493 |  | Neighbors of lignification | 0.018458954 |  | Neighbors of stomatal movement | 0.0349034 |
| Neighbors of osmotic pressure | 0.011073155 |  | Neighbors of metaphase | 0.018474196 |  | Binding partners of CCAAT factors | 0.035271669 |
| Neighbors of flower color | 0.011369241 |  | Neighbors of cell redox homeostasis | 0.018648386 |  | Neighbors of LEA | 0.035949303 |
| Neighbors of cytokinesis | 0.011455397 |  | Neighbors of cell polarity | 0.018879249 |  | Neighbors of fruit development | 0.036136985 |
| Neighbors of stomata density | 0.011633212 |  | Neighbors of metaphase | 0.018906232 |  | Neighbors of succinate | 0.036917989 |
| Neighbors of phosphoribosylanthranilate isomerase | 0.011745601 |  | Neighbors of nucleotide-excision repair | 0.019123308 |  | Neighbors of lignin content | 0.037312876 |
| Neighbors of FLS1 | 0.012681068 |  | Neighbors of hypocotyl shape | 0.019460046 |  | Neighbors of hypocotyl shape | 0.037457301 |
| Neighbors of flower identity | 0.01285919 |  | Neighbors of hypocotyl shape | 0.019874046 |  | Neighbors of transpiration | 0.037813643 |
| Neighbors of quercetin | 0.012910003 |  | Expression targets of SHY2 | 0.020490868 |  | Neighbors of acidification | 0.037822136 |
| Binding partners of helicase | 0.012975308 |  | Neighbors of nucleotide-excision repair | 0.021038582 |  | miRNA targets of MIR169B | 0.037884673 |
| Neighbors of cytokinesis | 0.013245885 |  | Neighbors of sterols | 0.021246641 |  | miRNA targets of MIR169C | 0.037884673 |
| Binding partners of plus-end-directed kinesin ATPase | 0.013370202 |  | Neighbors of cell expansion | 0.021965833 |  | Neighbors of nitrate reductase (NADH) | 0.037932007 |
| Neighbors of cell redox homeostasis | 0.013415154 |  | Neighbors of SHY2 | 0.021993136 |  | Neighbors of chitinase | 0.038237264 |
| Neighbors of flower identity | 0.013544834 |  | Neighbors of WOX5 | 0.02286648 |  | Neighbors of APX2 | 0.039041567 |
| Neighbors of fruit growth | 0.014611096 |  | Binding partners of SGT1B | 0.023526272 |  | Neighbors of CBF1 | 0.039264446 |
| Expression targets of HFR1 | 0.014626585 |  | Neighbors of MET1 | 0.024127583 |  | Neighbors of HSFA2 | 0.040468849 |
| Neighbors of cell redox homeostasis | 0.014761742 |  | Expression targets of PIL6 | 0.025335505 |  | Neighbors of salicylate | 0.04098675 |
| Neighbors of leaf size | 0.01560782 |  | Neighbors of negative gravitropism | 0.02703946 |  | Neighbors of nitrate | 0.041154635 |
| Binding partners of SGT1B | 0.015749008 |  | Neighbors of xyloglucan:xyloglucosyl transferase | 0.02725568 |  | Neighbors of petal development | 0.041226937 |
| Neighbors of HSF | 0.01586192 |  | Neighbors of negative gravitropism | 0.028622816 |  | Expression targets of ABI4 | 0.041562475 |
| Expression targets of BRI1 | 0.01611733 |  | Neighbors of cell cycle | 0.028870885 |  | Neighbors of carbohydrates | 0.042586976 |
| Neighbors of pigmentation | 0.016680213 |  | Neighbors of xylem development | 0.029648183 |  | Neighbors of acidification | 0.043753507 |
| Expression targets of ABI4 | 0.016697119 |  | Neighbors of xylem development | 0.03178177 |  | Expression targets of RGA1 | 0.043965449 |
| Neighbors of fruit growth | 0.01697964 |  | Neighbors of glucosinolate biosynthesis | 0.032631388 |  | Expression targets of PAD4 | 0.044122957 |
| Binding partners of chaperones | 0.017103284 |  | Neighbors of DWF4 | 0.032669369 |  | Neighbors of non-selective vesicle budding | 0.044880194 |
| Expression targets of histone H3 | 0.017486723 |  | Expression targets of E2F | 0.032893633 |  | Neighbors of psbB | 0.044914007 |
| Neighbors of catechin | 0.018044017 |  | Neighbors of indoleacetate | 0.033307618 |  | Neighbors of lignin content | 0.044944906 |
| Binding partners of LEC1 | 0.018189184 |  | Neighbors of G2/M transition | 0.03338303 |  | Neighbors of Flavonol | 0.045410818 |
| Neighbors of PIN3 | 0.018210527 |  | Neighbors of maintenance of DNA methylation | 0.033388454 |  | Neighbors of fruit development | 0.04587893 |
| Neighbors of protein folding | 0.018271192 |  | Neighbors of CUC2 | 0.03349219 |  | Neighbors of drought tolerance | 0.046407624 |
| Neighbors of HSFA3 | 0.018479542 |  | Binding partners of chaperones | 0.033754497 |  | Neighbors of spermidine | 0.046675908 |
| Neighbors of leaf size | 0.018588938 |  | Neighbors of phenylpropanoid metabolism | 0.033865712 |  | Neighbors of leaf growth | 0.047656801 |
| miRNA targets of MIR413 | 0.018930268 |  | Neighbors of response to gravity | 0.034577174 |  | Binding partners of AHP1 | 0.048098684 |
| Neighbors of GolS2 | 0.019023777 |  | Neighbors of maintenance of DNA methylation | 0.035112851 |  | Neighbors of petal development | 0.048497011 |
| Binding partners of CO | 0.019446116 |  | Neighbors of glucosinolate biosynthesis | 0.035573376 |  | Neighbors of HSF | 0.048853397 |
| Neighbors of Naringenin | 0.019457817 |  | Neighbors of anaphase | 0.035599843 |  | Neighbors of CO2 | 0.049214859 |
| Binding partners of histone H3 | 0.019569357 |  | Neighbors of phenylpropanoid metabolism | 0.035664495 |  | Neighbors of transpiration | 0.049655192 |
| Neighbors of L-phenylalanine | 0.019638832 |  | Neighbors of nuclear division | 0.03623249 |  | Neighbors of non-selective vesicle budding | 0.049684905 |
| Neighbors of pigmentation | 0.019678005 |  | Neighbors of response to gravity | 0.036489793 |  | Neighbors of PR1 | 0.049953395 |
| Neighbors of fruit set | 0.020247654 |  | Neighbors of pollen recognition | 0.036703252 |  |  |  |
| Neighbors of protein folding | 0.020773882 |  | Neighbors of cellulose biosynthesis | 0.036916199 |  |  |  |
| Neighbors of HSF1 | 0.020865934 |  | Neighbors of G2/M transition | 0.037283911 |  |  |  |
| Expression targets of RGA1 | 0.020983309 |  | Neighbors of pollen recognition | 0.038375697 |  |  |  |
| Neighbors of response to dehydration | 0.021053398 |  | Neighbors of cell cycle | 0.038561405 |  |  |  |
| Neighbors of beta-galactosidase | 0.021432435 |  | Neighbors of HRR | 0.038640513 |  |  |  |
| Expression targets of HB-8 | 0.02183117 |  | Neighbors of anaphase | 0.038867459 |  |  |  |
| Neighbors of fruit set | 0.021933855 |  | Neighbors of nuclear division | 0.039320038 |  |  |  |
| Neighbors of meristem organization | 0.023620819 |  | Neighbors of HRR | 0.039448184 |  |  |  |
| Neighbors of PP2C | 0.024785211 |  | Neighbors of protein folding | 0.039663129 |  |  |  |
| Neighbors of microtubule cytoskeleton assembly | 0.025597681 |  | Neighbors of root development | 0.041024132 |  |  |  |
| Neighbors of auxins | 0.026046445 |  | Neighbors of anthesis | 0.041283343 |  |  |  |
| Neighbors of meristem organization | 0.026165162 |  | Neighbors of sterol biosynthesis | 0.041487535 |  |  |  |
| Neighbors of response to dehydration | 0.026334218 |  | Neighbors of gravitropism | 0.041980959 |  |  |  |
| Neighbors of MP | 0.027441593 |  | Neighbors of protein folding | 0.044596494 |  |  |  |
| Neighbors of glucosinolate biosynthesis | 0.028059635 |  | Neighbors of callus development | 0.044784223 |  |  |  |
| Neighbors of microtubule cytoskeleton assembly | 0.029513338 |  | Neighbors of callus development | 0.04479227 |  |  |  |
| Neighbors of tuber development | 0.030847807 |  | Neighbors of anthesis | 0.044976965 |  |  |  |
| Neighbors of amino acid metabolism | 0.031063456 |  | Neighbors of cellulose biosynthesis | 0.045199159 |  |  |  |
| Neighbors of glucosinolate biosynthesis | 0.031560669 |  | Neighbors of stomata yield | 0.045672514 |  |  |  |
| Binding partners of microtubule | 0.032423728 |  | Neighbors of secondary cell wall biosynthesis | 0.046459004 |  |  |  |
| Neighbors of tuber development | 0.032462045 |  | Binding partners of CO | 0.047368405 |  |  |  |
| Neighbors of amino acid metabolism | 0.033398806 |  | Neighbors of sterol biosynthesis | 0.047497033 |  |  |  |
| Neighbors of chitinase | 0.034284918 |  | Neighbors of gravitropism | 0.047581629 |  |  |  |
| Neighbors of brassinosteroids | 0.034371535 |  | Neighbors of meristem initiation | 0.047906235 |  |  |  |
| Neighbors of sterols | 0.034905243 |  | Neighbors of secondary cell wall biosynthesis | 0.048506267 |  |  |  |
| Neighbors of PIF3 | 0.035063604 |  | Neighbors of amino acid metabolism | 0.049906566 |  |  |  |
| Neighbors of G2/M transition | 0.035083495 |  | Neighbors of proanthocyanidin metabolism | 0.049956243 |  |  |  |
| Neighbors of alkaline phosphatase | 0.035677823 |  |  |  |  |  |  |
| Neighbors of Proanthocyanidin | 0.036077925 |  |  |  |  |  |  |
| miRNA targets of ath-miR1886 | 0.036297525 |  |  |  |  |  |  |
| Neighbors of cholesterol | 0.036763122 |  |  |  |  |  |  |
| Neighbors of cell polarity | 0.037411518 |  |  |  |  |  |  |
| Neighbors of G2/M transition | 0.038286179 |  |  |  |  |  |  |
| Neighbors of PAD4 | 0.038633702 |  |  |  |  |  |  |
| Expression targets of PIF4 | 0.039121789 |  |  |  |  |  |  |
| Neighbors of cell polarity | 0.040692469 |  |  |  |  |  |  |
| Neighbors of D-glucose | 0.042604136 |  |  |  |  |  |  |
| Expression targets of JKD | 0.043397936 |  |  |  |  |  |  |
| Neighbors of nuclear division | 0.043973439 |  |  |  |  |  |  |
| Neighbors of Sinapinic acid | 0.044277057 |  |  |  |  |  |  |
| Neighbors of MYB23 | 0.045080473 |  |  |  |  |  |  |
| Neighbors of L-tryptophan | 0.045090102 |  |  |  |  |  |  |
| Neighbors of nuclear division | 0.046168632 |  |  |  |  |  |  |
| Neighbors of cell expansion | 0.046349593 |  |  |  |  |  |  |
| Neighbors of meristem initiation | 0.046391282 |  |  |  |  |  |  |
| Neighbors of response to cold | 0.04643499 |  |  |  |  |  |  |
| Binding partners of CCAAT factors | 0.047342702 |  |  |  |  |  |  |
| Neighbors of Tricarboxylic acid cycle | 0.047988959 |  |  |  |  |  |  |
| Neighbors of vacuolar storage | 0.048054471 |  |  |  |  |  |  |
| Neighbors of glucoxylan | 0.048253621 |  |  |  |  |  |  |
| Neighbors of maintenance of DNA methylation | 0.048261372 |  |  |  |  |  |  |
| Binding partners of CBL1 | 0.048386455 |  |  |  |  |  |  |
| Neighbors of callus initiation | 0.048521575 |  |  |  |  |  |  |
| Neighbors of Coniferyl alcohol | 0.049414926 |  |  |  |  |  |  |
