# Supplementary material for: Comprehensive Transcriptome Analyses Reveal Differential Gene Expression Profiles of Camellia sinensis Axillary Buds at Para-, Endo-, Ecodormancy, and Bud Flush Stages
Source: Front Plant Sci. 2017 Apr 18;8:553. doi: 10.3389/fpls.2017.00553 (PMC5394108; doi:10.3389/fpls.2017.00553)
Supplement: Supplementary file 7 [file Data_Sheet_7.docx]

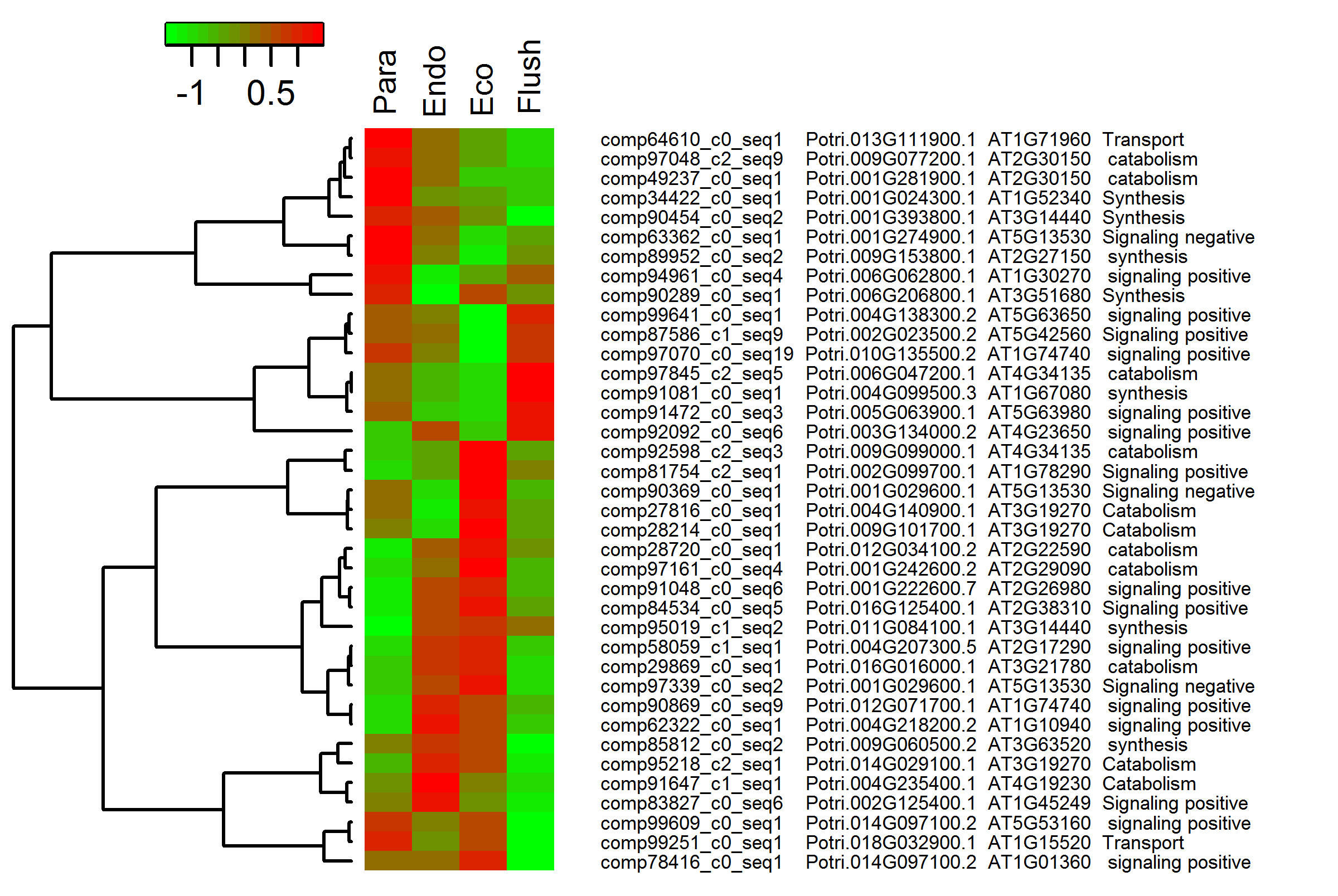


S1 Expression profiling analysis of abscisic acid-associated differentially expressed genes. The information listed on the right of heat map are the transcripts’ name in this study, the transcript’s annotation by poplar protein database, the transcript’s annotation by *Arabidopsis* protein database, and hormone function. Red indicates high relative gene expression and green indicates low relative gene expression.


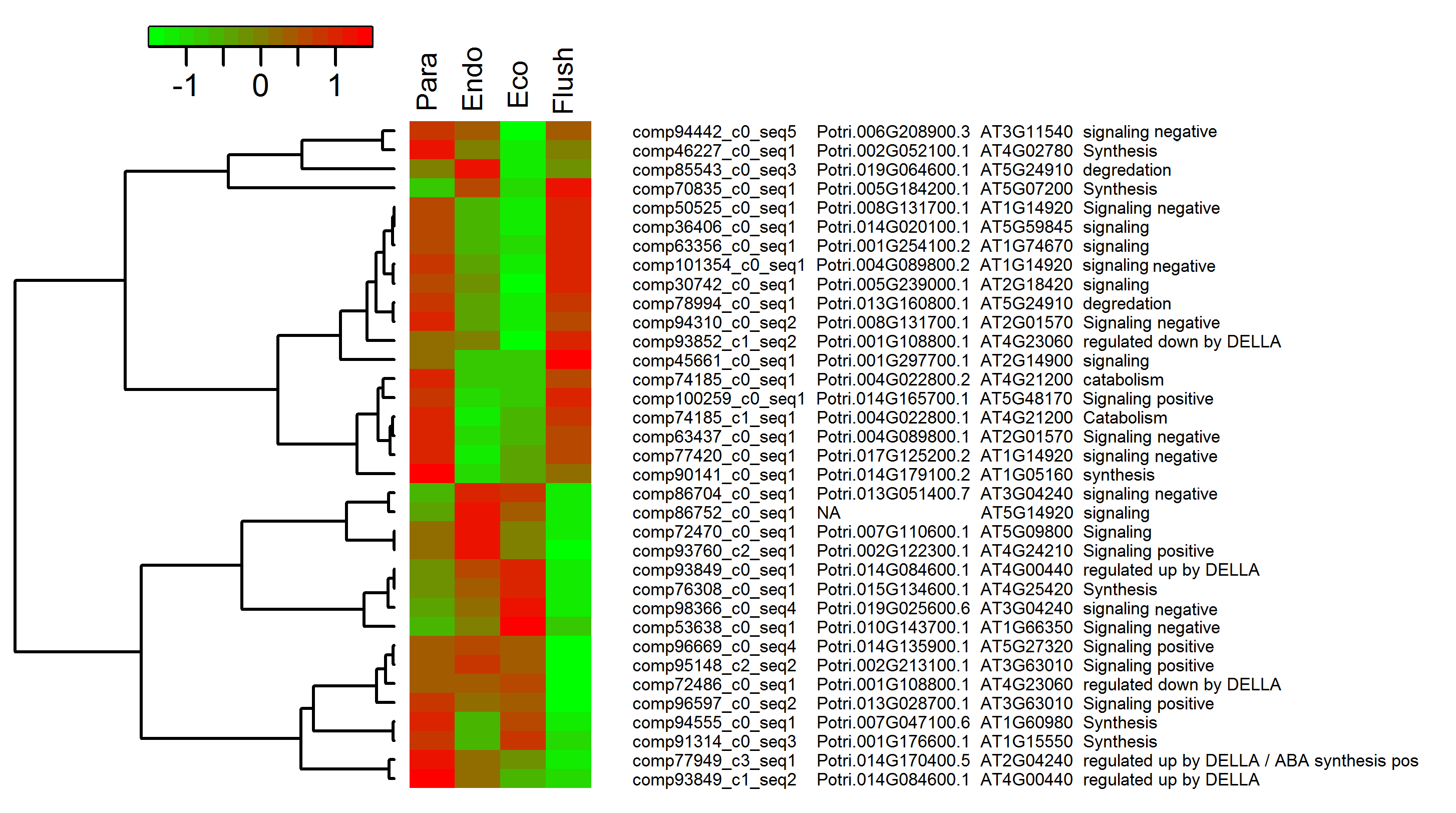


S2 Expression profiling analysis of gibberellin-associated differentially expressed genes. The information listed on the right of heat map are the transcripts’ name in this study, the transcript’s annotation by poplar protein database, the transcript’s annotation by *Arabidopsis* protein database, and hormone function. Red indicates high relative gene expression and green indicates low relative gene expression.


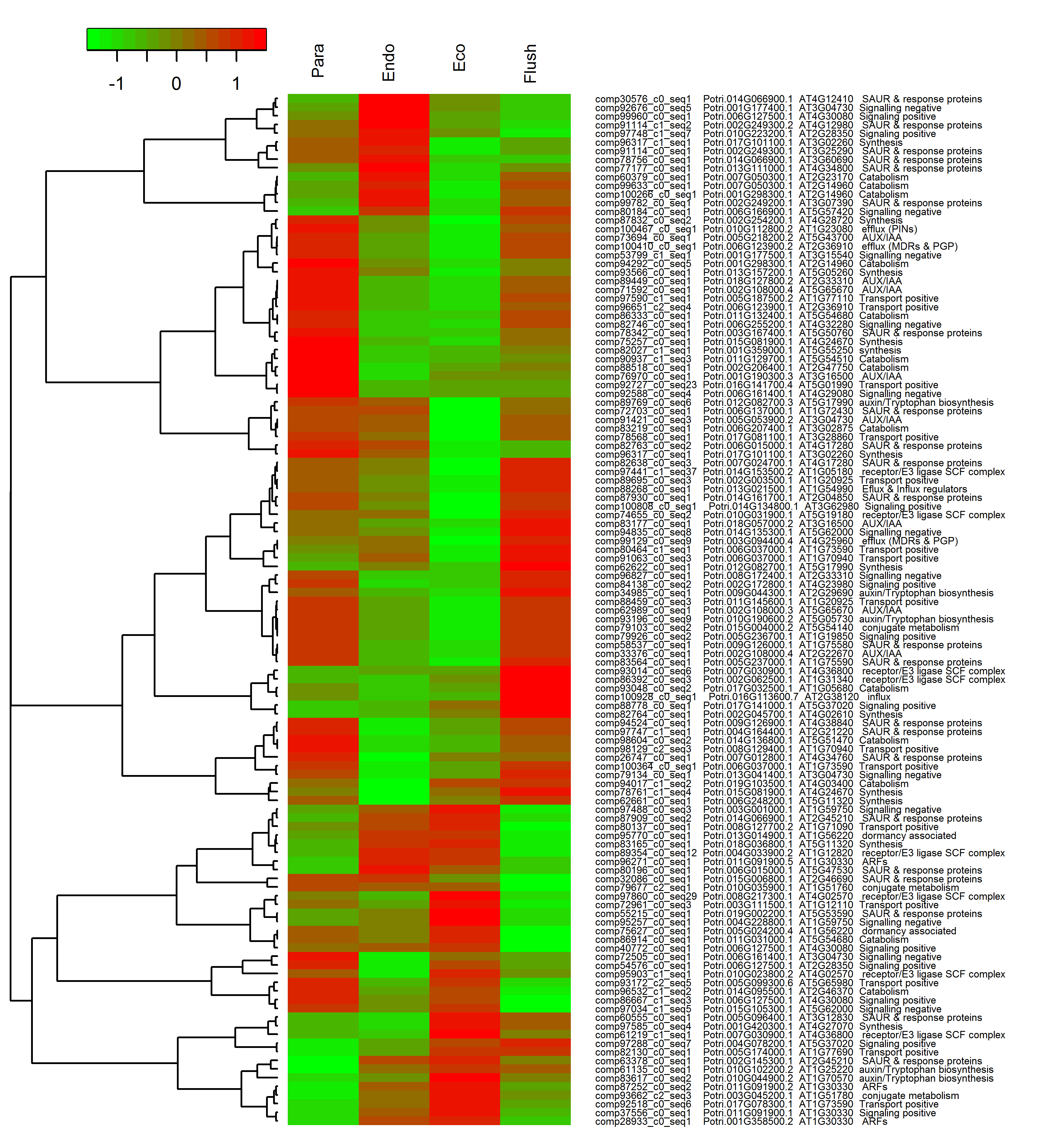


S3 Expression profiling analysis of auxin-associated differentially expressed genes. The information listed on the right of heat map are the transcripts’ name in this study, the transcript’s annotation by poplar protein database, the transcript’s annotation by *Arabidopsis* protein database, and hormone function. Red indicates high relative gene expression and green indicates low relative gene expression.


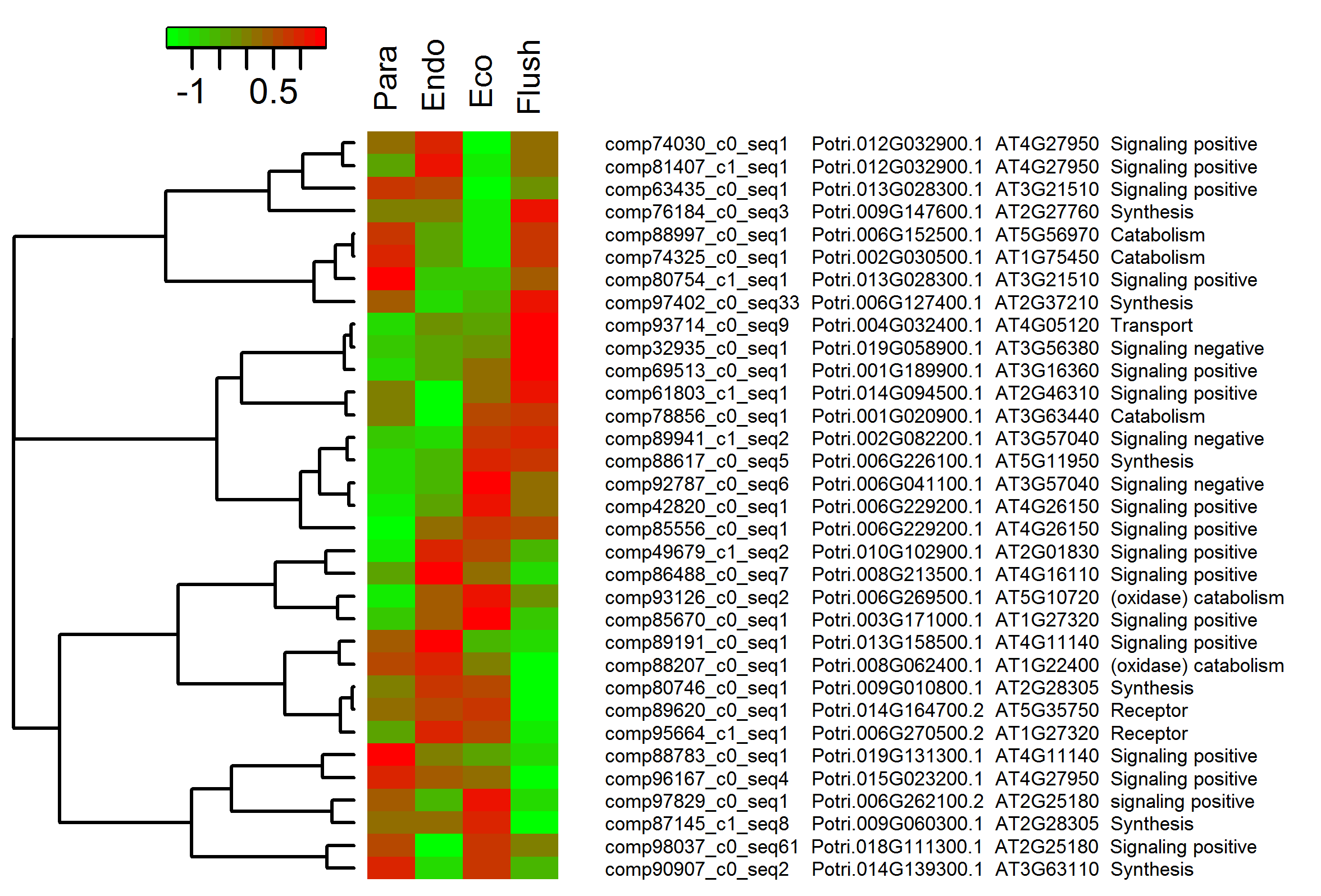


S4 Expression profiling analysis of cytokinin-associated differentially expressed genes. The information listed on the right of heat map are the transcripts’ name in this study, the transcript’s annotation by poplar protein database, the transcript’s annotation by *Arabidopsis* protein database, and hormone function. Red indicates high relative gene expression and green indicates low relative gene expression.


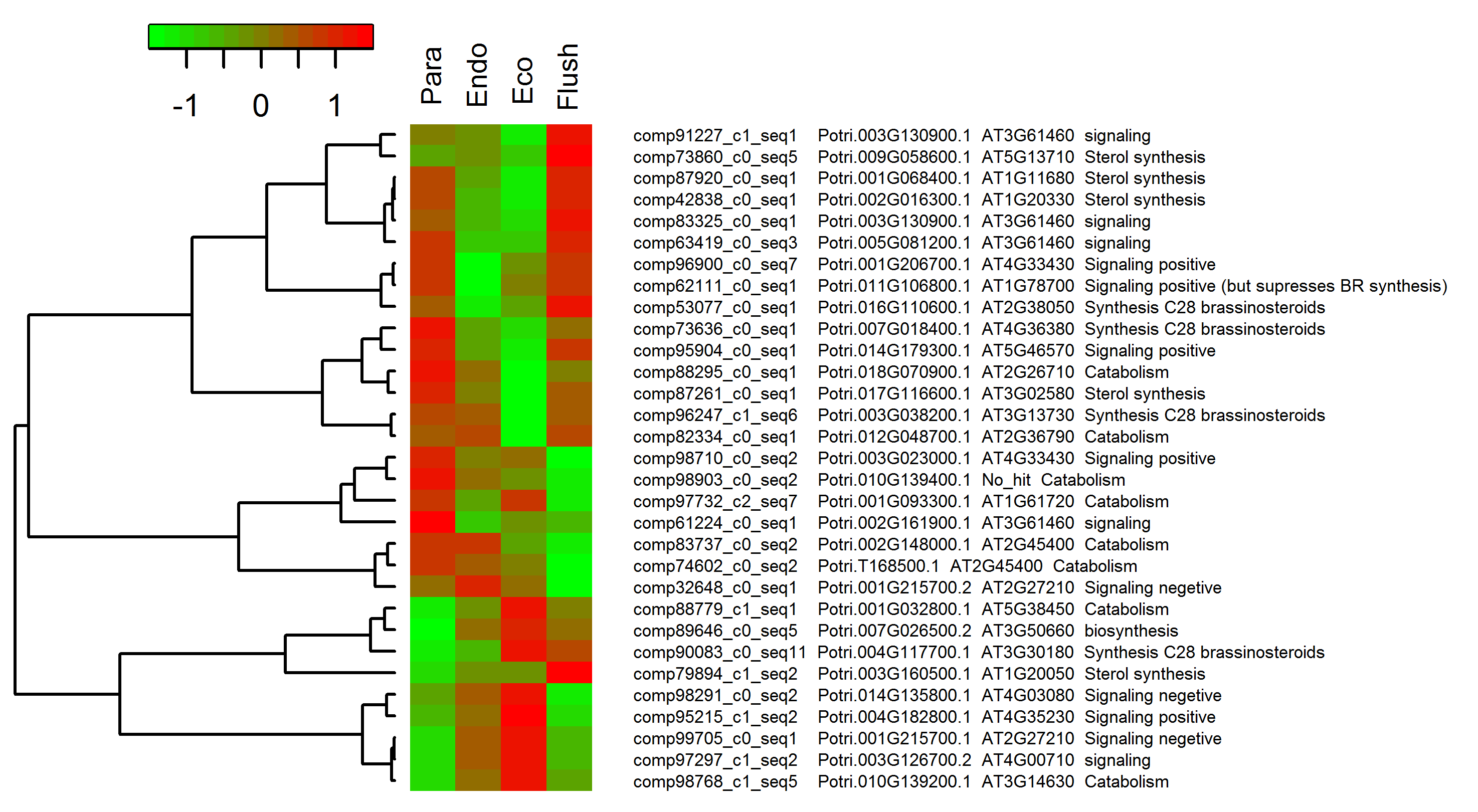


S5 Expression profiling analysis of brassinosteroid-associated differentially expressed genes. The information listed on the right of heat map are the transcripts’ name in this study, the transcript’s annotation by poplar protein database, the transcript’s annotation by *Arabidopsis* protein database, and hormone function. Red indicates high relative gene expression and green indicates low relative gene expression.


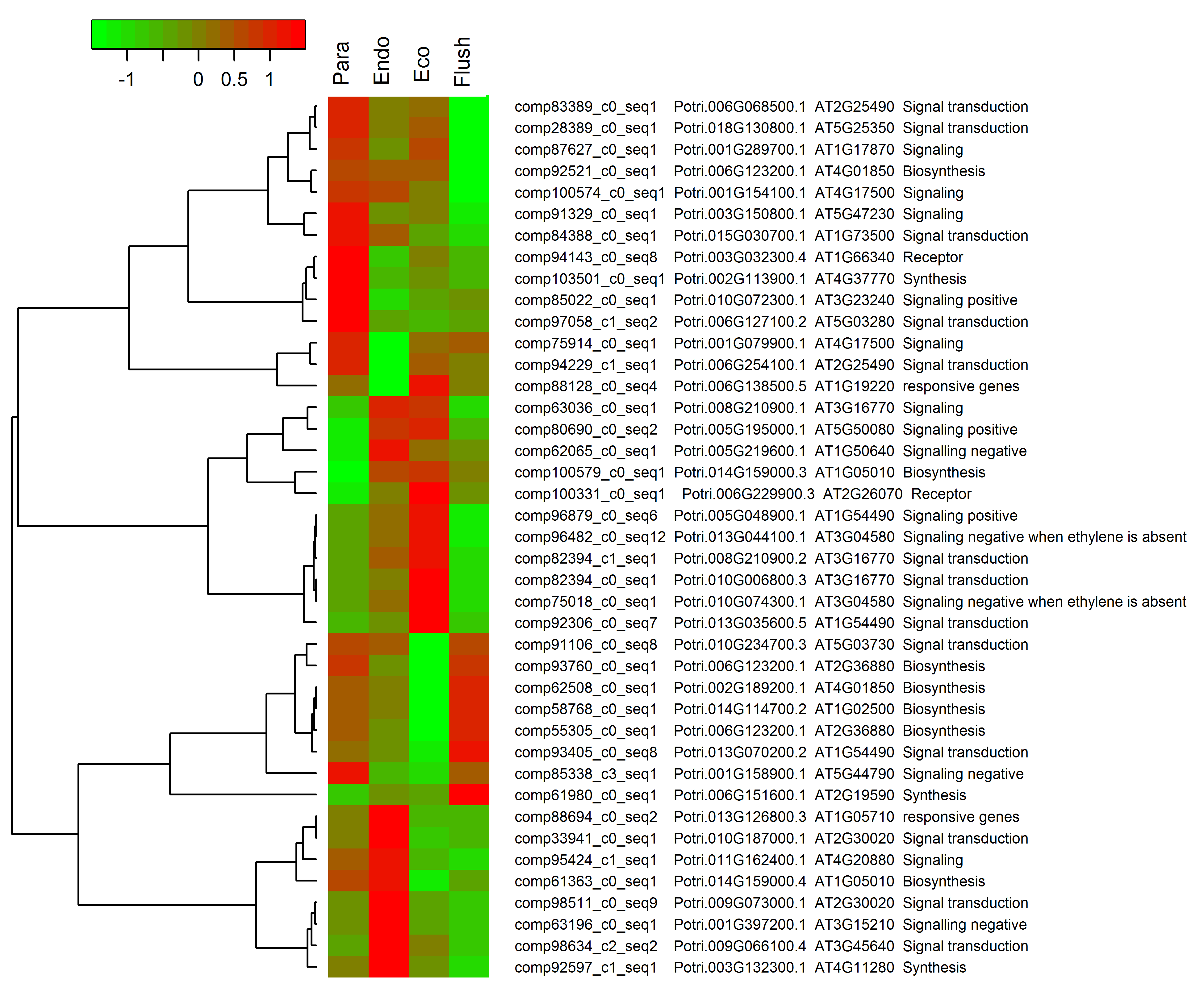


S6 Expression profiling analysis of ethylene-associated differentially expressed genes. The information listed on the right of heat map are the transcripts’ name in this study, the transcript’s annotation by poplar protein database, the transcript’s annotation by *Arabidopsis* protein database, and hormone function. Red indicates high relative gene expression and green indicates low relative gene expression.


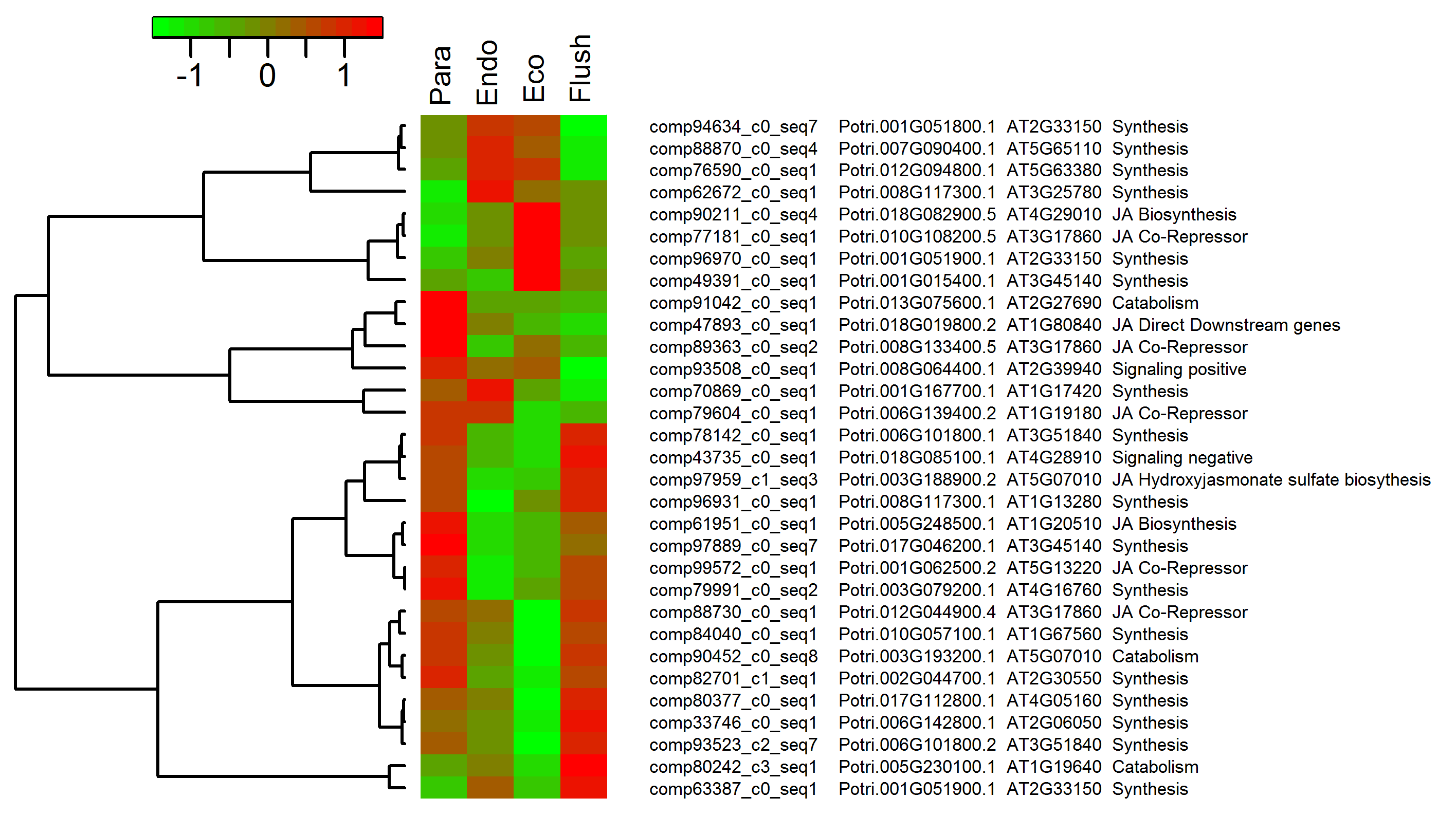


S7 Expression profiling analysis of jasmonic acid-associated differentially expressed genes. The information listed on the right of heat map are the transcripts’ name in this study, the transcript’s annotation by poplar protein database, the transcript’s annotation by *Arabidopsis* protein database, and hormone function. Red indicates high relative gene expression and green indicates low relative gene expression.
